# Paromomycin for the Treatment of Visceral Leishmaniasis in Sudan: A Randomized, Open-Label, Dose-Finding Study

**DOI:** 10.1371/journal.pntd.0000855

**Published:** 2010-10-26

**Authors:** Ahmed M. Musa, Brima Younis, Ahmed Fadlalla, Catherine Royce, Manica Balasegaram, Monique Wasunna, Asrat Hailu, Tansy Edwards, Raymond Omollo, Mahmoud Mudawi, Gilbert Kokwaro, Ahmed El-Hassan, Eltahir Khalil

**Affiliations:** 1 Institute of Endemic Diseases, University of Khartoum, Khartoum, Sudan; 2 Faculty of Medicine, Gedaref University, Gedaref, Sudan; 3 Drugs for Neglected Diseases initiative (DNDi), Geneva, Switzerland; 4 Centre for Clinical Research, Kenya Medical Research Institute (KEMRI), Nairobi, Kenya; 5 Addis Ababa University, Addis Ababa, Ethiopia; 6 London School of Hygiene and Tropical Medicine, London, United Kingdom; 7 Faculty of Pharmacy, University of Nairobi, Nairobi, Kenya; 8 Consortium for National Health Research (CNHR), Nairobi, Kenya; London School of Hygiene and Tropical Medicine, United Kingdom

## Abstract

**Background:**

A recent study has shown that treatment of visceral leishmaniasis (VL) with the standard dose of 15 mg/kg/day of paromomycin sulphate (PM) for 21 days was not efficacious in patients in Sudan. We therefore decided to test the efficacy of paramomycin for a longer treatment duration (15 mg/kg/day for 28 days) and at the higher dose of 20 mg/kg/day for 21 days.

**Methods:**

This randomized, open-label, dose-finding, phase II study assessed the two above high-dose PM treatment regimens. Patients with clinical features and positive bone-marrow aspirates for VL were enrolled. All patients received their assigned courses of PM intramuscularly and adverse events were monitored. Parasite clearance in bone-marrow aspirates was tested by microscopy at end of treatment (EOT, primary efficacy endpoint), 3 months (in patients who were not clinically well) and 6 months after EOT (secondary efficacy endpoint). Pharmacokinetic data were obtained from a subset of patients weighing over 30 kg.

**Findings:**

42 patients (21 per group) aged between 4 and 60 years were enrolled. At EOT, 85% of patients (95% confidence interval [CI]: 63.7% to 97.0%) in the 20 mg/kg/day group and 90% of patients (95% CI: 69.6% to 98.8%) in the 15 mg/kg/day group had parasite clearance. Six months after treatment, efficacy was 80.0% (95% CI: 56.3% to 94.3%) and 81.0% (95% CI: 58.1% to 94.6%) in the 20 mg/kg/day and 15 mg/kg/day groups, respectively. There were no serious adverse events. Pharmacokinetic profiles suggested a difference between the two doses, although numbers of patients recruited were too few to make it significant (n = 3 and n = 6 in the 20 mg/kg/day and 15 mg/kg/day groups, respectively).

**Conclusion:**

Data suggest that both high dose regimens were more efficacious than the standard 15 mg/kg/day PM for 21 days and could be further evaluated in phase III studies in East Africa.

**Trial Registration:**

ClinicalTrials.gov NCT00255567

## Author Summary

Visceral leishmaniasis (VL) is a parasitic disease transmitted through the bite of sandflies. The WHO estimates 500,000 new cases of VL each year, with more than 90% of cases occurring in Southeast Asia, East Africa, and South America. If left untreated, VL can be fatal. We had previously conducted a large multi-center study in Sudan, East Africa, to assess the efficacy of paromomycin (PM) alone or in combination with sodium stibogluconate. Clinical studies in India have shown that 15 mg/kg/day PM for 21 days was an effective cure. However, the same treatment regimen was not efficacious in two study sites in Sudan. Here, our aim was to assess two high-dose regimens of PM in Sudan: 15 mg/kg/day for 28 days and 20 mg/kg/day for 21 days. The results suggest that, at these total doses, PM is more efficacious than when given daily at 15 mg/kg for 21 days, and that high doses are required to treat VL in Sudan. Efficacy of 20 mg/kg/day PM for 21 days is currently being evaluated in a prospective, comparative phase III trial in East Africa.

## Introduction

According to the WHO estimates, visceral leishmaniasis (VL) is a parasitic disease that affects more than 500,000 people globally each year [1], and has a fatality rate of up to 100% if left untreated [2]. 90% of cases occur in five countries: India, Bangladesh, Nepal, Sudan, and Brazil [1], with the affected communities mostly located in remote regions of these endemic areas without ready access to treatment.

Although drugs (mainly antimonials such as sodium stibogluconate [SSG]) currently exist to treat this parasitic infection, their use has been limited because of high cost, toxicity, or development of parasite resistance [3]–[5]. A multi-center phase III study in India showed that PM is a very efficacious, affordable, and safe treatment [6], and is now registered for VL treatment in India.

In an effort to identify an effective treatment for VL in East Africa, we had previously initiated a multi-center phase III study in Sudan, Ethiopia, and Kenya comparing the efficacy of PM alone at the dose shown to be efficacious in India (15 mg/kg/day for 21 days) against SSG alone (20 mg/kg/day for 30 days) and against a combination treatment of SSG and PM (same dose of individual treatments but for 17 days). PM monotherapy did not show adequate efficacy, particularly in Sudan where parasite clearance was below 50% in patients at 6 months after end of treatment (EOT), and the study had to be prematurely stopped [7].

In the current study, we sought to find an efficacious dose of PM for the treatment of VL in Sudan and to explore possible reasons for the failure of the drug at the previous dose studied of 15 mg/kg/day for 21 days. In our previous study using this dose, conducted in 5 sites in Ethiopia, Kenya and Sudan, we found an overall end of treatment cure of 67.4% and 6-month post-treatment cure of 63.8% [7]. Cure at both sites in Sudan was below 50% [7]. The cure rate in this study of SSG was 92.2% at 6 months post-treatment [7].

Therefore a total dose increase of 33% was attempted through two possible regimens- an increased dose of 20 mg/kg for 21 days or a prolonged course of 15 mg/kg for 28 days. The former regimen has been evaluated in some clinical trials in India [8], [9]. There was no previous clinical experience with the 15 mg/kg dosage given for 28 days. The rationale was that the longer course of treatment would provide additional time for the patient's general condition to improve, and for their immunological response to develop, and that this might translate into a better clinical response without increasing the daily dosage.

## Methods

### Study objectives

The main objective was to assess the efficacy of two dosing regimens of PM monotherapy for the treatment of VL: 20 mg/kg/day for 21 days and 15 mg/kg/day for 28 days. Secondary objectives were to assess the safety of PM and compare the pharmacokinetic (PK) profiles of the two groups in a subset of patients.

### Participants

Patients with clinical symptoms and signs suggestive of VL and confirmed by visualization of parasites in bone-marrow aspirates were eligible for enrollment according to the National VL guidelines for Sudan for treatment and control. To be included in the study, patients had to: be between 4 and 60 years of age; be able to comply with the protocol (Protocols S1, S2 and S3); and provide written informed consent signed by themselves or by parents or legal guardians.

Patients were excluded from the study if they: had negative bone-marrow smears; were clinically contraindicated to having a bone-marrow aspirate; received any anti-leishmania drug in the past 6 months; had severe protein or caloric malnutrition (Kwashiorkor or marasmus); had previous hypersensitivity reaction to aminoglycosides; suffered from a concomitant severe infection, ie tuberculosis, HIV, or any other serious underlying disease (cardiac, renal, hepatic); suffered from other conditions associated with splenomegaly such as schistosomiasis; had previous history of cardiac arrhythmia or an abnormal electrocardiogram (ECG); were pregnant or lactating; or had pre-existing clinical hearing loss. If tuberculosis or schistosomiasis were suspected, these were screened through laboratory testing. Additionally, patients with the following laboratory values were excluded: hemoglobin less than 5 g/dL; white blood cell less than 10^3^/mm^3^; platelets less than 40,000/mm^3^; liver function test values more than three times the normal range; and serum creatinine values outside the normal range for age and gender.

### Study design

This was a two-arm, randomized, open-label, dose-finding study done at a single site in Sudan (Kassab Hospital, Ministry of Health, Gedaref State). This site participated in the previous study conducted on PM [7]. Eligible patients were randomly assigned to 20 mg/kg/day PM for 21 days (n = 21) or 15 mg/kg/day PM for 28 days (n = 21), and started a treatment regimen upon allocation to their treatment. Restricted-block randomization was done for the two groups. Randomization was done using sequentially numbered sealed envelopes that were prepared according to a centrally generated randomization list. Treatment was administered via daily intramuscular injection, and patients remained in the hospital for the duration of treatment. Patients were followed up at 3 and 6 months after treatment as outpatients. Parasitological assessments (bone-marrow aspirates only) were done at baseline, end of treatment (EOT), 3 months (only on patients who were not clinically well) and 6 months after treatment. Safety and clinical laboratory assessments were done at baseline, day 7 and day 14 of drug administration, EOT, and at 3 and 6 months follow-ups. These included a clinical assessment, (clinical symptoms, vital signs, weight, spleen and liver size), ECG, HIV testing (at baseline only), hemoglobin, white cell count, platelets, urea, creatinine, liver function tests (bilirubin, aspartate aminotransferase, alanine aminotransferase, alkaline phosphatase), urinalysis and audiometry. Audiometry was performed using a standardized procedure by site investigators who were trained by a qualified audiometrist and recorded as hearing levels in dB at 0.25, 0.5, 1, 2, 4 and 8 kHz frequencies [10]. All reported abnormal audiometric readings were reviewed by the audiometrist. An audiometric shift was defined in patients for whom there was one of the following: an increase in hearing level between baseline and EOT of ≥25 dB at ≥1 threshold frequency; an increase in hearing level between baseline and EOT of ≥20 dB at ≥2 adjacent threshold frequencies. Disabling hearing impairment was determined as an average of at least 41 dB across 0.5, 1, 2 and 4 kHz frequencies in adults (ages 15 years and above) and at least 31 db across 0.5, 1, 2 and 4 kHz frequencies in children (less than 15 years of age) [10].

Parasitology slides were prepared from bone-marrow aspirates, read, and reported according to a standardized, approved WHO method [11], [12]. Standardised parasitology readings were done from freshly prepared bone-marrow aspirates taken directly from the patients to the laboratory. Slide fields were examined and counted for parasites under oil emersion 100× magnification for 30 minutes (timed) before being declared negative (absence of parasites on microscopy slide). All parasitology was performed by a trained laboratory technician.

For the PK analysis, the first six consenting patients weighing 30 kg or more were selected from each treatment group and had additional venous blood and urine samples on day 1 and day 14 in the 20 mg/kg/day group, and on day 1 and day 26 in the 15 mg/kg/day group. The timing for blood sampling was 0 (before treatment) and at 0.25, 0.5, 1, 2, 4, 6, 8, 12, and 24 hours after administration of the drug, and at 0–2, 2–4, 4–6, 6–8, 8–12, and 12–24 hours after administration of the drug for urine sampling.

### Ethics statement

The trial was done in accordance with the Declaration of Helsinki (2002 version) for the conduct of research on human subjects and followed the International Committee for Harmonization guidelines for the conduct of clinical trials. All trial site personnel received relevant training in Good Clinical Practices.

The Ethics Committee of the Institute of Endemic Diseases, University of Khartoum, and the Directorate of Health Research, Federal Ministry of Health, Sudan approved the study protocol (July 8, 2005), which was submitted as a protocol amendment (Protocol S3) to our previous study [7].

All participants or their parents or legal guardians gave their written informed consent before entry into the trial. Children were included in this study because they represent more than 50% of VL cases in this endemic area, and were included in the PK sampling if they met the weight criteria (>30 kg). This study was registered at ClinicalTrials.gov (registration number NCT00255567).

### Treatment

The study medication was 1 g/2mL paromomycin sulphate (Gland Pharma, India). Doses in the study groups were 20 mg/kg/day paromomycin sulphate (equivalent to 15 mg/kg/day of paromomycin base) and 15 mg/kg/day paromomycin sulphate (equivalent to 11 mg/kg/day paromomycin base). The rescue medication was AmBisome (a liposomal formulation of amphotericin B, Gilead, USA), which was reconstituted according to the manufacturer's instructions for a dosage of 3 mg/kg/day for 10 days.

### Outcome measurements

#### Efficacy

The primary efficacy endpoint was cure (parasite clearance) at EOT (i.e., on days 22 and 29 for 20 mg/kg PM and 15 mg/kg PM, respectively). The secondary efficacy endpoint was parasite clearance at 6 months follow-up.

Efficacy was reported as the number and percentage of randomized patients in whom no parasites were detected by arm at each endpoint. Rescue medication before an endpoint indicates treatment failure at that endpoint. Clinical and biological parameters, such as temperature, weight, spleen size and hemoglobin, were used by clinicians to decide each patient's response to treatment and whether rescue medication was indicated. Relapse at 3-month follow-up was defined as patients with no parasites detected at EOT but in whom parasites were seen at 3-month follow-up. Slow response to treatment was defined as the presence of parasites at EOT but not at 6 months in the absence of rescue medication. Therefore, a treatment failure at initial test of cure (EOT) that did not receive rescue medication was defined as a slow responder so long as there was parasite clearance at 6-month follow-up.

#### Safety

Safety analyses were based on the number of adverse events (AEs) that occurred during the study, including changes of pre-defined magnitudes in audiometric measurements (a known side-effect of aminoglycosides [13]) and abnormal clinical laboratory or vital sign measurements. AEs were rated for seriousness, severity, and causality by the site investigator and were coded using the Medical Dictionary of Regulatory Activities (MedDRA), version 10.0.

Audiometric shift and disabling hearing impairment were defined according to an earlier phase III trial [6] and WHO guidelines [10].

#### Pharmacokinetics

For the PK study, only patients with body weight of more than 30 kg were included. PM was assayed using high-performance liquid chromatography, as described previously [14] with some modifications.

#### Statistical methods

The study was not powered to detect differences between the groups; indeed, the study objective was to assess whether improved efficacy without increasing risk could be obtained by using either a higher dose or an increased treatment duration of PM. A precision estimate for 21 patients would allow estimation of efficacy such that the lower bound of the 95% confidence interval around the efficacy estimate will be at least 60% for 17 or more treatment successes (efficacy at least 81%).

Analyses were carried out using Stata, version 10 (Stata Corporation, College Station, Texas). Analyses were done on the intention-to-treat (ITT) population. In case of missing data, efficacy analyses were by complete-case analysis, excluding patients with missing data, and by worst-case analysis, where missing efficacy data are assumed to be treatment failures. To account for different treatment durations and avoid overestimating harm in the longer treatment duration group, the treatment-emergent adverse event (TEAE) rate was calculated, per group, as the total number of adverse events divided by the total person-time at risk [15], [16]. The total person-time at risk for TEAE in each group was defined as the number of randomized patients multiplied by the treatment duration in days plus 30 days (51 days in the group of PM 20 mg/kg for 21 days and 58 days in the group of PM 15 mg/kg for 21 days).

## Results

### Study population

**Figure 1:**
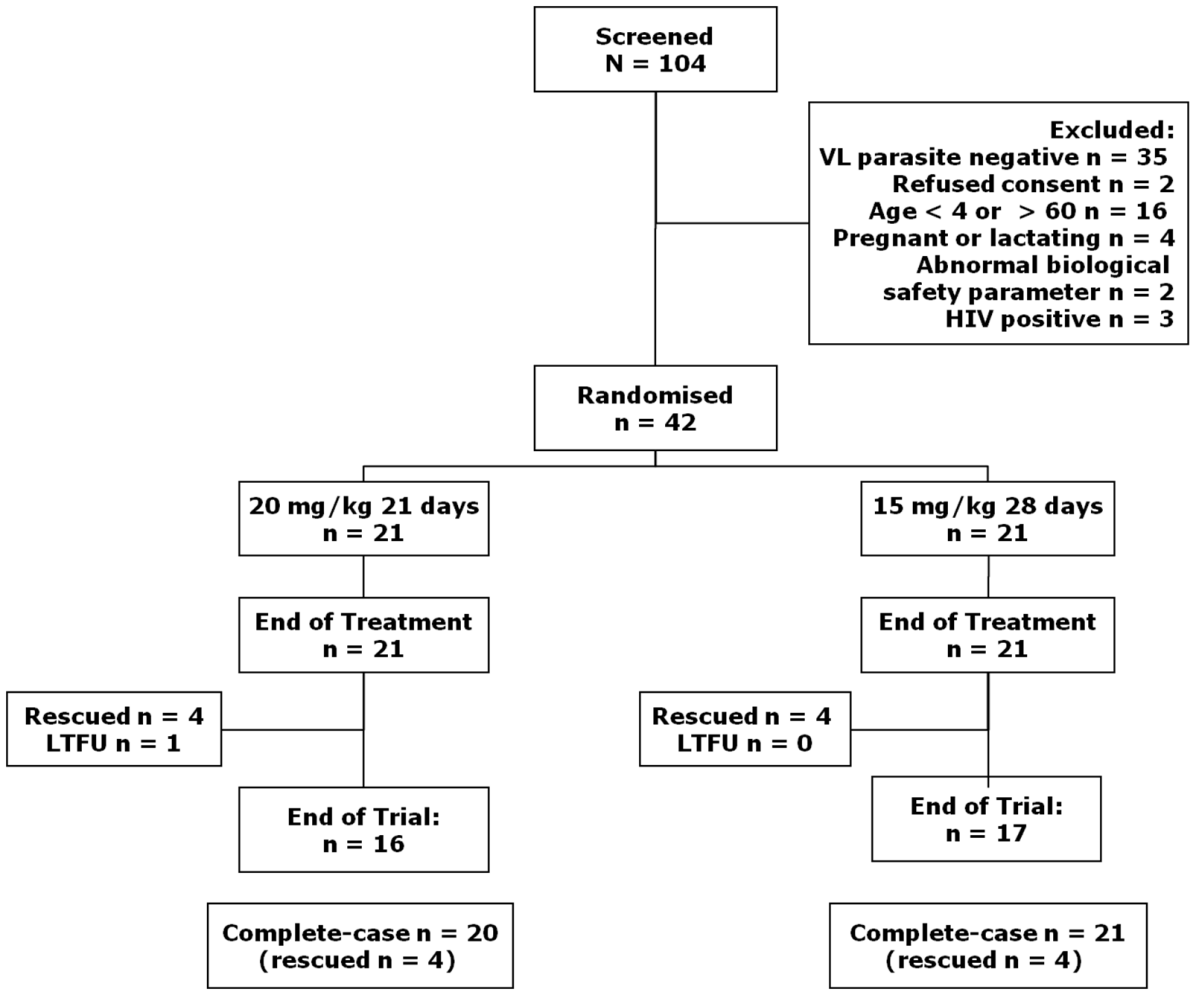
Flowchart of patients.

104 patients with suspected VL were screened for entry into this study. Of these, 42 patients were enrolled in the study (21 per group; figure 1). Demographics and baseline characteristics were similar in the two groups (table 1). One patient in the 20 mg/kg/day PM group was considered lost by the 6-month follow-up. The first patient was recruited in October 2005 and the last patient followed-up in October 2006.

**Table 1:** Baseline characteristics.

	20 mg/kg/day PM for 21 days (N = 21)	15 mg/kg/day PM for 28 days (N = 21)
**Age (years)**	19.4 (12.3)	17.7 (9.8)
4–14 years	10 (47.6%)	8 (38.1%)
15–60 years	11 (52.4%)	13 (61.9%)
**Sex (male)**	16 (76.2%)	19 (90.5%)
**Nutritional status** *		
Severely underweight	2 (9.5%)	0
Underweight	10 (47.6%)	13 (61.9%)
Normal	9 (42.9%)	8 (38.1%)
**Weight**	36.5 (16.5)	40.0 (16.8)
**Temperature (°C)**	38.9 (0.6)	39.0 (0.6)
**Heart rate (beats/min)**	106.2 (13.2)	105.4 (12.1)
**Spleen size (cm)**	5.9 (3.3)	5.5 (3.6)
**Liver size (cm)**	2.9 (3.0)	3.0 (2.0)
**Systolic BP (mm Hg)**	100.7 (12.1)	99.0 (12.6)
**Diastolic BP (mm Hg)**	61.9 (9.7)	59.8 (10.4)
**Log scale parasite count,**		
6+	4 (19.1%)	1 (4.8%)
5+	0	0
4+	1 (4.8%)	2 (9.5%)
3+	1 (4.8%)	2 (9.5%)
2+	1 (4.8%)	4 (19.1%)
1+	14 (66.7%)	12 (57.1%)
**Hemoglobin (g/dL)**	7.6 (1.4)	7.7 (0.9)
**White-cell count (×10^3^/µL)**	3.1 (1.2)	2.8 (0.9)
**Platelets (×10^3^/µL)**	311.6 (165.8)	251.7 (89.5)
**AST (U/L)**	18.8 (5.3)	19.9 (5.2)
**ALT (U/L)**	18.1 (4.5)	18.8 (4.7)
**Bilirubin (µmol/L)**	12.0 (5.8)	14.0 (10.4)
**BUN (mmol/L)**	9.7 (3.1)	9.5 (3.8)
**Creatinine (µmol/L)**	55.1 (15.8)	64.4 (19.4)

Data are mean (SD) or n (%).

ALT, alanine aminotransferase; AST, aspartate aminotransferase; BP, blood pressure; BUN, blood urea nitrogen.

*Based on Weight for Height (WHO child growth standards) if age <6 years) and BMI for Age if age 6–19 years; defined as severely underweight if z-score<−3SD; underweight if −3SD≤z-score <−2SD; normal if −2SD≤z-score<+1SD; and BMI if age >19 years: defined as severely underweight if <16, underweight: 16.0–18.4, normal: 18.5–24.9.

### Efficacy

Data were available for all patients at EOT (figure 1). 18 patients in the 20 mg/kg group and 19 in the 15 mg/kg group had parasite clearance at EOT, indicating an efficacy of 85.7% (95% CI: 63.7% to 97.0%) and 90.5% (95% CI: 69.6% to 98.0%), respectively (table 2).

**Table 2:** Complete-case efficacy analysis.

	20 mg/kg/day PM for 21 days (N = 21)	15 mg/kg/day PM for 28 days (N = 21)
**End of treatment**	18/21 (85.7%)95% CI: 63.7–97.0%	19/21 (90.5%)95% CI: 69.6–98.8%
**6-month follow-up** *	16/20 (80.0%)95% CI: 56.3–94.3%	17/21 (81.0%)95% CI: 58.1–94.6%

Data are n (%). CI, confidence interval.

*One patient lost to follow-up at 6 months in the 20 mg/kg group: worst-case efficacy = 16/21 (76.2%, 95% CI: 52.8–91.8%).

At 3-months follow-up, two patients had relapsed in the 20 mg/kg/day for 21 days regimen and three in the 15 mg/kg/day for 28 days regimen; however, there were no additional relapses at 6 months. At 6-months follow-up, the complete-case analysis efficacy in both groups was similar (80.0% in the 20 mg/kg/day group versus 81.0% in the 15 mg/kg/day group) (table 2). All treatment failures were given rescue medication. An exception was one patient in the 20 mg/kg/day PM group who was parasite positive at EOT but clinically responded. This patient was lost to follow-up at 6 months, leading to a lower efficacy estimate in the worst-case analysis (table 2). There was one slow responder (ie, parasite-positive patient at EOT, but clinically well and ultimately recovered) in the group treated with 15 mg/kg/day PM for 28 days.

### Safety

PM was well tolerated in this study. 48 AEs were reported in total; 20 in the 20 mg/kg for 21 days group and 28 in the 15 mg/kg for 28 days group (table 3), and none was regarded as serious. This gives an AE rate of 0.05 per person-day on treatment in both groups. All AEs, except diarrhea and malaria, were judged to be related to the treatment. The most frequent AE was injection site pain (n = 33). Audiometric shifts were seen in five patients at EOT (n = 3 in the 15 mg/kg group and n = 2 in the 20 mg/kg group), but completely resolved by 6 months follow-up. Disabling hearing impairment, detected at EOT, which improved but persisted at 6 months (ie still met the criteria for audiogram shift), occurred in one patient in the 20 mg/kg group.

**Table 3:** Non-serious adverse events.

Events	20 mg/kg/day PM for 21 days (N = 21)	15 mg/kg/day PM for 28 days (N = 21)
**Diarrhea**	1 (4.8%)	0
**Injection site pain**	14 (66.7%)	16 (76.2%)
**Abnormal audiogram**	3 (14.3%)	3 (14.3%)
**Malaria**	1 (4.8%)	1 (4.8%)
**Acute otitis media**	0	1 (4.8%)
**PKDL**	1 (4.8%)	4 (19.0%)
**Epitaxis**	0	1 (4.8%)
**Cholestatic jaundice**	0	1 (4.8%)
**Jaundice**	0	1 (4.8%)
**Total**	**20**	**28**

Data are n (%) of AEs reported. PKDL, Post-kala-azar dermal leishmaniasis.

### Pharmacokinetics

Although six patients from each group should have taken part in the PK study, only data from three patients in the 20 mg/kg/day PM group and six in the 15 mg/kg/day PM group were obtained. Only one patient was a child (age of 12 years and weight of 39 kg in the 15 mg/kg group). The others were aged between 17 and 28 years.

**Figure 2:**
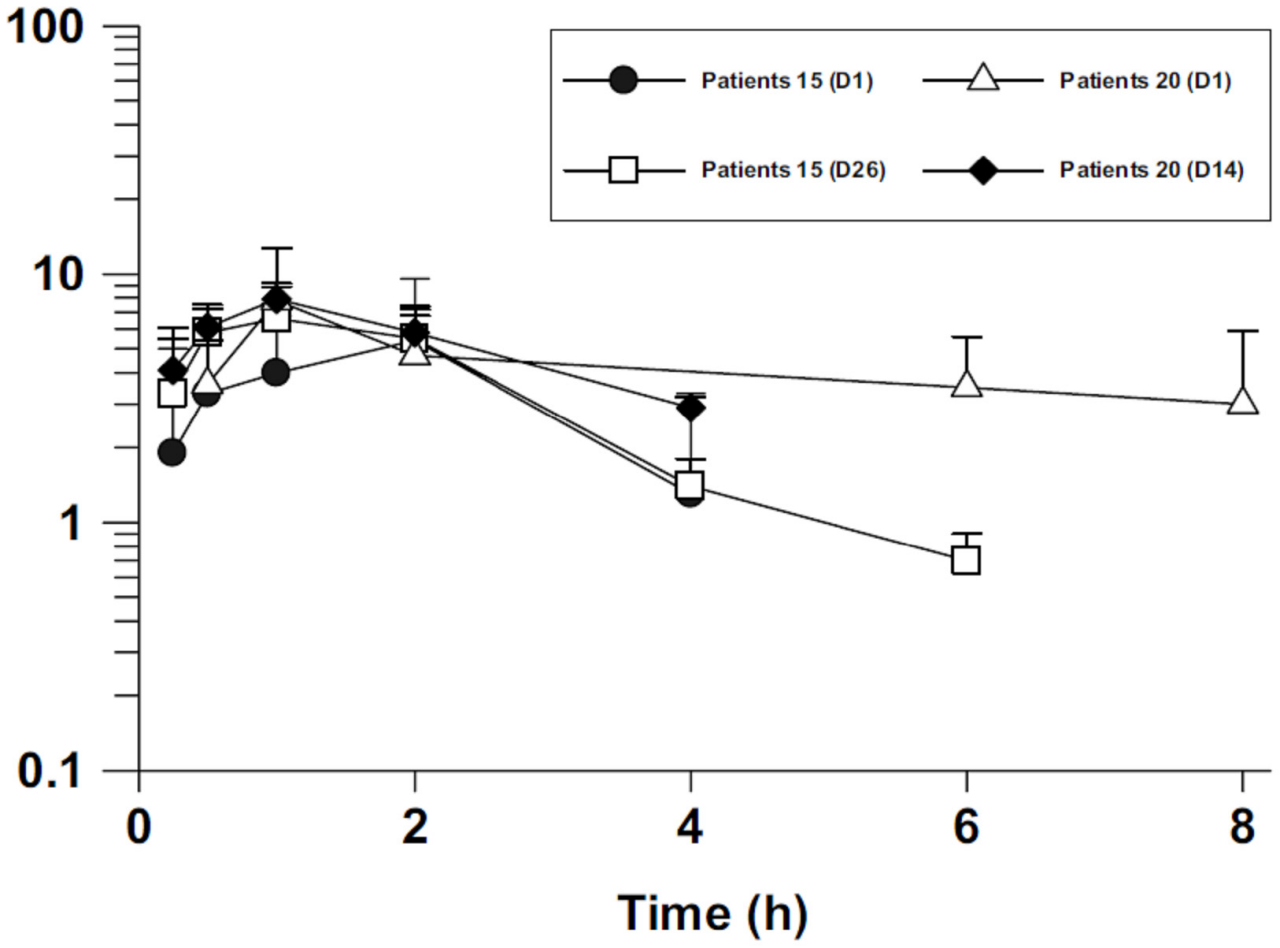
Semi-log plot of mean (SD) paromomycin (PM) concentrations (µg/mL) versus time (hours) after a single intramuscular injection. Plasma PM concentrations on day 1 (D1), day 14 (D14), and day 26 (D26) for VL patients who received either 15 mg/kg (n = 6) or 20 mg/kg PM (n = 3).

Mean plasma PM concentrations at the earlier time points were similar between the two treatment groups (figure 2). Nevertheless, the peak mean plasma PM concentration on day 1 was slightly higher in the 20 mg/kg/day group compared with that in the 15 mg/kg/day group (7.8±4.9 µg/mL versus 5.6±4.2 µg/mL). Six hours after administration, PM was not detected in the plasma of patients receiving 15 mg/kg/day PM but was seen at concentrations slightly lower than peak in the plasma of patients receiving 20 mg/kg/day PM (figure 2).

## Discussion

To date, the standard treatment for VL in East Africa still consists of antimonials. This study is part of the first large-scale multi-centre clinical trial to assess the efficacy of PM for the treatment of VL for the East African region. The initial study [7] showed poor efficacy results when 15 mg/kg/day PM was administered for 21 days to VL patients. This finding is in contrast to an earlier phase III study in India [6]. The results of this study show that increasing the total dose of PM from 15 mg/kg/day for 21 days to 15 mg/kg/day for 28 days or 20 mg/kg/day for 21 days improves efficacy in VL patients in Sudan. However, it should be cautioned that the results found in this study apply to one site only and might not apply to the whole East African region.

Although efficacy is normally assessed as parasite clearance at 6 months in trials for VL, in this study we chose to use parasite clearance at EOT as the primary endpoint because a chance of loss to follow-up of just a few patients would significantly affect the result. In addition to the small sample size, another potential limitation is the use of bone-marrow aspiration for diagnosis and test of cure. However, spleen aspiration remains contraindicated in rural hospitals in Sudan, making bone marrow the best viable alternative.

At 6 months after treatment, efficacy was 80.0% (95% CI: 56.3% to 94.3%) and 81.0% (95% CI: 58.1% to 94.6%) in the 20 mg/kg/day and 15 mg/kg/day groups, respectively, compared with less than 50% (in Sudan) at 6 months observed in the previous study [7]. This result shows that efficacy improved to levels closer to those obtained in trials in India (∼95%) [6].

Serious safety issues that would limit the evaluation of PM at high doses were not identified in this study. Otoxicity, which has been seen as a transient side-effect of PM in other studies [6], was also identified as a potential issue in this study because one patient had audiometric shift at 6 months. This shift occurred at high frequencies, as expected with aminoglycosides [6]. We suggest that this adverse event needs to be monitored closely in subsequent studies.

PK analyses showed that peak plasma PM concentration occurred 1–2 hours after administration and suggest that, at the high daily dose of 20 mg/kg, elevated plasma PM concentrations may be maintained for a longer period of time (up to 8 hours). Unpublished data (Mahmoud Mudawi, personal communication) of PM administration (15 mg/kg) to healthy Sudanese volunteers showed peak PM plasma concentrations similar to those in American volunteers who received a similar dose [14]. Sudanese VL patients had a much lower plasma concentration (30–40%) than that of healthy Sudanese (19.5±7.6µg/mL; n = 6) and American volunteers. Therefore, Sudanese VL patients may have different PK characteristics from both Sudanese and American healthy volunteers, and Indian VL patients. However, PK data were very limited and derived from only a small subset of patients. A PK study with more patients is currently underway as part of the larger phase III study.

Even though interpretation of our results is limited because of the small sample size, we identified what seems to be a more efficacious dose of PM than the one previously used in Sudan [7]. A meeting of the principal investigators was held to discuss the PM efficacy and PK dose-finding results. The group chose to use in the large multi-center phase III study, a dose of 20 mg/kg/day for 21 days for a comparison with the previously used doses of SSG and SSG and PM in combination. Our initial study [7] showed that efficacy of PM can vary greatly between geographical regions, and in addition to this study, suggests that different doses may be required to obtain similar levels of efficacy. If confirmed, these results emphasize the importance of considering regional differences in the treatment of VL and show that drugs of proven efficacy in Asian patients might not have the same efficacy in African patients.

## Supporting Information

Checklist S1 CONSORT checklist.
(0.19 MB DOC)

Protocol S1 Dose-ranging CSR.
(0.56 MB PDF)

Protocol S2 Clinical trial protocol.
(0.23 MB PDF)

Protocol S3 Protocol amendment.
(0.06 MB PDF)
