# Supplementary material for: Paromomycin for the Treatment of Visceral Leishmaniasis in Sudan: A Randomized, Open-Label, Dose-Finding Study
Source: PLoS Negl Trop Dis. 2010 Oct 26;4(10):e855. doi: 10.1371/journal.pntd.0000855 (PMC2964291; doi:10.1371/journal.pntd.0000855)
Supplement: Protocol S1 — Dose-ranging CSR. (0.56 MB PDF) [file pntd.0000855.s002.pdf]

Clinical Study Report LEAP0104 - Part II

A MULTICENTRE COMPARATIVE TRIAL OF EFFICACY AND SAFETY OF SODIUM STIBOGLUCONATE VERSUS PAROMOMYCIN VERSUS COMBINATION OF SODIUM STIBOGLUCONATE AND PAROMOMYCIN AS THE FIRST LINE TREATMENT FOR VISCERAL LEISHMANIASIS (VL) IN ETHIOPIA, KENYA AND SUDAN - PART II

Amendment 5: PK Assessment in 42 patients with 2 PM treatment regimens in Kassab, Sudan

**Protocol number**	LEAP 0104 Amendment 5 dated 8 July 2005
**Name of product(s)**	Paromomycin sulphate
**Indication**	acute, symptomatic visceral leishmaniasis (VL)
**Phase**	II
**Study Design**	Randomised, open, parallel group trial of two dose regimens (33% increase in total dose administered compared to Part I of the trial) of paromomycin sulphate (PM)<br>PM 20 mg/kg/day for 21 days, (increase in daily dosage from 15 to 20 mg/kg) and PM 15 mg/kg/day for 28 days (additional 7 days treatment) in adults (maximum age 60 years) and children (minimum age 4 years) with parasitologically proven, symptomatic, acute VL.<br>Patients were treated as hospital in-patients for the duration of treatment and followed up for six months post-treatment as out-patients
**Sponsor**	DND*i*, 15 Chemin Louis-Dunant<br>1202 Geneva, Switzerland<br>Tel. + 41 (0) 22 906 9230
**Co-sponsor**	none
**Principal investigator**	Dr Ahmed Musa Mudawi,<br>Institute of Endemic Diseases, University of Khartoum, Sudan

	**Date**
**Study Initiation:**	October 2005
**Early study termination:**	Not applicable

Study completion: October 2006 (last patient last visit)

Study Report Date: December 2009

This study was performed in compliance with Good Clinical Practices, including the archiving of essential documents

2 SYNOPSIS

This report is **part II** of the trial LEAP 0104;

- **Part I:** initial 3 arm parallel group trial (prematurely stopped) using the planned dose paromomycin sulphate (PM) 15 mg/kg/day x 21 days (n=135) and the two other comparative arms sodium stibogluconate (SSG) (n=135) and SSG+PM (n=135) See Clinical Trial Report LEAP 0104 part I
- **Part II: dose ranging study (protocol LEAP 0104 amendment #5 dated 8th July 2005) comparing PM 15 mg/kg for 28 days and PM 20 mg/kg for 21 days.**
- **Part III:** 3 arm comparative trial in which patients receiving the PM monotherapy treatment receive 20 mg/kg PM for 21 days. The comparator SSG (20 mg/kg/day for 30 days) arm and the shorter course combination (PM 15 mg/kg/day + SSG 20 mg/kg/day for 17 days) treatment arms remained unchanged from the original protocol. To be reported subsequently.

The phase III, randomised, controlled, 3 arm, parallel group, comparative trial LEAP 0104 was designed to evaluate the efficacy and safety of PM as a potential new treatment for acute visceral leishmaniasis (VL) in Sudan, Ethiopia and Kenya.

It became clear six months after starting recruitment into the 3 arm trial that at both trial sites in Sudan, efficacy in the PM monotherapy arm at a dosage of 15 mg/kg /day for 21 days was inadequate, with only 42% of patients clear of parasites at test of cure. It could therefore be anticipated that the definitive cure at six months follow up, the primary endpoint of the trial, would be even lower.

Table 2-1 Complete Parasite Clearance (Test of Cure) by site and treatment arm (LEAP 0104 Part I) at End of Treatment

	Paromomycin* (%)	Sodium Stibogluconate** (%)	Combination† (%)	P-Value
**Um El Kher**	10 / 30 (33.3)	26 / 30 (86.7)	25 / 30 (83.3)	< 0.001
**Kassab**	9 / 15 (60.0)	14 / 15 (93.3)	12 / 15 (80.0)	0.113
**Kenya**	11 / 11 (100)	10 / 10 (100)	10 / 11 (90.9)	1.00
**P-Value***	< 0.001	0. 571	.981	

*15 mg/kg/day administered for 21 days, **20mg/kg/day for 30 days, †SSG 20 mg/kg/day+ PM 15 mg/kg/day for 17 days, P-value from Fisher's exact test

Conversely initial results from Kenya were promising, albeit in a small sample, with results similar to those achieved in India. No data were yet available from the two Ethiopian sites.

Furthermore, it had been established that this was not a trial site-related effect, in that

poor results had been obtained from two different sites in Sudan, with different trial teams that were more than 100 km distant from each other (Um El Kher and Kassab).

Re-analysis of drug retrieved from the first trial site had established that the drug remained of good quality and within its technical specification, and is therefore robust under field transport and storage conditions.

It was therefore assumed that the poor efficacy was either related to patient-specific characteristics of the Sudan population or to decreased susceptibility of the parasite to PM.

A decision was made by LEAP to stop recruitment in Sudan prematurely but to continue recruitment at other sites (Kenya and Ethiopia) until they had completed initial cohorts of patients (n=45 at each site) and meanwhile to carry out a dose ranging study in Sudan to see if it was possible to improve efficacy results in this challenging environment.

The two arms in this sub-study received increased total PM dosage over the course of treatment by 33%, either by increasing the daily dosage from 15 mg/kg to 20mg/kg but maintaining the treatment duration at 21 days, or by increasing the treatment duration from 21 to 28 days but keeping the daily dosing at 15 mg/kg. Twenty-one patients were recruited to each arm. Pharmacokinetics (PK) were performed on a subset of patients, key points will be described within this report and the full PK report can be found in Section 15.1.8.

This was essentially a phase II dose-ranging study and therefore not powered to show a statistical difference between arms, but to explore the possibility for improved efficacy. The results indicated that it was possible to improve efficacy by a clinically significant margin by increasing the total dosage of PM administered. The two arms were similar in terms of both safety and efficacy based on the small sample size of 21 patients per arm with test of cure at the end of treatment (EOT) in the range 85-90% for both treatments.

On the basis of these results, and in accordance with DND*i* VL strategy to find 'field-adapted' treatments of shorter duration, it was decided by the LEAP group, and ratified by DND*i* scientific advisory committee (SAC), to carry forward the 20 mg/kg 21day PM schedule and resume the original phase III, 3 arm comparative trial with this increased PM dosage, the other two arms being unchanged from the original protocol. See protocol amendments # 6, 8, 9 and 10. The trial resumed in November 2006 and will be reported subsequently in Clinical Trial Report LEAP 0104 Part III.

**Name of Sponsor/Company**: Drugs for Neglected Diseases initiative (DND*i*), Geneva, Switzerland	Individual Study Table Referring to Part of the Dossier	(For National Authority Use only)
**Name of Finished Product**: Paromomycin (PM)		Volume:
**Name of Active Ingredient**: Paromomycin sulphate		Page:

**Investigators**: Dr Ahmed Musa Mudawi, Institute of Endemic Diseases, University of Khartoum, Sudan	
**Study centre(s)**: Ministry of Health Hospital Kassab, Gedaref State, Sudan	
**Publication (reference)**: None	
**Studied period (years)**:<br>(date of first enrolment) October 2005<br>(date of last completed) October 2006	**Phase of development**: II
**Objectives**: The primary objective of this study was to evaluate efficacy of PM in two dosing regimens (15 mg/kg/day for 28 days vs. 20 mg/kg/day for 21 days) for the treatment of visceral leishmaniasis (VL) in East Africa	
**Methodology**: A single-centre, randomized, open two-arm study. The primary endpoint was parasite clearance at end of treatment (EOT)	
**Number of patients (planned and analysed)**: 42 patients planned and analyzed	

Diagnosis and main criteria for inclusion:

Inclusion Criteria:

- Patients with clinical signs and symptoms of VL and diagnosis confirmed by visualization of parasites in tissue samples (lymph node or bone marrow) with microscopy.
- Patients aged between 4 and 60 years (inclusive) who were able to comply with the protocol. It was justified to include children because they represented more than 50% of VL cases.
- Patients for whom written informed consent had been signed by the patients themselves (if aged 18 years and over) or by parents(s) or legal guardian for patients under 18 years of age.

Exclusion Criteria:

- Patients who had received any anti-leishmanial drug in the last 6 months.
- Patients with a negative lymph node / bone marrow smears.
- Patients with a clinical contraindication to lymph node/ bone marrow aspirates.

- Patients with severe protein and or caloric malnutrition (Kwashiokor or marasmus)
- Patients with previous hypersensitivity reaction to sodium stibogluconate or aminoglycosides.
- Patients suffering from a concomitant severe infection such as tuberculosis or any other serious underlying disease (cardiac, renal, hepatic), which would preclude evaluation of the patients response to study medication.
- Patients suffering from other conditions associated with splenomegaly such as schistosomiasis
- Patients with previous history of cardiac arrhythmia or an abnormal electrocardiogram
- Patients who were pregnant or lactating
- Patients with abnormal clinical laboratory values (i.e. haemoglobin < 5 gm/dl, white blood cell count <1 x $10^3/mm^3$, platelets <40,000/mm^3, liver function tests more than three times the normal range, serum creatinine outside the normal range for age and gender)
- Patients with pre-existing clinical hearing loss
- Patients who were human immunodeficiency virus-positive

Test product, dose and mode of administration, batch number:

20 or 15 mg/kg/day of PM, administered by intramuscular injection

Batch number FB 301X

Duration of treatment: 20 mg/kg/day for 21 days and 15 mg/kg/day for 28 days

Reference therapy, dose and mode of administration, batch number: None

**Name of Sponsor/Company**: Drugs for Neglected Diseases initiative (DND*i*), Geneva, Switzerland	Individual Study Table<br>Referring to Part of the Dossier	(For National Authority Use Only)
**Name of Finished Product**: Paromomycin		Volume:
**Name of Active Ingredient**: Paromomycin sulphate		Page:

Criteria for evaluation:

Efficacy: The primary efficacy endpoint was parasite clearance from bone marrow aspirates at days 22 and 29 (for the 20 mg/kg and 15 mg/kg groups, respectively) following initiation of treatment (i.e., parasite clearance at EOT). The secondary efficacy endpoints were parasite clearance from bone marrow aspirates at 3 and 6 months follow-up, post EOT.

Safety: Safety was evaluated by monitoring the occurrence of any adverse events (AEs) and the occurrence of any abnormal clinical laboratory, vital sign, or other physical measurements during the study and follow-up periods.

Statistical methods:
The treatment efficacy in each arm was the percentage of patients in whom the treatment was a success. All analyses were performed in the intent-to-treat population.

Missing data were handled in three ways:

1. Complete case analysis: excluding patients with missing data from estimation of efficacy in each arm
2. Worst case analysis: patients with missing efficacy data were allocated efficacy results based on their ‘worst-case’ scenario i.e. treatment failure.
3. Last parasitology carried forward: Patients with missing efficacy data had their last parasitology results carried forward

Where appropriate, binary data were compared between groups using chi-squared or Fisher’s exact test and continuous or discrete data using t-tests, ANOVA or non-parametric tests.

SUMMARY - CONCLUSIONS

EFFICACY RESULTS: Daily dosing with either 20 mg/kg of PM for 21 days or 15 mg/kg of PM for 28 days resulted in parasite clearance in 85% and 90% of VL patients, respectively at EOT. Two patients in each group relapsed by 6 months resulting in slight reduction in efficacy (76.2% and 81.0% with 20 mg/kg and 15 mg/kg treatment, respectively) at this follow-up (secondary endpoint). This was an improvement compared with the <50% efficacy among all patients in Sudan treated with 15 mg/kg/day for 21 days for whom data was available 6 months after the initiation of the study (LEAP 0104 Part I results).

Thus the primary objective of identifying a more efficacious dose of PM was met. Baseline characteristics of patients in both groups were similar. However, the two doses cannot be compared directly.

SAFETY RESULTS: There were no unexpected safety issues that arose during this study. There were no deaths or other serious AEs. The most common AE in both treatment groups was pain at the site of injection related to the administration of treatment. Audiometric shift at the EOT assessment was detected in 3 patients per group. In one patient in the 20 mg/kg arm, the shift was considered to be disabling hearing impairment. Although not a complete return to normal at 6 months, the disabling hearing impairment at EOT was no longer considered to be disabling. Other shifts returned to normal.

CONCLUSION:

Both doses of PM delivered to 21 patients in each group at total doses higher than in LEAP 0104 Part I were more efficacious. Both doses were well-tolerated and treatment-limiting AEs were not identified. Ototoxicity was reported in six patients from both groups at EOT. This resolved in five patients at the 6-month follow-up, and improved in one (2.4% of treated patients) patient.

Date of the report: 17th December 2009

TABLE OF CONTENTS

2 SYNOPSIS 2
3 LIST OF ABBREVIATIONS AND DEFINITION OF TERMS 12
4 ETHICS 14
4.1 INDEPENDENT ETHICS COMMITTEE (IEC) 14
4.2 ETHICAL CONDUCT OF THE STUDY 14
4.3 PATIENT INFORMATION AND CONSENT 14
5 INVESTIGATORS AND STUDY ADMINISTRATIVE STRUCTURE 15
6 INTRODUCTION 16
6.1 Background of the disease 16
6.2 Rationale for the study and treatment choice 17
7 STUDY OBJECTIVES 18
8 INVESTIGATIONAL PLAN 18
8.1 OVERALL STUDY DESIGN AND PLAN - DESCRIPTION 18
8.2 DISCUSSION OF STUDY DESIGN, INCLUDING THE CHOICE OF CONTROL GROUPS 19
8.2.1 Efficacy 19
8.2.2 Pharmacokinetics 20
8.3 SELECTION OF STUDY POPULATION 20
8.3.1 Inclusion Criteria 21
8.3.2 Exclusion Criteria 21
8.3.3 Removal of Patients from Therapy or Assessment 22
8.4 TREATMENTS 22
8.4.1 Treatments Administered 22
8.4.2 Identity of Investigational Product(s) 22
8.4.3 Method of Assigning Patients to Treatment arms 22
8.4.4 Selection of Doses in the Study 23
8.4.5 Selection and Timing of Dose for each Patient 23
8.4.6 Blinding 23
8.4.7 Prior and Concomitant Therapy 23
8.4.8 Treatment Compliance 23
8.5 EFFICACY AND SAFETY VARIABLES 24
8.5.1 Efficacy and Safety Measurements Assessed and Flow Chart 24
8.5.1.1 Parasitology *.......... 24*
8.5.1.2 Audiometry *.......... 24*
8.5.1.3 Electrocardiographs *.......... 25*
8.5.1.4 HIV testing *.......... 25*
8.5.1.5 Adverse Events *.......... 26*
8.5.2 Appropriateness of Measurements 26
8.5.3 Primary Efficacy Variable 26
8.5.4 Drug Concentration Measurements 27

8.6 DATA QUALITY ASSURANCE 27
8.7 STATISTICAL METHODS PLANNED IN THE PROTOCOL AND DETERMINATION OF SAMPLE SIZE 27
8.7.1 Statistical and Analytical Plans 27
8.7.1.1 Primary efficacy analysis *28*
8.7.1.2 Secondary efficacy analysis *28*
8.7.1.3 Slow Response to Treatment *29*
8.7.1.4 Baseline Data *29*
8.7.1.5 Timing of Follow-up Data Collection *29*
8.7.1.6 Parasitological Efficacy *29*
8.7.1.7 Biological Markers, ECG, Audiometry and Urinalysis *29*
8.7.1.8 Serious and Non-serious Adverse Events *30*
8.7.2 Determination of Sample Size 31
8.8 CHANGES IN THE STUDY CONDUCT OR PLANNED ANALYSES 31
9 STUDY PATIENTS 31
9.1 Disposition of Patients 31
9.2 Protocol Deviation 32
10 EFFICACY EVALUATION 32
10.1 Data Sets Analysed 32
10.2 Demographic and Other Baseline Characteristics 32
10.3 Measurements of Treatment Compliance 36
10.4 Efficacy Results and Tabulations of Individual Patient Data 36
10.4.1 Analysis of Efficacy 36
10.4.1.1 Primary Efficacy Endpoint *36*
10.4.1.2 Secondary Efficacy Endpoint *36*
10.4.2 Statistical/Analytical Issues 38
10.4.2.1 Adjustment for Covariates *38*
10.4.2.2 Handling of Dropouts or Missing Data *38*
10.4.2.3 Interim Analyses and Data Monitoring *38*
10.4.3 Tabulations of Individual Response Data 38
10.4.4 Drug dose, Drug Concentration, and Relationships to Dose 38
10.4.5 Drug-Drug and Drug-Disease Interaction 39
10.4.6 Efficacy Conclusion 40
11 SAFETY EVALUATION 40
11.1 Extent of Exposure 40
11.2 Adverse Events 41
11.2.1 Brief Summary of Adverse Events 41
11.2.2 Display of AEs 42
11.2.3 Analysis of AEs 42
11.3 Deaths, Other Serious Adverse Events, and Other Significant AEs 42
11.3.1 Listings of deaths, other serious adverse events, and other significant

AEs ... 42
11.3.2 Narratives of deaths, other serious adverse events, and other significant adverse events ... 43
11.3.3 Analysis and discussion of deaths, other SAEs, and other significant AEs. ... 43
11.4 Clinical Laboratory Evaluation ... 43
11.4.1 Evaluation of Laboratory Parameters ... 43
11.5 Vital Signs, Physical Findings, and other Observations Related to Safety ... 45
11.6 Safety Conclusions ... 46
12 Discussion and Overall Conclusions ... 47
13 Tables, Figures, and Graphs referred to but not included in the Text 48
14 Reference List ... 48
15 Appendices ... 50

LIST OF TABLES

Table 2-1	Complete Parasite Clearance (Test of Cure) by site and treatment arm (LEAP 0104 Part I) at End of Treatment	2
Table 8-1	Evaluation schedule for this study	24
Table 10-1	Demographic Characteristic of Patients	33
Table 10-2	Baseline Vital Sign and Other Physical and Clinical Measurements	34
Table 10-3	Baseline Laboratory Values	35
Table 10-4	Timing of the 6-month follow-up, n (%)	36
Table 10-5	End of Treatment Analysis (Complete Case Analysis)	36
Table 10-6	3-Month Secondary Efficacy Endpoint	37
Table 10-7	6-Month Secondary Efficacy Endpoint: Complete Case Analysis	37
Table 10-8	6-Month Secondary Efficacy Endpoint: Last Parasitology Carried Forward	37
Table 11-1	Paromomycin Daily Dose per Day	40
Table 11-2	Total Paromomycin Dose Received During the Study	40
Table 11-3	Number of Adverse Events (AEs) and Number of Patients Experiencing AEs During the Study	41
Table 11-4	All Non-serious Adverse Events by Treatment and Relation to Drug, n (%)	42
Table 11-5	Clinical Laboratory Values at End of Treatment	44
Table 11-6	Comparison of Urinalysis Outcome between Baseline and End of Treatment, n (%)	45
Table 11-7	Vital signs and Other Physical Measurements at End of Treatment	46
Table 11-8	Audiometric Results* During Treatment and Follow-up	46

LIST OF FIGURES

Figure 9-1	Patient flow in LEAP 0104 Part II	32

3 LIST OF ABBREVIATIONS AND DEFINITION OF TERMS

AAU	Addis Ababa University
AE	Adverse event
ADR	Adverse drug reaction
AIDS	Acquired immunodeficiency syndrome
ALT	Alanine aminotransferase
ANCOVA	Analysis of covariance
ANOVA	Analysis of variance
AP	Alkaline phosphatase
AST	Aspartate aminotransferase
BMI	Body mass index
BUN	Blood urea nitrogen
CBC	Complete blood count
CCR	Centre for Clinical Research
CRF	Case report form
DC	Definitive cure
DNDi	Drugs for Neglected Diseases Initiative
DMC	Data management center
DSMB	Data safety monitoring board
ECG	Electrocardiogram
EOT	End of treatment
GCP	Good clinical practice
Hb	Haemoglobin
HIV	Human immunodeficiency virus
ICH	International Conferences on Harmonization
IEC	International Ethics Committee
IEND	Institute of Endemic Diseases
i.m.	Intramuscular
IQR	Interquartile range
KEMRI	Kenya Medical Research Institute
LEAP	Leishmaniasis East Africa Platform
LPCF	Last parasitology carried forward
MedDRA	Medical dictionary for Regulatory Activities
MSF	Médicins Sans Frontières
N	number
NR	Not related
PI	Principal investigator
PKDL	Post kala-azar dermal leishmaniasis
PM	Paromomycin sulphate
RBC	Red blood cell count
SAC	Scientific Advisory Committee
SAE	Serious adverse event

SD	Standard deviation
SOC	System organ class
SSG	Sodium stibogluconate
TEAE	Treatment emergent adverse events
UNICEF	United Nations Children's Fund
VCT	Voluntary counseling and testing
VL	Visceral leishmaniasis
WBC	White blood cell count
WFP	World food program
WHO	World Health Organisation
WHO-TDR	WHO – Special Programme for Research and Training in Tropical Diseases

4 ETHICS

4.1 INDEPENDENT ETHICS COMMITTEE (IEC)

The Principal Investigator, Dr Ahmed Musa Mudawi, submitted the protocol amendment #5 dated 8th July 2005 of LEAP 0104 protocol dated 31st July 2004 for approval to the Ethics Committee of the Institute of Endemic Diseases, University of Khartoum, and the Department of Communicable Diseases, Federal Ministry of Health. Approval to proceed was received on 4th August 2005. The full text of the amendment can be found in Section 15.1.1.

Patients (adults with body weight >30 kg) who consented to participate in the pharmacokinetics (PK) evaluation signed an additional/adapted consent form - see Section 15.1.3.

4.2 ETHICAL CONDUCT OF THE STUDY

The trial was conducted in accordance with the Declaration of Helsinki (2002 version) relating to the conduct of research on human subjects and followed the International Committee for Harmonisation (ICH) guidelines for the conduct of clinical trials. All trial site personnel received relevant training in Good Clinical Practice (GCP). The WHO – Special Program for Research and Training in Tropical Diseases (WHO-TDR) handbooks were used for training and for reference before and during the trial.

The Data Safety Monitoring Board (DSMB) appointed at trial start for LEAP 0104 in late 2004 also oversaw this dose-ranging sub-study. See LEAP 0104 CTR Part I for further details of DSMB.

The trial was regularly monitored by GCP trained monitors, initially by Dr Shibru Berhanu, from Ethiopia and subsequently by Dr Robert Balikuddembe from Uganda and Mr Dedan Kinoti from Kenya. During monitoring visits, data entered into the trial case report forms (CRFs) were checked against source data e.g. laboratory log books, analyser printouts, nursing records etc., for verification.

4.3 PATIENT INFORMATION AND CONSENT

Prospective trial participants were drawn from communities well known to the partner institution (Institute of Endemic Diseases, Khartoum) and on the basis of prior knowledge that visceral leishmaniasis (VL) was a major health hazard for those communities. The Kassab site is situated within the major VL endemic region of Sudan; Gedaref State where it is estimated that the majority of cases occur. The affected communities had taken part in previous research and/or drug trials and therefore the level of prior knowledge of VL, its diagnosis, treatment and possible outcomes was quite high.

The Principal/site Investigators (PI) and trial field workers met local community leaders and other local health care personnel before and during recruitment, these people together with patient family members and neighbours also provided a valuable informal resource for locating patients for follow up assessment. The level of community commitment to assist the trial was high due to the past positive experiences these communities had had with the staff of the partner institution.

The patient information and consent procedure was conducted as a process over a period of time during which diagnosis and pre-trial assessments for suitability were performed. Patient information sheets and consent forms were translated into Arabic.

Prospective patients/trial subjects with clinical signs and symptoms suggestive of VL were identified by active case-finding process during field trips to villages in the endemic areas or when patients presented spontaneously at the hospital trial site.

At the point of presentation, the trial and its purpose was described to prospective participants/parents/guardians by the trial site staff. Prospective patients were identified by a combination of evaluation of clinical history, signs and symptoms and leishmania skin tests. If they were willing, prospective patients were diagnosed by identifying parasites in tissue aspirates. The majority of patients presented directly to the hospital trial site as it has become more widely known that treatment was available there.

Patients in whom no positive diagnosis of VL could be made were investigated for other conditions by the trial site teams and treated or referred onwards to Gedaref hospital as appropriate. Where clinical suspicion for VL remained high, but parasitology was initially negative, patients were kept as in-patients at the hospital trial site and re-investigated a week later.

Patients who had positive parasitological assessment and were otherwise eligible, gave written or documented witnessed verbal consent to participate after the completion of the pre-trial diagnostic tests at the hospital trial site. Consent was obtained from parents or guardians for all minors.

Once patients had given consent, they were randomised to trial treatment by means of sequentially numbered, sealed envelopes containing the treatment allocation (generated by the Centre for Clinical Research (CCR) Data Centre, Kenya Medical Research Institute (KEMRI) and the first dose administered.

All patients who had a positive diagnoses for VL at the trial site, i.e. parasites identified by microscopy of fresh tissue samples taken from the patient, received treatment as in-patients from the trial site teams on the designated trial wards, either within the trial if they were eligible and consented to trial participation, or otherwise outside the trial.

All patients with a positive diagnosis of VL, whether or not they participated in the trial, were treated at the hospital trial site and additionally provided with food and all other needed medication and investigations free of charge.

All patients who did not respond to trial treatment, who deteriorated clinically during treatment, or were withdrawn from trial treatment for other reasons, were given rescue treatment with Ambisome® (liposomal amphotericin B).

5 INVESTIGATORS AND STUDY ADMINISTRATIVE STRUCTURE

This dose-ranging protocol amendment #5 was agreed and designed by the Leishmaniasis East Africa Platform (LEAP) (see LEAP 0104 Clinical Study Report part I for details).

The trial was coordinated from the DND*i* Africa office at CCR, KEMRI, Nairobi where the trial Medical Coordinator, trial manager, trial data centre and the Trial Master File were also located. The trial monitors, finance and administration were initially managed by *DNDi* Geneva and later delegated to the DND*i* Africa office.

Progress was evaluated monthly by the DND*i* Research and Development Director and Executive in Geneva, on the basis of a written report from the project manager based on a collation of reports from the trial sites, monitors, trial manager, and medical coordinator.

Serious Adverse Events (SAEs) were to be reported to the trial coordination office at CCR KEMRI Nairobi from trial sites, by phone, fax or e-mail for discussion, decision-making and onward reporting to national authorities. Additionally, all SAEs were reported as they occurred to the Chair of the DSMB for onwards transmission and discussion by the DSMB.

All clinical trial supplies, both drugs and equipment, were obtained via DNDi's project procurement partner the International Dispensary, Amsterdam, Netherlands, who was also the sole supplier and distributor for PM, as in part I of the trial.

A list of investigators, their roles and their qualifications is given in Section 15.1.4.

The PK analyses were carried out at the laboratory of Professor Gilbert Kokwaro at KEMRI Nairobi.

Dr Tony Moody assisted the trial site with parasitology training, slide reading and general laboratory management. Dr Moody also performed a second independent 'blind' reading (without knowledge of trial treatment allocation) of all trial slides to ensure quality and accuracy of reporting by trial site laboratory technicians as for part I.

6 INTRODUCTION

6.1 Background of the disease

Leishmaniases are a group of diseases caused by Leishmania parasites, of which at least 20 different species can cause human disease. Leishmania infection is transmitted to humans by the bite of a female sandfly. The disease occurs in four forms:

- self-healing cutaneous leishmaniasis,
- mutilating mucosal leishmaniasis,
- VL (in India also known as kala azar) the most common form in Eastern Africa, and
- post kala-azar dermal leishmaniasis (PKDL), which occurs in recovering patients months to years after the primary infection, and is prevalent in Sudan but much less commonly seen elsewhere.

Not all infected people develop clinical symptoms; some have a subclinical infection which resolves spontaneously. Patients with symptomatic VL present with fever, malaise, cough, abdominal pain, diarrhoea, epistaxis, splenomegaly, hepatomegaly, cachexia, anaemia, pancytopenia, lymhadenopathy, and malnutrition.

The incubation period for VL varies widely, and is estimated to be between 2 – 6 months. Malnutrition, anaemia and immune depression increase the likelihood that infection will progress to the clinical, life-threatening disease.

Untreated, symptomatic VL is fatal. Children living in endemic areas who have not been previously exposed and therefore have no immunity are particularly susceptible. Sub-clinical infections that may occur in semi-immune patients, for example previously exposed adults in endemic areas, and who do not present for treatment may be reservoirs of infection in the community. Recently, it has become evident that VL is also

an opportunistic infection that can affect immuno-compromised patients such as those with human immunodeficiency virus (HIV) infection and it is now accepted as an acquired immunodeficiency syndrome (AIDS)-defining co-infection (WHO 2007).

The high prevalence of PKDL (in up to 50% of recovering patients) seems to be a particular feature associated with the disease variant in Sudan (Zijlstra, Musa et al. 2003). In these cases, the skin is heavily infected with parasites although the person is systemically well, and these can be an additional reservoir of infection.

The WHO estimates that worldwide about 500,000 new cases of VL occur per year, but due to the remote location of communities in the VL endemic areas most are untreated. Ninety percent of VL cases occur in five countries: India (especially Bihar), Bangladesh, Nepal, Sudan and north eastern Brazil (Desjeux 1996).

In Eastern Africa, especially in Sudan, Ethiopia, and Kenya, VL is associated with high mortality and morbidity, exacerbated by poor nutritional status, and in some areas it is the commonest health hazard that communities face. However, the disease presentation and patient demographics vary from area to area, and even within countries.

In Sudan, Gedaref State, Upper Nile and Western-Upper Nile are known endemic areas for VL with more than 51,000 cases (of which were 3,900 deaths) treated by Médicins Sans Frontiers (MSF) alone from 1989 to 2003 (Ritmeijer and Davidson 2003). The majority of cases were children and HIV co-infection was at less than 1%.

Population displacements as a result of war, drought, famine and rural-urban migration have exacerbated the spread of the disease, when non-immune populations move into VL endemic areas. For instance, the epidemic in western Upper Nile in Sudan, an area where VL was previously not endemic, caused approximately 100,000 deaths between 1984 and 1992, a population mortality of up to 36% (Seaman, Mercer et al. 1996).

6.2 Rationale for the study and treatment choice

Current standard treatment for VL in East Africa is sodium stibogluconate (SSG). However, SSG has several drawbacks including: cardiac toxicity, involves a 4-week treatment requiring hospitalization (therefore increasing the economic burden on patients), and potential for development of resistance - in endemic areas in India the parasite is already resistant to SSG, making it unusable.

Therefore there is a need for alternative treatments in Africa. Two phase II studies in India showed that PM given at a dose of approximately 15 mg/kg/day for 21 days was efficacious, with definitive cure rates of 93% (Jha et al 1998) and 89% (Thakur et al 2000). Both studies also showed that PM given at a dose of 20 mg/kg/day for 21 days was both safe and efficacious (cure rates of 97% and 86% in the Jha et al 1998 and Thakur et al 2000, studies, respectively). Additionally, MSF has gained valuable field experience with the drug in an emergency situation in Sudan (Seaman, Pryce et al. 1993).

The current study is part of a larger study aimed at evaluating the safety and efficacy of PM for VL treatment in East Africa. In Part I of LEAP 0104, PM delivered at a dose of 15 mg/kg/day for 21 days was not effective in patients in Sudan. To determine an effective dose for the planned larger trial (LEAP 0104 Part III), the trial was amended to include the current intermediate dose-finding study.

In the current study, two treatment regimens, 20 mg/kg/day PM for 21 days and 15 mg/kg/day for 28 days, were evaluated for parasitiological test of cure (TOC) 22 or 29

days following initiation of treatment, respectively for the two groups.

There was no previous clinical experience with the 15 mg/kg dosage given for 28 days. The rationale for this treatment arm was that the longer course of treatment would provide additional time for the patient's general condition to improve, and for their immunological response to develop, and that this might translate into a better clinical response without increasing the daily dosage. There was ample prior clinical trial evidence that PM at a dose of 15 mg/kg/day was well tolerated (personal communication at the time of amendment #5 design, and subsequently published, Sundar et al 2007).

7 STUDY OBJECTIVES

The primary objective of this study was to evaluate efficacy of PM dosing regimens (20 mg/kg/day for 21 days and 15 mg/kg/day for 28 days) for the treatment of VL in Sudan.

The secondary objectives were to evaluate safety and perform PK studies in a subset of adult VL patients to further elucidate the possible causes of failure of the PM 15 mg/kg/day for 21 days regimen in Sudan in LEAP 0104 part I.

8 INVESTIGATIONAL PLAN

8.1 OVERALL STUDY DESIGN AND PLAN - DESCRIPTION

This was a single site, open study documented as a protocol amendment to LEAP 0104 (LEAP 0104 Amendment 5 dated 8th July 2005, Section 15.1.1). The study centre was the Ministry of Health Hospital Kassab, Gedaref State, Sudan. The goal was to further elucidate the efficacy and safety of two high dosing regimens of PM monotherapy, and perform PK analysis on a subset of patients to address the lack of efficacy issue in Sudan that had been identified in Part I of LEAP 0104 trial.

The two study arms were: PM 20 mg/kg/day for 21 days (n = 21) and PM 15 mg/kg/day for 28 days (n = 21). There was no placebo arm as acute VL is a potentially rapidly fatal disease. AmBisome was used as rescue treatment as in LEAP 0104 Part I.

Forty-two patients that fulfilled the inclusion criteria and had none of the exclusion criteria were selected from the patient population at the study site and enrolled in the study. Due to the differing lengths of the treatment regimens in each arm, this was an open study.

Restricted block randomization of size 6 was performed for the two arms. Patients were randomly allocated to the treatment arms using sequentially numbered sealed envelopes that were prepared according to a centrally generated randomisation list from the data management centre (DMC) at KEMRI Nairobi. Thus the investigator or patient did not know treatment allocation for any particular patient until the envelope was opened at the initiation of treatment.

Patients received the first dose of their allocated treatment right after allocation, and continued to receive daily administration of the assigned drug as hospital in-patients for the duration of treatment. They were followed up at 3 and 6 months post-treatment as out-patients. The final assessment was performed at 6 months post-treatment.

The primary efficacy endpoint, was TOC (parasite clearance) at EOT (i.e. on day 22 for the 20 mg/kg group and day 29 for the 15 mg/kg group). Due to the long distances between patients' villages and the trial sites, patients often stayed at the hospital trial site for a few days to complete the outpatient follow-up assessments at 3 months (at the discretion of the investigator) and 6 months post-treatment. The final assessment

included parasitology to assess definitive cure (DC). If patients did not attend a follow-up visit, health workers visited the patient's village to try to establish if the patient was alive and well, had died, or had moved away.

All patients with a positive diagnosis of VL whether or not they participated in the trial, were treated and additionally provided with food and all other needed medication and investigations, free of charge.

Patients who did not respond to trial treatment or who clinically deteriorated during treatment with the study medication received rescue medication (AmBisome), where required.

For the pharmacokinetic analysis, the first six patients with body weight of 30 kg or more from each treatment arm had additional venous blood and urine sampling (Table 8-1).

No interim analysis was planned or carried out in LEAP 0104 Part II.

8.2 DISCUSSION OF STUDY DESIGN, INCLUDING THE CHOICE OF CONTROL GROUPS

The design of this dose-finding study was based on results from Part I of the LEAP 0104 study (which showed the inefficacity of a 15 mg/kg/day treatment for 21 days in Sudan) and results from previous clinical trials performed with PM in India that showed the efficacy of treatment with 20 mg/kg/day for 21 days (see Rationale Section 6.2).

In the current study we evaluated two regimens with higher total dose of PM than that administered in Part I of LEAP 0104. Increased total dose was achieved either by increasing treatment duration of the 15 mg/kg/day dose to 28 days or increasing the daily dose to 20 mg/kg/day but maintaining treatment duration (21 days).

8.2.1 Efficacy

Efficacy analysis was based on parasite clearance in bone marrow aspirates. Splenic aspiration for parasitological assessment of VL is not allowed in rural health centres that lack surgical facilities in Sudan.

The primary efficacy endpoint in the current study was parasite clearance at EOT (i.e. 22 and 29 days following initiation of treatment for the 20 mg/kg and 15 mg/kg groups, respectively). Although the primary efficacy endpoint is normally DC at 6 months, in the current study there were concerns of increased toxicity during treatment due to a higher total dose and the theoretical risk that patients could be lost to follow-up at 6 months, which would compromise the study due to the low number of patients enrolled. Previous studies have shown that parasite clearance at 30 days is good indicator of efficacy, though not definitive. This allowed us to proceed to Part III of the LEAP 0104 study since PM was an unlicensed product and it was not known at trial start if it would be possible to follow up all patients after discharge from hospital. The secondary efficacy endpoint was parasite clearance at the 3- (optional) and 6-month follow-up. There were no control groups in this study.

Patients who were clinically well and parasite negative at EOT were discharged and reviewed at 3 months if the investigator deemed it necessary. Patients who were clinically well but had parasites at a lower level than at trial entry i.e. slow responders, could be discharged but were seen earlier than planned in the protocol, i.e. at 1 month or sooner if symptoms recurred. Patients who were still parasite positive at the 6-month follow-up were given rescue treatment with AmBisome. The decision when to give rescue treatment, based on the overall clinical picture of symptoms, signs and laboratory

parameters was the responsibility of the trial physician.

It is generally acknowledged that response to drug treatment in VL is not only a result of the parasitocidal effect of the drug, but also dependent on the nutritional and the immunological status of the individual patient at the time of infection. Patients, particularly adults living in endemic areas, might be partially immune due to previous exposure to the leishmania parasite, i.e. previous sub-clinical infection, whereas other patients, e.g. young children with their first exposure to the parasite, or adults moving to an endemic/transmission area might be immunologically naïve. Concurrent or recent other infections, for example malaria or pneumonia, might also have a deleterious effect on response by suppressing immunological response.

The trial sites provided daily food (3 meals) for all trial patients free of charge whilst in hospital to ensure adequate nutrition during treatment. In addition, children were given supplements of the proprietary product 'plumpy nut' from hospital supplies provided by United Nations International Children Fund (UNICEF)/World Food Program/MSF whenever these were available at the discretion of the trial physician/nurse. Severely malnourished children and adults were not eligible for the trial and were treated with SSG at the trial sites, by trial site staff but outside the trial.

None of the above factors could be completely controlled for, and it is clear that many patients were malnourished at trial entry and many had a history of recent infection, particularly malaria or respiratory tract infection that may have altered their immunological status. These factors were discussed during the planning of the trial and the consensus was that it was important to mimic, as much as possible, the usual clinical situation.

In summary, the trial patient population was heterogenous with regard to their general health status, but accurately reflected the diversity of clinical presentations and the 'real life' situation under which potential new treatments would be tested in phase III trials.

8.2.2 Pharmacokinetics

The first six patients in each treatment arm, with a body weight ≥30 kg, and who gave consent to participate in the PK part of the study were to undergo additional venous blood and urine sampling for pharmacokinetic evaluation as outlined in Appendix 1 of the protocol amendment #5 (Section 15.1.1).

At the time of the study design, there were no reports of PK studies with PM in VL patients. Although PK had been included in the Indian phase III trial this had not yet been reported by the beginning of this study, therefore there was no prior knowledge of PK of PM in VL patients. PK results were only known for studies in healthy volunteers (Kanyok et al 1997).

8.3 SELECTION OF STUDY POPULATION

Inclusion and exclusion criteria were unchanged from those in the original protocol except that HIV-positive patients were excluded from participation in the protocol amendment to limit further confounding factors that might affect efficacy, safety, or PK evaluation.

Potentially eligible patients with clinical signs and symptoms suggestive of VL were identified when they presented spontaneously at the hospital trial sites. At the point of presentation, the trial and its purpose was described to prospective trial participants/parents/guardians in their local language by field workers or the trial site

staff. It was justified to include children because they represent more than 50% of VL cases, however for the PK study only patients with body weight >30 kg were included.

In the field, prospective patients were identified by a combination of clinical history, signs and symptoms, supplemented by specific field tests for VL, for example direct leishmanin skin tests. After initial assessment patients were further investigated and a definitive diagnosis made by identifying parasites in tissue aspirates. Only when all these tests had been performed and reviewed by the trial physician was the patient/guardian asked to sign the consent form and the patient randomised and started on trial treatment. Patients, in whom no positive diagnosis of VL could be made on the basis of examination of fresh tissue aspirates, were investigated for other conditions by the trial site teams and treated, or referred onwards as appropriate. For cases where clinical suspicion for VL remained high, patients were kept as inpatients and reinvestigated a week later.

All patients who were positively diagnosed with VL, i.e. parasites were identified by microscopy of fresh tissue (lymph node or bone marrow) samples taken from the patient, received treatment as inpatients from the trial site teams on the designated trial wards, either within the trial if they were eligible and consented, or outside the trial.

8.3.1 Inclusion Criteria

1. Patients for whom written informed consent had been signed by the patients themselves (if aged 18 years and over) or by parents(s) or legal guardian for patients under 18 years of age.
2. Patients aged between 4 and 60 years (inclusive) who were able to comply with the protocol. It was justified to include children because they represented more than 50% of VL cases.
3. Patients with clinical signs and symptoms of VL and diagnosis confirmed by visualization of parasites in tissue samples (lymph node or bone marrow) with microscopy.

8.3.2 Exclusion Criteria

1. Patients who had received any anti-leishmanial drug in the last 6 months.
2. Patients with a negative lymph node / bone marrow smears.
3. Patients with a clinical contraindication to lymph node/ bone marrow aspirates.
4. Patients with severe protein and or caloric malnutrition (Kwashiokor or marasmus)
5. Patients with previous hypersensitivity reaction to SSG or aminoglycosides.
6. Patients suffering from a concomitant severe infection such as tuberculosis or any other serious underlying disease (cardiac, renal, hepatic), which would preclude evaluation of the patients response to study medication.
7. Patients suffering from other conditions associated with splenomegaly such as schistosomiasis
8. Patients with previous history of cardiac arrhythmia or an abnormal electrocardiogram (ECG)
9. Patients who were pregnant or lactating

10. Patients with haemoglobin < 5 gm/dl
11. Patients with white blood cell (WBC) < 1 x $10^3/mm^3$
12. Patients with platelets < $40,000/mm^3$
13. Patients with liver function tests more than three times the normal range
14. Patients with serum creatinine outside the normal range for age and gender
15. Patients with pre-existing disabling hearing impairment
16. Patients who are HIV-positive

8.3.3 Removal of Patients from Therapy or Assessment

Patients were only removed from therapy or assessment at their own request or because of clinical deterioration, failure to respond, or SAE. In all cases patients were then treated with AmBisome if appropriate, as described below. All these patients were to be followed up for 6 months post treatment in the same way as those remaining in the trial, if possible, or until death. No post-mortems were carried out as this is not the cultural norm in Sudan and no facilities or expertise for this procedure are available.

8.4 TREATMENTS

8.4.1 Treatments Administered

Both treatment arms received PM via intramuscular (i.m.) injection in the gluteal muscle.

Rescue medication

In the event of failure to respond to trial treatment, clinical deterioration during trial treatment or relapse at any time during follow up, rescue treatment consisting of an intravenous infusion of AmBisome® (a liposomal formulation of amphotericin B) reconstituted according to the manufacturer's instructions, at a dosage of 3 mg/kg/day for 10 days, total dose 30 mg/kg. Repeated courses of AmBisome could be given if required.

Patients who received AmBisome at any time during the trial were counted as trial treatment failures, but were followed up wherever possible until they responded or died.

Crossover from one trial treatment to another was not allowed as this would have confounded the efficacy evaluation.

8.4.2 Identity of Investigational Product(s)

PM (Batch No. FB 301X) with a shelf life of 24 months was used. The presentation was as a single use, amber glass ampoule containing 1g of paromomycin sulphate in 2 ml.

Trial supplies for each arm of the trial were packed as a complete 'per patient' course in customised boxes containing the correct number of daily doses to assist ease of drug accounting by the trial monitors.

8.4.3 Method of Assigning Patients to Treatment arms

Allocation to trial treatment was by means of sequentially numbered, sealed envelopes, generated from a computerised, randomisation list. The trial site received a box of uniquely numbered sealed envelopes for this part of the trial from the trial DMC, CCR, KEMRI Nairobi where centralised randomisation and envelope preparation was carried

out. The randomisation master list was filed in the Trial Master File in Nairobi.

Once a patient had been confirmed as eligible for the trial and had given consent, the investigator took the next lowest numbered envelope from the box, read the treatment allocation from the card inside and prescribed this treatment for the patient. The opened envelope and card were retained for review and checking by the monitor at each visit.

No PM was used outside the trial to treat any patient, as it is currently unlicensed in Sudan.

8.4.4 Selection of Doses in the Study

The two dosing regimens, 20 mg/kg/day for 21 days and 15 mg/kg/day PM for 28 days were selected based on previous experience (see Rationale in Section 6.2)

8.4.5 Selection and Timing of Dose for each Patient

Except for those patients in the PK evaluation, exact timing of patient dosing was not critical, but generally treatment was given by the clinician or research nurse at approximately the same time each day, usually in the morning, and documented on a treatment sheet indicating time of dosing.

8.4.6 Blinding

Due to the differing treatment durations no medication blinding was considered possible, or attempted.

8.4.7 Prior and Concomitant Therapy

No prior (within 6 months) or concomitant other treatments for VL were allowed. Patients who were diagnosed with malaria during investigation before trial entry were treated for malaria first and then reassessed after one week for trial eligibility. Concomitant respiratory and other bacterial infections were quite common and treatment with the relevant antibiotics was allowed and documented in the patient CRF.

8.4.8 Treatment Compliance

Treatment was given by the clinician or research nurse each day and documented on a treatment sheet indicating time of dosing bearing the signature of the attending clinician/nurse. These sheets were used for drug accountability checks and were reviewed by the trial monitors. The presentation of PM was in single-use, 2-ml glass vials with a snap-off glass top. The empty, broken vials were re-inserted into the daily treatment pack and checked by the monitor at each monitoring visit. It was not possible to retain un-used PM as the vials had snap off tops.

Both trial treatments were parenteral and administered as an i.m. injection into the gluteal muscle by the trial personnel. No patients received the wrong medication i.e. medication to which they had not been previously allocated. Drug accountability sheets were kept for each trial patient and checked at monitoring visits. Concomitant medications were recorded in the trial CRF for each patient as was rescue treatment with AmBisome.

Monitors also checked expiry dates of trial medication. After drug accounting procedures had been completed, the used medication packs were destroyed according to the Institute of Endemic Diseases (IEND) standard operating procedures.

8.5 EFFICACY AND SAFETY VARIABLES

8.5.1 Efficacy and Safety Measurements Assessed and Flow Chart

Patients were seen daily throughout the pre-trial diagnostic assessment period and whilst on trial treatment by the trial site physician or deputy and by the trial nurse. Formal assessments as shown in the table below were carried out at baseline and at weekly intervals whilst on treatment and on the day after the EOT.

Table 8-1 Evaluation schedule for this study

Assessment	Day of Treatment				Follow-up	
	0	7 days	14 days	End of Treatment†	3 months	6 months
Efficacy : Parasitology‡	✓			✓	✓	✓
ECG and Audiometry	✓	✓	✓	✓	✓	✓
Biological Markers°	✓	✓	✓	✓	✓	✓

† End of treatment is Day 22 for 20 mg/kg for 21 day regimen, Day 29 for 15 mg/kg for 21 day regimen
‡ Measured at 3 months if patient clinically unwell
°Temperature, Spleen size, Liver Size, Weight, Haemoglobin, Platelets, White cell count, heart Rate, Systolic and Diastolic blood pressure, Bilirubin, BUN, Creatinine, ALT, AST

For patients participating in the PK evaluation, blood and urine sampling were taken on day 1 for both groups and day 14 and 26 for the 20 mg/kg and 15 mg/kg groups, respectively. These samplings were to be carried out in the mornings, but not at any particular time in relation to drug administration. Analysis was carried out by the trial laboratory technician or deputy at the trial site. There was no central laboratory. Parasitology, audiometry, and ECGs were carried out as indicated on the flow chart (Table 8-1). Tests other than the ones in Table 8-1 could be carried out as required to assist the physician in patient management but were not stipulated in the protocol.

8.5.1.1 Parasitology

Lymph node and bone marrow aspirates were performed using standard methods. Parasitology slides were prepared, read and reported according to a standardised, approved WHO method supervised by Dr Tony Moody who trained all trial laboratory technical staff.

8.5.1.2 Audiometry

Audiometry was performed at a convenient quiet time, often in the early morning, to reduce background noise, which may otherwise interfere with the patient's ability to perform the test. A professional audiometrist (Serah Ndegwa, Kenyatta National Hospital, Nairobi, Kenya) trained all trial personnel in the correct audiometry technique. The same audiometer that was used in LEAP 0104 Part I was used in this study.

Prior to testing, the Madsen Voyager audiometer was recalibrated to take account of background noise levels, which were checked using a hand-held noise monitor. If the ambient sound level was above 35 dB audiometry testing was deferred until a quieter time. Before initiating the audiometry test, patients' ears were inspected using a standard clinical otoscope and the patient instructed in the audiometry technique to be used and given a handheld buzzer to press as soon as they heard an audible sound. Patients who had difficulty holding and manipulating the buzzer could raise their hand

instead, as soon as they heard the audible sound. The audiometer headphones were then positioned on their head and testing commenced. Each ear was tested in turn. The tester set the audiometer to start with the right ear at a frequency of 1,000Hz and an intensity of 60 dB for air conduction threshold measurements and the signal presented for one to three seconds. If there was a positive response the intensity was reduced in 10 dB steps until no further response was obtained. The intensity of the tone was then increased in 5 dB steps until a response occurred. This procedure was repeated at the same frequency until two out of three positive responses were obtained to an ascending stimulus, and this was then recorded as the threshold for that frequency. If there was no response to 60 dB, the signal was increased in 20 dB steps until a response was obtained.

The following frequencies were then tested in the following order; 2,000 Hz, 4,000 Hz, 8,000 Hz, 500 Hz and 250 Hz. The whole procedure was then repeated for the left ear.

The stimulus was not presented to the patient in a regular time sequence to prevent a predictable rhythm of response to the signal. If the patient gave several false positive responses i.e., pressed the buzzer repeatedly when no stimulus was being presented by the tester, the patient would be re-instructed and the test repeated or deferred to another occasion. The complete test for both ears took about 30-45 minutes to perform for each patient. Generally, the children had no difficulty complying with instructions and rapidly learned what was required of them.

An audiometric shift was defined in patients for whom there was one of the following:

- an increase in hearing level between baseline and EOT of ≥25 dB at ≥1 threshold frequency
- an increase in hearing level between baseline and EOT of ≥20 dB at ≥2 adjacent threshold frequencies

Disabling hearing impairment was determined as an average of at least 41dB across 500Hz, 1000Hz, 2000Hz and 4000Hz frequencies in the better ear in adults (ages 15 years and above) and at least 31db across 500Hz, 1000Hz, 2000Hz and 4000Hz frequencies in the better ear in children (less than 15 years of age) (World Health Organization, 2004).

Per arm, data are presented to show the number and percentage of patients:

- experiencing an audiometric shift at the EOT
- an audiometric shift remaining at 6 months follow-up
- disabling hearing impairment at the EOT
- disabling hearing impairment remaining at 6 months follow-up

8.5.1.3 Electrocardiographs

ECGs were performed with the patient resting supine on the bed using a portable self-reporting ECG machine. The trial site was supplied with a new ECG machine prior to trial start. The tracing was reviewed by the trial physician to assist in clinical management and any abnormality reported in the patient's trial CRF. ECGs have been retained with patient trial records at the trial site.

8.5.1.4 HIV testing

HIV testing and reporting was carried out by specifically trained personnel who could

undertake the required counselling prior to testing. Patients who tested positive were not eligible for part II of the trial, and after treatment of their VL infection were referred to a specialist in Gedaref for further assessment and treatment in accordance with national HIV protocols.

8.5.1.5 Adverse Events

The study investigator was responsible for adverse event (AE) reporting. AE data were obtained by observing and examining the patients daily, by asking patients if they had suffered any untoward effects since they were last seen and by reviewing laboratory data. AEs were rated for severity and causality by the site investigator and were classified using Medical Dictionary for Regulatory Affairs (MedDRA) version 10.0 at the DMC, KEMRI Nairobi with the assistance of the clinical trial manager and project coordinator.

SAEs were discussed and consensus reached with the trial medical coordinator for severity and causality. The approved national SAE reporting procedure for Sudan was coordinated by the trial office at CCR, KEMRI.

Safety assessments (blood biochemistry, haematology, urinalysis, ECG and audiometry) were performed as in LEAP 0104 Part I, i.e., before treatment, on day 7, 14 for both groups and additionally on day 22 for the 20 mg/kg group and day 29 for the 15 mg/kg group so that direct correlations can be made with the PK data (Table 8-1).

8.5.2 Appropriateness of Measurements

For all safety and efficacy measurements, well-known and validated standard procedures were used.

8.5.3 Primary Efficacy Variable

The primary efficacy endpoint (parasite count in bone marrow aspirates on days 22 and 29) was based on standardised parasitology reading of freshly prepared tissue aspirates taken directly from the patients to the laboratory. Slide fields were examined and counted for parasites under oil emersion 100X magnification for 30 minutes (timed) before being declared negative (absence of parasites on microscopy slide at end of treatment).

No tissue aspirate technique is perfect and accuracy can vary with operator technique and experience. It is generally acknowledged that splenic aspirate is the most sensitive but it is also theoretically the most hazardous method and may be associated with intra-abdominal bleeding which can be severe and, rarely, catastrophic. In areas where lymphadenopathy is a prominent clinical presentation a high level of accuracy can be obtained with this method.

In Sudan, lymph node aspiration is stipulated in the national treatment guideline on the grounds of safety as many patients may be treated in rural health posts far from surgical facilities, and this technique was used at the Kassab trial site. Splenic aspiration for parasitological assessment of VL is not allowed in Sudan in rural hospitals lacking adequate surgical facilities.

After treatment, splenomegaly and lymphadenopathy may have regressed dramatically and therefore bone marrow aspiration can be used as an alternative for follow up evaluation. Bone marrow aspiration is safe, but painful unless adequate local anaesthesia is used.

The secondary endpoints were parasite counts in bone marrow at the 3 and 6 months post-EOT follow-ups. Follow-up at 3 months is optional, dependent on investigator concerns following discharge post treatment and seasonal access to remote communities.

Patients who attended follow up were examined for clinical symptoms of VL and classified as clinically well or clinically unwell. Parasitology, measured by visualization of parasites in tissue samples (lymph node or bone marrow) on microscopy, was performed in patients deemed to be clinically unwell.

8.5.4 Drug Concentration Measurements

Prior to this study the only data available on PK of PM was from a healthy volunteer study conducted in North America (Kanyok et al 1997). PK data from VL patients in the Indian study only became available in late 2006, after the current study was completed.

However, data from a separate, single dose PK study in six healthy human volunteers carried out in 2006 by the University of Khartoum were analysed at the same time in the same laboratory and will also be briefly described in relation to the patient data in the study report (Section 15.1.8).

For PK analysis, venous blood sampling (5 ml for duplicate analysis at each time-point) in each group was to be carried out during two sampling periods; on day 1 of treatment for both groups and then subsequently on day 14 in the 20 mg/kg group and day 26 in the 15 mg/kg group to represent steady state conditions. On each day blood samples were drawn at time 0 (before treatment) and at 0.25, 0.5, 1.0, 2, 4, 6, 8, 12, and 24 hours post-dosing.

Twenty-four hour urine samples were to be collected from patients in both groups on the same day that blood was drawn at 0-2, 2-4, 4-6, 6-8, 8-12, and 12-24 hours post-dosing.

The specific methods for the PK analysis are given in the report (Section 15.1.8).

8.6 DATA QUALITY ASSURANCE

The quality assurance mechanisms put in place included adequate pre-trial GCP training of trial site staff and regular ‘refresher’ training. Regular site monitoring and one to one training and mentoring of clinical and laboratory technology staff was undertaken. See LEAP 0104 CTR Part I for further details.

8.7 STATISTICAL METHODS PLANNED IN THE PROTOCOL AND DETERMINATION OF SAMPLE SIZE

8.7.1 Statistical and Analytical Plans

This was a phase II dose-ranging protocol amendment to evaluate if it was possible to improve on the previous unsatisfactory efficacy results seen in Sudan in Part I of the trial.

Part II was not powered to show statistically significant differences in efficacy between treatment in the 20 mg/kg and 15 mg/kg arms. The goal was to identify an efficacy dose at which PM would be effective. During consultation between DND*i*, the LEAP group and the trial statisticians, it was agreed that two groups of 21 patients each would suffice to establish if it was possible to detect acceptably high efficacy that could be introduced into the main protocol as an amended dosing schedule in the PM monotherapy arm, for further testing in a formal phase III prospective test situation. No interim analysis was

planned and simple descriptive statistics were used to express the results.

8.7.1.1 Primary efficacy analysis

Primary efficacy analysis was based on TOC at day 22 (20 mg/kg group) or 29 (15 mg/kg group) post-treatment initiation. TOC was defined as the absence of parasites by microscopy at EOT.

Treatment failure was defined as receipt of rescue medication during treatment period or initial hospitalization period. The treatment efficacy in each arm was the proportion, or percentage, of patients in whom the treatment was a success.

Missing parasitology data at the end of treatment could have arisen if:

- a patient died prior to the end of the treatment period
- a patient was withdrawn from study treatment
- a parasitology exam should have been done but was not (investigator error)

To take account of missing data, efficacy analyses was carried out at EOT, within an Intention-to Treat framework, in two ways:

1. Complete case analysis: excluding patients with missing data from estimation of efficacy in each arm
2. Worst case analysis: patients with missing efficacy data are allocated efficacy results based on their 'worst-case' scenario i.e. treatment failure.

Analyses assumed that EOT time points were comparable, by definition of the treatment regimens.

8.7.1.2 Secondary efficacy analysis

Secondary efficacy analysis was based on parasitological results at the 3 and 6 month follow-ups.

The number of patients who were seen at 3 months, the number of patients who were clinically well, and the number of relapses following non detection of parasites at the end of treatment were determined.

Treatment success was DC at the 6-month follow-up. DC was defined as the absence of parasites on microscope slides at 6 months, provided no rescue medication was given during treatment or follow up period.

Treatment failure was defined as receipt of rescue medication at any point during the trial or parasite visualization on a microscopy slide at 6 months in patients who did not receive rescue medication during treatment or follow-up period. If a patient did not attend the 6-month follow-up, they were considered to be lost to follow-up for analysis purposes, unless rescue had been previously administered to the patient, in which case the efficacy outcome was assumed to be failure.

The efficacy in each arm was the proportion or percentage of treatment successes. Where 6 months parasitology data was missing, efficacy analyses was carried out within an Intention-to Treat framework in three ways:

1. Complete case analysis: excluding patients with missing efficacy data from estimation of efficacy in each arm
2. Worst case analysis: Patients with missing efficacy data were allocated efficacy

results based on their ‘worst-case’ scenario i.e. treatment failure.

3. Last parasitology carried forward: Patients with missing efficacy data had their last parasitology results carried forward.

8.7.1.3 Slow Response to Treatment

Slow response to treatment was described as the number and percentage of patients per arm in whom parasites were visualised at the end of treatment but not at 6 months post EOT with no administration of rescue medication.

8.7.1.4 Baseline Data

At baseline, age was summarised as a continuous variable and within the categories: paediatric (4 to 14 years) and adult (15 and above).

Nutritional status was classified based on Weight for Height and BMI for Age z-scores if age<6 years and 6-19 years respectively and BMI values used if age>19 years. Patients were classified as;

- Normal if -2SD≤ BMI for Age <+1SD or -2SD≤ Weight for Height <+1SD or 18.5≤ BMI <25.0
- Underweight if -3SD≤ BMI for Age <-2SD or -3SD≤ Weight for Height <-2SD or 16.0≤ BMI <18.5
- Severely underweight if BMI for Age <-3SD or Weight for Height <-3SD or BMI<16.0

Continuous or discrete baseline data were summarised using mean and standard deviation (SD) and also using median and inter-quartile range (IQR). Testing for differences across arms was to be conducted using parametric t-tests and non-parametric Wilcoxon rank sum tests for continuous data. Binary and categorical data were to be summarised using proportions and compared using chi-squared or Fishers test.

8.7.1.5 Timing of Follow-up Data Collection

Where the 3-month follow-up date of a patient fell between 1.5 and 4.5 months post EOT assessment (expected 3 months follow-up date ±45 days), data were treated as 3 months follow-up data. When the date of a patient follow-up fell anywhere beyond 4.5 months post EOT assessment, data were treated as 6 months follow-up data (TOC assessment date plus 135 days or more).

8.7.1.6 Parasitological Efficacy

Efficacy and corresponding 95% confidence intervals (CI) are presented for each PM regimen. The efficacy and 95% CI for the original PM dose of 15 mg/kg for 21 days, as assessed on Day 22 was also presented for historical, visual comparison from the Kassab site only.

8.7.1.7 Biological Markers, ECG, Audiometry and Urinalysis

For biological measurements taken repeatedly during treatment, the mean difference in parameter value between the EOT and baseline, by randomisation arm, was presented for each parameter. At the EOT, continuous data measurement for the two PM test arms were compared by non-parametric testing by Wilcoxon rank sum tests. This was done for informative reasons even though the study was not designed as a comparative

study. The analysis of the data for part II had followed the format of part I and comparisons and methods used were decided prior to analysis.

All available data were used in this analysis. Data collection was ceased in the CRF when a patient was withdrawn from treatment due to a serious adverse event. It is important to note that other patients may have experienced an AE that led to a large change in one of the parameters included in the analysis; such values could influence results.

ECG data were analysed as binary data, where categories are normal (normal or clinically insignificant abnormality) and abnormal (clinically significant abnormality).
Data were to be summarised using the following proportions, where the denominator in all cases was the number of patients randomised to treatment with normal result at baseline.

- proportion of patients who had an abnormal reading at EOT
- proportion of patients who had an abnormal reading at EOT that has not returned to normal at 6 months

A chi-squared test was used to compare proportions across arms.

Patients were not expected to have negative (normal) values for the urinalysis parameters, protein and blood, at baseline. Patients who had urinalysis results at baseline were categorised based on recorded changes between baseline and end of treatment, into one of the following distinct groups, per arm

- Baseline negative, end of treatment negative
- Baseline negative, end of treatment positive
- Baseline positive, end of treatment positive
- Baseline positive, end of treatment negative
- Baseline negative, end of treatment missing
- Baseline positive, end of treatment missing

Note that this analysis assumes that a negative reading indicates a normal result and positive reading indicates an abnormal result.

The number of patients experiencing an audiometric shift or disabling hearing impairment at the EOT was presented by arm, along with any clinically significant findings remaining at 6 months post EOT.

Data for the original PM dose from the Kassab site only was presented for historical, visual comparison.

8.7.1.8 Serious and Non-serious Adverse Events

SAEs and AEs, classified according to MedDRA, version 10, were tabulated by treatment arm according to their corresponding System Organ Class and preferred terms.

Relation to study drug was classified as *not related* where original recording in CRF indicates unrelated and classified as *related* where recording indicates a possible, probable or unlikely relation.

Treatment emergent AEs were defined as those beginning at any time between the first

day of treatment and 30 days after the expected end of treatment inclusive. For the analysis of treatment emergent AEs, the AE rate was calculated as the number of events divided by the person-days at risk, for each arm and comparisons made across arms. The maximum person-time at risk for each arm was defined as follows,

20 mg/kg for 21 days: 21 days treatment + 30 days = 51 days

15 mg/kg for 28 days: 28 days treatment + 30 days = 58 days

Rate ratios and corresponding 95% CIs were calculated for each regimen and 95% CIs compared visually for overlapping intervals.

AEs for the original PM dose from the Kassab site was shown for historical comparison only.

8.7.2 Determination of Sample Size

See Section 8.7.1.

8.8 CHANGES IN THE STUDY CONDUCT OR PLANNED ANALYSES

No changes were made to the conduct of the study during Part II of the trial. It was carried out in the same way as Part I but patient recruitment restricted to one trial site.

9 STUDY PATIENTS

9.1 Disposition of Patients

One hundred and four patients were screened for entry into this study. Patients were excluded from the study because they were VL parasite negative (n=35), refused consent (n=2), were <4 or >60 years of age (n=16), were pregnant or lactating (n=4), had abnormal biological safety parameters (n=2), or were HIV-positive (n=3). Thus, 42 were enrolled and randomised (21 in each group).

All of the patients remained in the trial until the EOT. One patient in the 20 mg/kg group was lost at the 6-month follow-up, and 4 patients in each group received rescue medication by the 6-month follow-up (Figure 9-1).

Figure 9-1 Patient flow in LEAP 0104 Part II

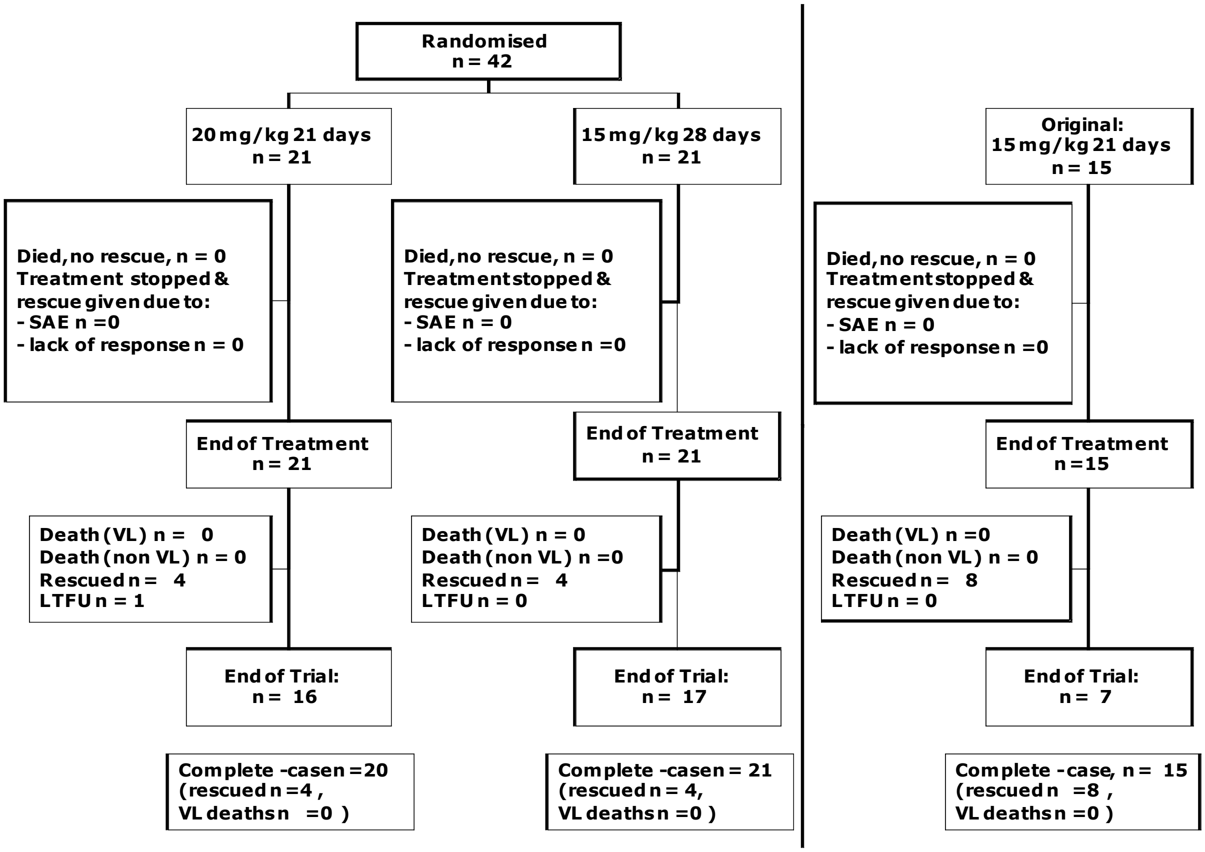

LTFU = loss to follow -up
Patient flow for original PM dose in Kassab shown for informal visual comparison

For the PK study, four patients in the 20 mg/kg group and six in the 15 mg/kg group, participated in the study.

9.2 Protocol Deviation

There were no major protocol violations. There were six minor protocol violations. Two patients (9.5%) who were severely malnourished were included in the study (both in the 20 mg/kg group), and two patients with abnormal creatinine concentrations (<30 or >130 μmol/L) and liver function test outside the normal range (SGOT/SGPT>200U/L) were included in each group. These patients were included in all analyses.

10 EFFICACY EVALUATION

10.1 Data Sets Analysed

Analyses were performed on the intent-to-treat population and all available data included.

10.2 Demographic and Other Baseline Characteristics

All of the patients that participated in this study were Sudanese from the Gedaref State. Children aged less than 15 years constituted 47.6% of patients in the 20 mg/kg group

and 38.1% of patients in 15 mg/kg group. Two thirds of patients in the 20 mg/kg group and approximately 90% in the 15 mg/kg group were male, (Table 10-1).

Table 10-1 Demographic Characteristic of Patients

		20 mg/kg/day - 21 days N=21	15 mg/kg/day - 28 days N=21	15 mg/kg/day-21 days (LEAP 0104 Part I) N=15
**Age (years)**	**Mean (SD)***	19.4 (12.3)	17.7 (9.8)	15.5 (8.1)
	**Median (IQR)***	16 (9 to 27)	17 (10 to 23)	14 (8 to 23)
	**4-14†, n (%)**	10 (47.6)	8 (38.1)	8 (53.3)
	**15-16, n (%)**	11 (52.4)	13 (61.9)	7 (46.7)
**Sex***	**Male, n (%)**	16 (76.2)	19 (90.5)	7 (46.7)
	**Female, n (%)**	5 (23.8)	2 (9.5)	8 (53.3)

*No significant difference ($p>0.1$) in age or sex distribution of patients between groups; †children classified as 4-14 years old and adults, 15 years and above.

Baseline demographic characteristics and biological parameters were similar in the two groups (Table 10-1 to Table 10-3). One patient in the 20 mg/kg group had pneumonia at baseline. Individual patient baseline data including demographic characteristics and biological parameters are shown in Section 15.2.1.
All of the patients in both groups had normal hearing and ECG measurements at baseline.

Table 10-2 Baseline Vital Sign and Other Physical and Clinical Measurements

		20 mg/kg/day - 21 days N = 21	15 mg/kg/day - 28 days N = 21	p-value*
**Temperature (°C)**	mean (SD)	38.93 (0.6)	38.97 (0.6)	0.815
	median (IQR)	39 (38.7 to 39.3)	39 (38.5 to 39.4)	0.890
**Heart Rate, (beats/min)**	mean (SD)	106.2 (13.2)	105.4 (12.1)	0.846
	median (IQR)	108 (104 to 113)	108 (98 to 112)	0.762
**Spleen Size (cm)**	mean (SD)	5.9 (3.3)	5.5 (3.6)	0.754
	median (IQR)	6 (4 to 8)	5 (3 to 8)	0.629
**Liver Size (cm)**	mean (SD)	2.9 (3.0)	3.0 (2.0)	0.810
	median (IQR)	2 (0 to 4)	4 (2 to 4)	0.400
**Systolic BP (mm Hg)**	mean (SD)	100.7 (12.1)	99.0 (12.6)	0.664
	median (IQR)	100 (90 to 110)	100 (90 to 110)	0.690
**Diastolic BP (mm Hg)**	mean (SD)	61.9 (9.7)	59.8 (10.4)	0.494
	median (IQR)	60 (50 to 70)	60 (50 to 70)	0.622
**Nutritional Status**†	Severely underweight	2 (9.5)	0	0.468
	Underweight	10 (47.6)	13 (61.9)	
	Normal	9 (42.9)	8 (38.1)	

BP = blood pressure; *p-value from testing between randomised groups, parametric ANOVA test for continuous data where mean (SD) presented, non-parametric Wilcoxon ranksum test for continuous data where median (IQR) presented, chi-squared/Fishers test for categorical data; † Based on Weight for Height if age (<6years) and BMI for Age if age (6-19years), defined as severely underweight if z-score<-3SD; underweight if -3SD≤ z-score <-2SD; normal if -2SD≤z-score<+1SD: and BMI if age (>19years): defined as severely underweight if < 16, underweight: 16.0 – 18.4, normal: 18.5 – 24.9.

Table 10-3 Baseline Laboratory Values

Laboratory parameters		20 mg/kg 21 days N = 21	15 mg/kg 28 days N = 21	p-value*
**Log Scale Parasite Count, n (%)**	**6+**	4 (19.1)	1 (4.8)	0.352
	**5+**	0	0	
	**4+**	1 (4.8)	2 (9.5)	
	**3+**	1 (4.8)	2 (9.5)	
	**2+**	1 (4.8)	4 (19.1)	
	**1+**	14 (66.7)	12 (57.1)	
**Haemoglobin (g/dl)**	**mean (SD)**	7.6 (1.4)	7.7 (0.9)	0.655
	**median (IQR)**	7.3 (7.0 to 8.1)	7.8 (6.8 to 8.4)	0.521
**White-cell Count ($x10^3$/μL)**	**mean (SD)**	3.1 (1.2)	2.8 (0.9)	0.269
	**median (IQR)**	2.8 (2.5 to 3.4)	2.6 (2.2 to 3.0)	0.371
**Platelets ($x10^3$/μL)**	**mean (SD)**	311.6 (165.8)	251.7 (89.5)	0.153
	**median (IQR)**	243 (217 to 368)	246 (194 to 320)	0.421
**AST, (U/L)**	**mean (SD)**	18.8 (5.3)	19.9 (5.2)	0.498
	**median (IQR)**	17 (14.5 to 20.0)	19.5 (15.6 to 24.0)	0.385
**ALT, (U/L)**	**mean (SD)**	18.1 (4.5)	18.8 (4.7)	0.603
	**median (IQR)**	16 (14.1 to 22.0)	18 (15 to 20)	0.384
**Bilirubin, (μmol/L)**	**mean (SD)**	12.0 (5.8)	14.0 (10.4)	0.437
	**median (IQR)**	10.3 (8.6 to 13.7)	12.0 (8.6 to 15.4)	0.493
**BUN, (mmol/L)**	**mean (SD)**	9.7 (3.1)	9.5 (3.8)	0.848
	**median (IQR)**	9.5 (7.3 to 12.0)	9.5 (6.2 to 12.8)	0.715
**Creatinine (μmol/L)**	**mean (SD)**	55.1 (15.8)	64.4 (19.4)	0.098
	**median (IQR)**	53.0 (44.2 to 61.9)	61.9 (44.2 to 88.4)	0.148

AST, Aspartate aminotransferase; ALT, Alanine aminotransferase; BUN, Blood urea nitrogen;*p-value from testing between randomised groups; parametric ANOVA test for continuous data where mean (SD) presented, non-parametric Wilcoxon ranksum test for continuous data where median (IQR) presented, chi-squared/Fishers test for categorical data.

10.3 Measurements of Treatment Compliance

Patients remained in the hospital for the duration of treatment and no issues with compliance with treatment were reported at EOT.

Approximately half of the patients in each arm were followed up within 14 days of the 6-month follow-up date (Table 10-4). One patient in the 20 mg/kg group was lost to follow-up but all of the rest were seen within 45 days of the 6-month follow-up date.

Table 10-4 Timing of the 6-month follow-up, n (%)

Final evaluation	20 mg/kg 21 days<br>N = 21	15 mg/kg 28 days<br>N = 21
Within 2 weeks of expected date	12 (57.1)	12 (57.1)
>2 weeks to <1.5 months	8 (38.1)	8 (38.1)
1.5 to 3 months after expected date	0	1 (4.8)
Loss to Follow Up	1 (4.8)	0

10.4 Efficacy Results and Tabulations of Individual Patient Data

10.4.1 Analysis of Efficacy

10.4.1.1 Primary Efficacy Endpoint

Analysis of the primary efficacy endpoint showed that 18 patients in the 20 mg/kg group and 19 patients in the 15 mg/kg group had parasite clearance at EOT. Thus, end of treatment data shows efficacy of between 85% and 90% (Table 10-5). Corresponding 95% CI around the efficacy estimates in each arm are very similar (Table 10-5).

Since there were no missing data at EOT, worst case analysis results did not differ from the complete-case analysis.

Table 10-5 End of Treatment Analysis (Complete Case Analysis)

Efficacy	20 mg/kg/day - 21 days N=21	15 mg/kg/day - 28 days N=21	15 mg/kg/day-21 days (LEAP 0104 Part I) N=15
**Treatment efficacy at end of treatment, n (%)**	18 (85.7)	19 (90.5)	9 (60.0)
**95% CI for efficacy, %**	[63.7; 97.0]	[69.6; 98.8]	[32.3; 83.7]

CI = confidence interval

10.4.1.2 Secondary Efficacy Endpoint

Secondary efficacy endpoints included detection of parasites in patients who were clinically unwell at 3 months and parasite count at 6 months. Eighteen patients in the 20 mg/kg group and 20 patients in the 15 mg/kg group were followed up at 3 months (Table 10-6). Of these, 16 patients in the 20 mg/kg group and 17 in the 15 mg/kg group were considered to be clinically well and had no parasitology performed. Two patients in the 20 mg/kg group and three patients in the 15 mg/kg group were not clinically well and parasites were detected in all of them indicating a relapse rate of 11.1% and 15.8%, respectively (Table 10-6).

Table 10-6 3-Month Secondary Efficacy Endpoint

	20 mg/kg/day - 21 days N=21	15 mg/kg/day - 28 days N=21	15 mg/kg/day-21 days (LEAP 0104 Part I) N=15
Patients seen at 3 months & assumed to be clinically well*	16 (76.2)	17 (81.0)	8 (53.3)
Patients seen at 3 months & parasites detected at 3 months	2 (9.5)	3 (14.3)	0
Patients seen at 3 months & in whom parasites had not been detected at EOT	18 (85.7)	19 (90.5)	7 (46.7)
Relapses†	2 (11.1)	3 (15.8)	0

EOT = End of Treatment, day 22 or 29; *Clinically well patients did not have parasitological examination; †Relapse, parasites not detected at EOT but detected at 3 months

At the 6-month follow up, 16 and 17 patients in the 20 mg/kg group and the 15 mg/kg groups, respectively were parasite-free, indicating a DC rate of 80% in both groups for the complete case analysis (Table 10-7). One patient in the 15 mg/kg group who was parasite positive at EOT had parasite clearance at the 6-month follow-up, indicating a slow response to treatment.

Table 10-7 6-Month Secondary Efficacy Endpoint: Complete Case Analysis

Efficacy	20 mg/kg/day - 21 days N=20	15 mg/kg/day - 28 days N=21	15 mg/kg/day-21 days (LEAP 0104 Part I) N=15
**Treatment efficacy at 6 months, n (%)**	16 (80.0)	17 (81.0)	7 (46.7)
**95% CI for efficacy, %**	[56.3-94.3]	[58.1-94.6]	[21.3; 73.4]

Table 10-8 6-Month Secondary Efficacy Endpoint: Last Parasitology Carried Forward

	20 mg/kg/day - 21 days N=21	15 mg/kg/day - 28 days N=21	15 mg/kg/day-21 days (LEAP 0104 Part I) N=15
**Treatment efficacy at 6 months, n (%)**	16 (76.2)	17 (81.0)	7 (46.7)
**95% CI for efficacy, %**	[52.8-91.8]	[58.1-94.6]	[21.3; 73.4]

One patient who was lost to follow-up had parasites at end of treatment and was considered treatment failure

Four patients in each group received rescue medication by 6 months, and one patient in the 20 mg/kg group was lost to follow-up (Figure 9-1). Since the patient who was lost to follow-up was also parasite-positive at EOT, results of the LPCF analysis and worst case

analysis were identical. Parasite infection resolved in all of the patients who received rescue medication (four patients in each group).

10.4.2 Statistical/Analytical Issues

10.4.2.1 Adjustment for Covariates

No adjustments were made.

10.4.2.2 Handling of Dropouts or Missing Data

The efficacy in each arm is the proportion, or percentage, of treatment success. To take account of missing data, efficacy analyses were carried out at EOT and 6 months, within an intention-to treat framework, in three ways;

- Complete case analysis: excluding patients with missing efficacy data from estimation of efficacy in each arm
- Worst case analysis: Patients with missing efficacy data were allocated efficacy results based on their 'worst-case' scenario i.e. treatment failure.
- Last parasitology carried forward (LPCF): Patients with missing efficacy data had their last parasitology results carried forward (relevant for 6-month data only).

In the complete-case analysis, missing efficacy data were replaced as treatment failures in the following cases, since these events could not be assumed to be independent of VL or the study drug;

- Death during initial hospitalization period, before or after receipt of rescue
- Stopping of study treatment due to SAE or lack of response to treatment
- Death during follow-up where independence to VL could not be confirmed by the medical co-ordinator

Stopping treatment due to a protocol violation or, investigator error in not conducting parasitological exam was not assumed to indicate treatment failure in a complete-case analysis.

Primary efficacy analysis was carried out on complete case analysis and worst case analyses, whereas the 6 months efficacy analyses were carried out with complete case analysis, worst case analyses, and LPCF.

10.4.2.3 Interim Analyses and Data Monitoring

No interim analyses were planned or performed.

10.4.3 Tabulations of Individual Response Data

Efficacy was based on the single variable of parasite clearance at EOT and at 6 months. Individual tabulated data are given in section 15.2.3.

10.4.4 Drug dose, Drug Concentration, and Relationships to Dose

Data were available from four patients in the 20 mg/kg group and six patients in the15 mg/kg group. Since few patients participated in this part of the study, the results should be viewed with caution as an indicator only of what might be expected in patients.

Assay of PM in plasma and urine from patients

The limit of PM detection in the current study was 0.05 μg/ml and the limit of quantification was 0.1 μg/ml. There was no significant difference in mean PM plasma concentration between the two groups (Figure 10-1, Section 15.1.8) In most patients PM was undetectable beyond 8 h on all days sampled in both groups. Due to variability in plasma PM concentrations in both groups, area under the plasma concentration-time curves (0-8 h), T_{max} and C_{max} could not be reliably be determined.

Figure 10-1 Mean Plasma Paromomycin Concentration Following a Single I.M. Administration

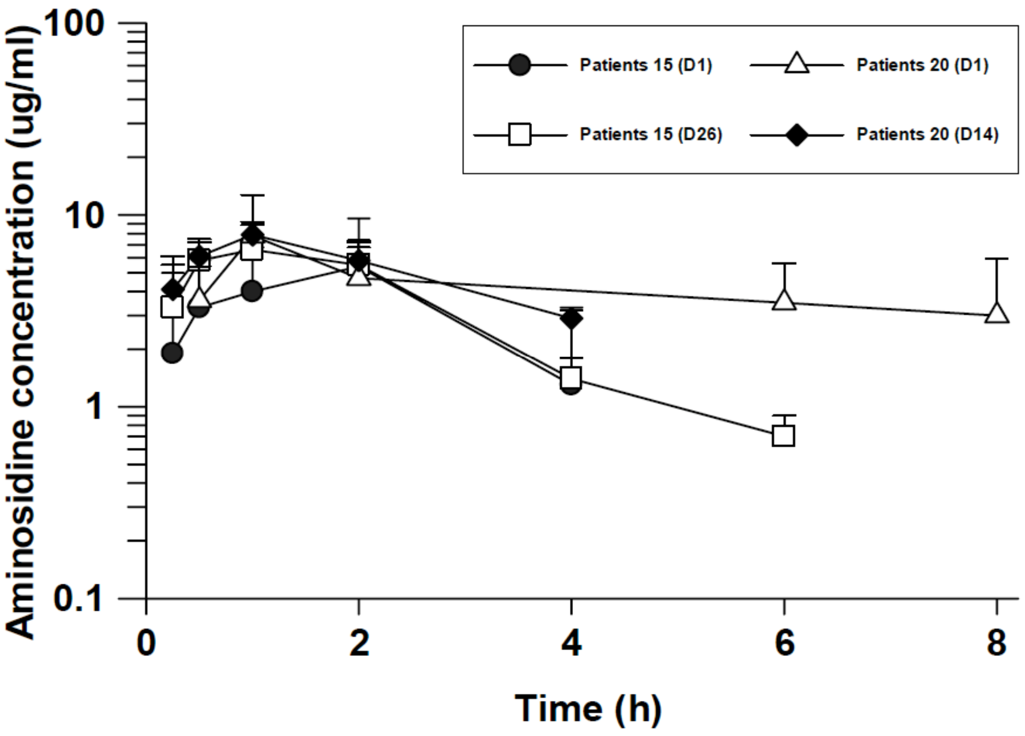

Measurements made on days 1 and 14 (for patients receiving 20 mg/kg) and on days 1 and 26 (for patients receiving 15 mg/kg). Error bars indicate standard deviation.

The results showed wide inter-subject variability at each time point. PM was rapidly absorbed into the circulation with peak plasma concentrations achieved at 1-2 hours post-dosing and also rapidly and completely excreted in urine, being undetectable in plasma 8 hours after dosing in patients (see report, Section 15.1.8). No differences in PK between the 20 mg/kg and 15 mg/kg groups were observed, which could be due to the small samples size and wide inter-subject variability. More PK studies are planned and will be reported in LEAP 0104 Part III.

10.4.5 Drug-Drug and Drug-Disease Interaction

A list of concomitant drugs were administered is presented in Section 15.2.8. There were no reports of the interaction of PM with other drugs.

10.4.6 Efficacy Conclusion

Daily dosing with either 20 mg/kg of PM for 21 days or 15 mg/kg of PM for 28 days resulted in parasite clearance in 85% and 90% of VL patients, respectively at EOT. In both arms, there was a 95% probability that the efficacy of both treatments was between approximately 55% and 95% at the 6-month follow-up.

This was an improvement compared with the efficacy observed at EOT in Kenya and Sudan only. Using all available EOT data from these sites, treatment efficacy in patients allocated to 15 mg/kg/day for 21 days and 95% confidence bounds suggested efficacy between 40 and 66% (LEAP 0104 Part I results).

Thus, the primary objective of identifying a more efficacious dose of PM was met. Baseline characteristics of patients in both groups were similar. However, it is important to bear in mind that because of the low number of patients enrolled, the efficacy of the two doses cannot be formally compared.

11 SAFETY EVALUATION

11.1 Extent of Exposure

All of the patients (21 per group) received their assigned daily dose of PM for the duration of the treatment period, i.e. 20 mg/kg/day for 21 days and 15 mg/kg/day for 28 days. Daily (Table 11-1) or total (Table 11-2) PM exposure data are presented for children and adults separately.

Table 11-1 Paromomycin Daily Dose per Day

Daily dose (mg)		20 mg/kg	15 mg/kg
Children (4-14 years)	Mean (SD)	407.6 (159.2)	348.0 (190.5)
	Median (IQR)	350 (300 to 450)	292.5 (221.5 to 416.3)
Adults (15 years and above)	Mean (SD)	997.2 (137.8)	730.3 (156.3)
	Median (IQR)	1014.3 (866.7 to 1088.6)	743.6 (660 to 826.8)

ie mean of individual daily doses per patient; SD=standard deviation; IQR=inter-quartile range

Table 11-2 Total Paromomycin Dose Received During the Study

Total dose (mg)		20 mg/kg - 21 days	15 mg/kg - 28 days
Children (4-14 years)	Mean (SD)	8559.5 (3342.6)	9743.1 (5334.6)
	Median (IQR)	7350 (6300 to 9450)	8190 (6195 to 11657.5)
Adults (15 years and above)	Mean (SD)	20940.9 (2894.1)	20448.8 (4375.8)
	Median (IQR)	21300 (18200 to 22860)	20820 (18480 to 23150)

ie Total individual daily doses per patient; SD=standard deviation; IQR=inter-quartile range

11.2 Adverse Events

11.2.1 Brief Summary of Adverse Events

A total of 48 AEs were reported in 33 patients (from both groups) during the study (Table 11-3). Most of the AEs were treatment-emergent and were assessed as being related to the drug. No SAEs were reported in this study. AE rates and corresponding 95% CIs show very similar rates in each group.

Table 11-3 Number of Adverse Events (AEs) and Number of Patients Experiencing AEs During the Study

	20 mg/kg/day - 21 days	15 mg/kg/day - 28 days
**Number of patients who received drug**	21	21
**Patient–days on treatment***	1071	1218
**Total number of AEs (number of patients with AEs)‡**	20 (15)	28 (18)
**Number of AEs during follow-up (number of patients)**	1 (1)	4 (4)
**Number of treatment-emergent AEs† (number of patients)**	19 (15)	24 (17)
**AE rate**	0.018	0.020
**95% CI around rate**	0.011 to 0.028	0.013 to 0.029

*Patient-days = 21 x (days on treatment +30) ;‡Not all patient experienced AEs and some patients experienced more than one AE ; †Treatment-emergent AE defined as AE onset between day 1 of treatment to 30 days post-treatment; CI, confidence interval.

11.2.2 Display of AEs

Most of the AEs that occurred during the study were related to administration site conditions (Table 11-4). Listing of AEs by patient is presented in Section 15.2.4.

Table 11-4 All Non-serious Adverse Events by Treatment and Relation to Drug, n (%)

**System Organ Class**<br>Preferred term	**20 mg/kg/day - 21 days**		**15 mg/kg/day - 28 days**	
	**NR**	**ADR**	**NR**	**ADR**
**Gastro-intestinal Disorders**	**1**	**0**	**0**	**0**
Diarrhoea	1	0	0	0
**General Disorders and Administration Site Conditions**	**0**	**14 (77.8)**	**0**	**16 (57.1)**
Injection Site Pain	0	14	0	16
**Investigations**	**0**	**3 (16.7)**	**0**	**3 (10.7)**
Audiogram Abnormal	0	3	0	3
**Infections and Infestations**	**1**	**1 (5.6)**	**0**	**6 (21.4)**
Malaria	1	0	0	1
Otitis media	0	0	0	0
Otitis media acute	0	0	0	1
Viceral Leishmaniasis*	0	1	0	4
Urinary tract infection	0	0	0	0
**Respiratory, Thoracic and Mediastinal Disorders**	**0**	**0**	**0**	**1 (3.6)**
Epistaxis	0	0	0	1
**Hepatobiliary Disorders**	**0**	**0**	**0**	**1 (3.6)**
Cholestatic jaundice	0	0	0	1
**Blood and Lymphatic Disorders**	**0**	**0**	**0**	**1 (3.6)**
Jaundice	0	0	0	1
**Total**	**2 (100)**	**18 (100)**	**0**	**28 (100)**

NR, not related; ADR, adverse drug reaction; *Post kala-azar dermal leishmaniasis

11.2.3 Analysis of AEs

The most common AE in both treatment arms was injection site pain, and this was considered to be drug-related. Considering that patients received daily i.m. injections for more than 20 days this was not unexpected. Three patients in each group had abnormal audiograms at EOT referring to audiometric shifts presented earlier, which were considered to be related to the drug. In all patients but one in the 20 mg/kg group, this resolved by the 6-month follow-up. The types of AEs experienced by patients in both groups were similar but there were more cases of PKDL (coded as visceral Leishmaniasis) reported in the 15 mg/kg group.

11.3 Deaths, Other Serious Adverse Events, and Other Significant AEs

11.3.1 Listings of deaths, other serious adverse events, and other significant AEs

There were no deaths or other SAEs reported during the study. One patient in the 20 mg/kg group had disabling hearing impairment at EOT. However, the hearing

impairment improved by 6 months, and patient was recorded as having an audiometric shift.

11.3.2 Narratives of deaths, other serious adverse events, and other significant adverse events

No deaths or other SAEs were reported during this study. Three patients in each treatment group had abnormal audiometric measurements at EOT (see Section 11.5). In one patient in the 20 mg/kg group, the audiometric shift was considered to be disabling hearing impairment. The audiometric shift in five patients resolved at the 6-month follow-up. The disabling hearing impairment at EOT in one patient had not completely resolved at 6 months, though was no longer considered disabling (see Table 11-8).

11.3.3 Analysis and discussion of deaths, other SAEs, and other significant AEs

See Section 11.3.2.

11.4 Clinical Laboratory Evaluation

Listing of individual laboratory measurements by patient are presented in Section 15.2.6.

11.4.1 Evaluation of Laboratory Parameters

Laboratory values for the various parameters tested at EOT were similar in the two treatment arms. The only exception was haemoglobin concentration, which showed weak evidence of lower average values in the 20 mg/kg group compared with the 15 mg/kg group ($p = 0.028$).

Table 11-5 Clinical Laboratory Values at End of Treatment

	20 mg/kg/day - 21 days		15 mg/kg/day - 28 days			
	**Mean (SD)**	**Median (IQR)**	**Mean (SD)**	**Median (IQR)**	**p-value***	**p-value†**
**Haemoblobin (g/dl)**	9.1 (1.0)	9.1 (8.5-9.8)	9.7 (0.7)	9.7 (9.1-10.1)	0.029	0.028
**White Blood Cell Count ($x10^3/\mu l$)**	4.4 (1.2)	4 (3.5-5.1)	4.3 (1.0)	4.1 (3.6-5.0)	0.947	0.880
**Platelet Count ($x10^3/\mu l$)**	336.1 (94.2)	312 (286-368)	366.7 (99.9)	387 (310-410)	0.080	0.208
**AST (U/L)**	17.0 (2.4)	16.7 (15-18.6)	17.7 (3.3)	18 (16-19.2)	0.627	0.487
**ALT (U/L)**	16.5 (2.6)	16.8 (14.9-18.4)	17.5 (2.7)	18 (15-19)	0.333	0.413
**Bilirubin (μmol/L)**	11.2(3.8)	10.3 (8.6-12.0)	10.8 (4.9)	10.3 (8.6-12)	0.584	0.497
**BUN (mmol/L)**	8.1 (1.9)	8.0 (7.3-8.8)	7.9 (2.3)	7.3 (6.6-8.8)	0.828	0.324
**Creatinine (μmol/L)**	51.4 (15.7)	44.2 (35.4-61.9)	48.8 (15.5)	44.2 (44.2-44.2)	0.558	0.563

*ANCOVA testing for a difference in end of treatment mean measurements between randomised groups, adjusting for baseline values; †Wilcoxon ranksum test for difference in end of treatment median measurements between randomised groups

Urinalysis showed that there were no abnormalities in protein and blood in urine that appeared during treatment (Table 11-6). Nine patients in the 20 mg/kg group and five in the 15 mg/kg group who had abnormal protein concentrations at baseline also had abnormal concentrations at EOT. Abnormal protein concentrations at baseline in nine patients in the 20 mg/kg group and 15 in the 15 mg/kg group, had resolved at EOT. Similarly, there were no abnormal blood values that appeared during treatment, although these measurements were missing in four patients in the 15 mg/kg group (Table 11-6).

Table 11-6 Comparison of Urinalysis Outcome between Baseline and End of Treatment, n (%)

	20 mg/kg/day - 21 days N=21	15 mg/kg/day - 28 days N=21
**Protein**		
**Baseline negative, EOT negative**	3 (14.3)	1 (4.8)
**Baseline negative, EOT positive**	0	0
**Baseline positive, EOT positive**	9 (42.9)	5 (23.8)
**Baseline positive, EOT negative**	9 (42.9)	15 (71.4)
**Baseline negative, EOT missing**	0	0
**Baseline positive, EOT missing**	0	0
**Blood***		
**Baseline negative, EOT negative**	6 (28.6)	8 (38.1)
**Baseline negative, EOT positive**	0	0
**Baseline positive, EOT positive**	0	0
**Baseline positive, EOT negative**	0	0
**Baseline negative, EOT missing**	2 (9.5)	4 (19.1)
**Baseline positive, EOT missing**	0	0

* Missing values for 22 patients at baseline; 13 in the 20 mg/kg group and 9 in the 15 mg/kg group

11.5 Vital Signs, Physical Findings, and other Observations Related to Safety

No abnormal ECG measurements were reported in any of the patients during treatment or follow-up. A total of six patients had audiometric shift at EOT (Table 11-8). Of these one was considered to be disabling hearing impairment. At the 6-month follow-up these audiometric abnormalities had resolved in five patients. One patient had audiometric shift at the 6-month follow-up.

Table 11-7 Vital signs and Other Physical Measurements at End of Treatment

	20 mg/kg/day - 21 days, N=21		15 mg/kg/day - 28 days, N=21			
	**Mean (SD)**	**Median (IQR)**	**Mean (SD)**	**Median (IQR)**	**p-value***	**p-value†**
**Temperature (°C)**	36.1 (0.3)	36.2 (36-36.2)	36.0 (0.5)	36.2 (35.8-36.2)	0.381	0.664
**Heart Rate (beats/min)**	88.6 (10.2)	90 (82-92)	86.7 (11.7)	84 (78-92)	0.604	0.550
**Spleen Size (cm)**	0.8 (1.3)	0 (0-2)	1.0 (1.9)	0 (0-2)	0.472	0.816
**Liver Size (cm)**	0.6 (1.3)	0 (0-0)	0.3 (0.7)	0 (0-0)	0.221	0.600
**Systolic BP (mm/Hg)**	105 (12.9)	110 (95-120)	103.3 (13.1)	100 (90-115)	0.928	0.682
**Diastolic BP (mm/Hg)**	63.3 (10.0)	60 (55-70)	63.1 (9.3)	65 (60-70)	0.557	0.969

SD, standard deviation; IQR, interquartile range; BP, blood pressure; *p-value from ANCOVA testing for a difference in end of treatment measurements between the two treatment arms adjusted for baseline, † p-value from Wilcoxon ranksum test for difference in end of treatment measurements between the two treatment arms.

Table 11-8 Audiometric Results* During Treatment and Follow-up

	**(20 mg/kg/day – 21 days) N=21**	**(15 mg/kg/day – 28 days) N=21**
Patients with a shift at EOT†, n (%)	3 (14.3)	3 (14.3)
Patient with disabling hearing impairment at EOT	1 (4.8)	0
Patients with a shift remaining at 6-month follow-up†‡	1 (4.8)	0
Patient with disabling hearing loss remaining at 6-months follow-up‡	0	0

EOT, end of treatment; *Not including minor audiometric protocol violation at baseline; †shift of ≥25dB at ≥one frequency or ≥20dB at ≥two adjacent frequencies, in either ear; ‡disabling hearing impairment (average of ≥41dB in adults or ≥31dB in children over the frequencies 500Hz, 1000Hz, 2000Hz and 4000 Hz) detected at end of treatment, not returning to normal levels by end of trial.

11.6 Safety Conclusions

There were no unexpected safety issues that arose during this study. There were no deaths or SAEs. The most common AE in both treatment groups was pain at the site of injection related to the administration of treatment. Audiometric shift associated with the

drug was reported in six patients. Although this was completely reversible in five patients, one patient who had received the higher dose still had had audiometric shift at the 6-month follow-up. Though these numbers are low they suggest that ototoxicity is an important issue that needs to be closely monitored.

No other significant laboratory findings or changes in vital sign or other physical measurements were identified during the study.

12 Discussion and Overall Conclusions

The goal of the current study was to evaluate the efficacy and safety of PM in Sudanese VL patients, to identify an effective dose for Part III of the LEAP 0104 study. The results from the two treatment regimens (20 mg/kg/day for 21 days and 15 mg/kg/day for 28 days) showed improved and acceptable efficacy compared with the results of Part I of LEAP 0104.

The primary efficacy endpoint in the current study was parasite clearance at EOT. This was different from Part I of LEAP 0104, in which the primary endpoint was parasite clearance at 6 months. Since few patients were enrolled in the current study it was considered that the value of the study would have been diminished if parasite clearance at 6 months were the primary endpoint since some patients were expected to be lost at follow-up, further reducing patient numbers data available for evaluation. Furthermore, the study was not powered to detect differences between the two treatment arms and no conclusion can be drawn from the absence of a difference between the treatment arms.

All of the patients enrolled received treatment for the duration of the study and data from all patients were available at EOT. At EOT, efficacy rate was 85.7% (95% CI: 63.7%-97.0%) and 90.5% (95% CI: 69.6%-98.8%) in the 20 mg/kg and 15 mg/kg treatment arms, respectively. At the 6-month follow up, efficacy was about 80% in both groups (complete case analysis, 95% CI: 52.8% to 91.8% and 58.1% to 94.6% in the 20 mg/kg and 15 mg/kg groups, respectively) and was only slightly lower in the worst case analyses. Both results are better than the efficacy observed in Sudan with 15 mg/kg/day for 21 days (< 50% efficacy), and comparable to what was observed at the similar time point in Kenya in Part I of the LEAP 0104 study (80% efficacy at 6 months). However, this was not as good as what was reported in India (95% cure rate at 6 months) (Sundar et al. 2007).

No major unexpected safety issues arose during this study. Ototoxicity associated with the treatment was reported. Although it resolved in most of the cases one patient still had hearing impairment at 6 months. Ototoxicity is a know transient side effect of PM. The observation that it had not completely resolved in one case (2.4% of the treated patients) suggests that the safety of PM with regard to ototoxicity needs to be closely monitored. By contrast, no abnormal treatment-emergent audiometry was reported in LEAP 0104 Part I, suggesting that the risk for ototoxicity may be of concern at the higher doses.

There was no difference in the mean plasma PM concentration between the two groups.

Although the PK analysis was performed in very few patients, the data offer some explanation for the poor efficacy results seen in Sudan, in contrast to the good results seen in Indian patients using the same dose, raising the possibility that Sudanese patients may metabolise and excrete the drug differently. Our limited PK studies show that peak plasma PM concentrations in patients in the current study were lower than what was reported among Indian patients (Sundar et al., 2007). The mean ±SD peak PM concentration following administration of 15 mg/kg in the current study was between

5.4 μg/ml ± 4.2 and 6.6 μg/ml ± 2.3 as compared with a peak concentration of between 18.3 μg/ml ±8.9 to 20.5 μg/ml ± 7.1 among patients who received the equivalent of 15 mg/kg PM (i.e 11 mg/kg paromomycin free base) in the study conducted in India. These results suggest that in Sudanese patients plasma PM concentrations never reach the high level observed in the studies in India and may offer a possible explanation for the differences in efficacy. However, due to the limited number of patients in this study, the results have to be confirmed. A larger PK study was planned and conducted as part of Part III of the trial.

In conclusion, the current study showed that both treatments with higher total PM dose were more efficacious than the dose initially used in LEAP 0104 Part 1. The treatment with the shorter duration, i.e. 20 mg/kg/day for 21, was selected as the dose for comparison with other treatments in Part III of the LEAP 0104 study.

13 Tables, Figures, and Graphs referred to but not included in the Text

None

14 Reference List

References

Desjeux, P. (1996). "Leishmaniasis. Public health aspects and control." Clin Dermatol 14(5): 417-23.

Jha, T. K., P. Olliaro, et al. (1998). "Randomised controlled trial of aminosidine (paromomycin) v sodium stibogluconate for treating visceral leishmaniasis in North Bihar, India." BMJ 316(7139): 1200-5.

Kanyok, T. P., Killian, A. D. et al. (1997). "Pharmacokinetics of intramuscularly administered aminosidine in healthy subjects." Antimicrob Agents Chemother 41 (5): 982-6.

Ritmeijer, K. and R. N. Davidson (2003). "Royal Society of Tropical Medicine and Hygiene joint meeting with Medecins Sans Frontieres at Manson House, London, 20 March 2003: field research in humanitarian medical programmes. Medecins Sans Frontieres interventions against kala-azar in the Sudan, 1989-2003." Trans R Soc Trop Med Hyg 97(6): 609-13.

Seaman, J., A. J. Mercer, et al. (1996). "The epidemic of visceral leishmaniasis in western Upper Nile, southern Sudan: course and impact from 1984 to 1994." Int J Epidemiol 25(4): 862-71.

Seaman, J., D. Pryce, et al. (1993). "Epidemic visceral leishmaniasis in Sudan: a randomized trial of aminosidine plus sodium stibogluconate versus sodium stibogluconate alone." J Infect Dis 168(3): 715-20.

Thakur, C. P., T. P. Kanyok, et al. (2000a). "Treatment of visceral leishmaniasis with injectable paromomycin (aminosidine). An open-label randomized phase-II clinical study." Trans R Soc Trop Med Hyg 94(4): 432-3.

Thakur, C. P., T. P. Kanyok, et al. (2000b). "A prospective randomized, comparative, open-label trial of the safety and efficacy of paromomycin (aminosidine) plus sodium stibogluconate versus sodium stibogluconate alone for the treatment of visceral leishmaniasis." Trans R Soc Trop Med Hyg 94(4): 429-31.

WHO (2007). "Fifth Consultative Meeting on Leishmania/HIV Coinfection, Addis Ababa, Ethiopia, 20-22 March 2007." World Health Organisation, WHO/CDS/NTD/IDM/2007.5.

World Health Organization (2004). Guidelines for Hearing Aids and Services for Developing Countries. WHO. Switzerland.

Zijlstra, E. E., A. M. Musa, et al. (2003). "Post-kala-azar dermal leishmaniasis." Lancet Infect Dis 3(2): 87-98.

15 Appendices

15.1 Study Information

15.1.1 Protocol and Protocol Amendments
15.1.2 Sample Case Report Forms
15.1.3 List of Independent Ethics Committee and sample Consent Form
15.1.4 List of Investigators
15.1.5 Signatures of Investigators
15.1.6 Randomization Schedule
15.1.7 Statistical Analysis Plan
15.1.8 Pharmacokinetics Study Report

15.2 Patient Data Listings

15.2.1 Demographics and Baseline Characteristics
15.2.2 Drug Dose Administration
15.2.3 Individual Efficacy Response Data
15.2.4 Individual Patients Adverse Events Listing
15.2.5 Listing of Patients Requiring Rescue Medication
15.2.6 Individual Patient Vital Sign and Laboratory Measurements
15.2.7 Audiometry and Electrocardiogram Measurements
15.2.8 Individual Listings of Concomitant medication
