# Supplementary material for: Paromomycin for the Treatment of Visceral Leishmaniasis in Sudan: A Randomized, Open-Label, Dose-Finding Study
Source: PLoS Negl Trop Dis. 2010 Oct 26;4(10):e855. doi: 10.1371/journal.pntd.0000855 (PMC2964291; doi:10.1371/journal.pntd.0000855)
Supplement: Protocol S2 — Clinical trial protocol. (0.23 MB PDF) [file pntd.0000855.s003.pdf]

TITLE

A MULTICENTRE COMPARATIVE TRIAL OF EFFICACY AND SAFETY OF SODIUM STIBOGLUCONATE (SSG) VERSUS PAROMOMYCIN (PM) VERSUS COMBINATION OF SSG AND PM AS THE FIRST LINE TREATMENT FOR VISCERAL LEISHMANIASIS IN ETHIOPIA, KENYA AND SUDAN

Principal Investigators
Dr Monique Wasunna[1]
Dr. Asrat Hailu [2]
Dr. Getahun Mengistu [2]
Dr. Musa Amudawi[3]
Dr. Manica Balasegaram[4]

Project Manager
Dr Catherine Royce[5]

Statistician
Mr Lawrence Muthami[1]

[1]Kenya Medical Research Institute, Kenya
[2]University of Addis Ababa, Ethiopia
[3]Institute of Endemic Diseases, University of Khartoum, Sudan
[4]Médecins Sans Frontières Holland, Sudan
[5]Drugs for Neglected Diseases Initiative, Switzerland
[6]University of Nairobi, Kenya
[7]Gedarif University, Sudan
[8] National Ribat University, Sudan

Co- investigators: KENYA
Dr. J R Rashid[1]
Dr. J. Mbui[1]
Dr. P. Nyakundi[1]
Dr. G. Mucee[1]
Dr. V. Manduku[1]
Dr. A. Musibi[1]
Dr. Z. Mutuma[1]
Dr. F. Kirui[1]

Dr. H. Lodenyo[1]
Mr. D. Kinoti[1]
Prof. K. Bhatt[6]

Co-investigators: SUDAN

Professor A.M. El-Hassan[3]
Professor Eltahir Awad Gasim Khalil[3]
Dr M.E. Ibrahim[3]
Dr I.M. Elhassan[3]
Dr Ahmed Abdalla[7]
Dr Fawzi Abdeirahim Mahjoub[8]

Co- investigators: ETHIOPIA

Prof Eyasu Makonnen[2]
Dr Yalemtsehay Mekonnen[2]
Dr Shibru Berhanu [Gondar]
Dr Asfawesen Gebre-Yohannes [Gondar]
Dr Sisay Yifru [Gondar]
Dr Abiye Tesfaye [Gondar]
Dr Nurelign Gashu [Gondar]
Dr Samson Tesfaye [Arba-Minch]
Dr Yewubnesh Hailu [Arba-Minch]
Dr Degu Jerene [Arba-Minch]

SIGNATURE PAGE

**Principal Investigators**	**SIGNATURE**	**DATE**
Dr Monique Wasunna	……………………	……………….
Dr. Asrat Hailu	……………………	……………….
Dr. Getahun Mengistu	……………………	……………….
Dr. Musa Amudawi	……………………	……………….
Dr. Manica Balasegaram	……………………	……………….
**Project Manager**		
Dr Catherine Royce	……………………	……………….
**Statistician**		
Mr. Lawrence Muthami	……………………	……………….

TABLE OF CONTENTS

LEAP DRAFT PROPOSAL……………………………………………………………………………………1
TITLE……………………………………………………………………………………………………1
INVESTIGATORS…………………………………………………………………………………………1
SIGNATURE PAGE………………………………………………………………………………………3
SUMMARY………………………………………………………………………………………………6
LITERATURE REVIEW……………………………………………………………………………………6
DISTRIBUTION OF VISCERAL LEISHMANIASIS IN EAST AFRICA………………………………………7
CLINICAL ASPECTS OF LEISHMANIASIS IN EASTERN AFRICA…………………………………………8
MAIN TREATMENT OPTIONS FOR VISCERAL LEISHMANIASIS……………………………………9
NEED FOR NEW TREATMENT OPTIONS…………………………………………………………10
SSG……………………………………………………………………………………………………10
PAROMOMYCIN…………………………………………………………………………………………11
HISTORICAL PRODUCT PROFILE – FARMITALIA DOSSIER ………………………………………11
PRE-CLINICALTOXICOLOGY…………………………………………………………………………12
ANIMALTOXICOLOGY…………………………………………………………………………………13
CLINICAL PHARMACOLOGY…………………………………………………………………………15
PHARMACOKINETICS…………………………………………………………………………………15
DOSE-FINDING STUDIES………………………………………………………………………………15
CLINICAL EXPERIENCE WITH INJECTABLE PAROMOMYCIN IN THE TREATMENT OF VL…...16
TRIAL OBJECTIVES AND PURPOSE…………………………………………………………………17
HYPOTHESIS……………………………………………………………………………………………17
OBJECTIVES OF THE TRIAL……………………………………………………………………………17
METHODOLOGY…………………………………………………………………………………………18
STUDY DESIGN…………………………………………………………………………………………18
STUDY SITES……………………………………………………………………………………………18
INCLUSION CRITERIA…………………………………………………………………………………18
EXCLUSION CRITERIA…………………………………………………………………………………19
HIV-STATUS AND VCT…………………………………………………………………………………19
CRITERIA FOR PATIENT WITHDRAWAL……………………………………………………………20
SAMPLE SIZE……………………………………………………………………………………………21
TREATMENT……………………………………………………………………………………………23

DRUG ADMINISTATION……………………………………………………………………………………23
RESCUE MEDICATION…………………………………………………………………………………24
PRIOR AND CONCOMITANT MEDICATIONS…………………………………………………………24
EFFICACY ASSESSMENT…………………………………………………………………………………24
PRIMARY EFFICACY ENDPOINT…………………………………………………………………………25
SECONDARY EFFICACY ENDPOINT……………………………………………………………………25
SAFETY ASSESSMENTS…………………………………………………………………………………26
ADVERSE EVENTS…………………………………………………………………………………………26
ASSESSMENT OF INTENSITY/SEVERITY…………………………………………………………… 26
ASSESSMENT OF CASUALITY……………………………………………………………………………26
SERIOUS ADVERSE EVENTS………………………………………………………………………………27
DATA COLLECTION, STORAGE AND ANALYSIS…………………………………………………………28
QUALITY CONTROL AND QUALITY ASSURANCE…………………………………………………………29
ETHICAL CONSIDERATIONS…………………………………………………………………………………29
INSURANCE AND LIABILITY…………………………………………………………………………………30
TIME FRAME……………………………………………………………………………………………………31
REFERENCES……………………………………………………………………………………………………32
PATIENT INFORMATION AND CONSENT…………………………………………………………………35

SUMMARY

Visceral leishmaniasis (VL) or Kala-azar is the most severe form of leishmaniasis. It is estimated that 500,000 new cases world wide of VL are diagnosed annually. 90% of VL cases occur in developing countries: India (especially Bihar), Bangladesh, Nepal, North Eastern Brazil and Sudan. For the past 100 years, antimony has been the first line of treatment for VL cases despite considerable toxicity and the requirement for 4 weeks hospitalization.

Resistance to antimony coupled with emergence of HIV associated with VL is on the increase. New and improved treatment options are urgently needed to replace or complement the few currently available drugs. The wide variety of epidemiological situations and clinical presentations of this disease further warrant a series of treatment options instead of one single treatment or control strategy for the affected populations.

During 2003, experts in VL together with representatives of regulatory authorities and health ministries from Kenya, Ethiopia and Sudan met (Nairobi, May 2003, Khartoum, August 2003) to discuss the development of new treatment options for this fatal but neglected disease.

This research proposal will be a multicentre, prospective, open label, parallel group, comparative trial to determine the efficacy and safety of sodium stibogluconate (SSG) 20mg/kg/day given for 30 days, Paromomycin (PM) 15mg/kg/day for 21 days, and a combination of SSG and PM, 20mg/kg/day, 15mg/kg/day respectively, given for 17days in the treatment of patients suffering from VL in Ethiopia, Kenya and Sudan. Primary endpoint will be cure rate at 6 months.

LITERATURE REVIEW

The leishmaniases are a group of diseases caused by Leishmania parasites, of which at least 20 different species can cause human disease. Leishmania infection is transmitted by the bite of female sandflies. The disease occurs in three forms: self-healing cutaneous leishmaniasis (CL), mutilating mucosal leishmaniasis (ML or MCL) and life-threatening visceral leishmaniasis (VL). Each form varies in degree of severity, with visceral leishmaniasis being by far the most devastating.

Today, of the estimated 350 million people at risk in 88 countries, 12 million people are thought to be affected by leishmaniasis in its different forms, with an estimated 1.5 -2 million new cases occurring annually (1-1.5 million cases of CL/MCL and 500,000 cases of VL) (WHO 2000). In the past decade, the number of leishmaniasis cases has risen (Desjeux 2001) due to increased human exposure to the sandfly vector as well as the spread of AIDS and other immunosuppressive conditions that have increased the risk of *Leishmania*-infected people developing the disease.

Visceral leishmaniasis (VL) or kala-azar is the most severe form of the disease. If untreated, VL has a mortality rate of almost 100%. In 1999, there were 57,000 (reported) deaths due to kala-azar. Ninety per cent of VL cases occur in five developing countries: India (especially Bihar), Bangladesh, Nepal, North Eastern Brazil, and Sudan.

Distribution of Visceral Leishmaniasis in Eastern Africa

In Eastern Africa, especially Sudan, Ethiopia and Kenya, visceral leishmaniasis is by far the most common form of the disease and is the cause of much death and disease.

VL in Ethiopia has been reported from over 40 localities in different parts of the country. The infection is either due to L. donovani, L. infantum or L. archbaldi. Most infections are acquired in north-west Ethiopia in the lowlands of Metema and Humera, south-west Ethiopia in the Segen, Woitu and Omo river basins, and in other isolated foci in the rift valley. The north-western Metema-Humera focus (which extends northwards to Eritrea and westwards into eastern Sudan) is a major VL focus which presently accounts for approximately 60% of the total disease burden in Ethiopia. This focus extends over a huge land mass in two regions, Region 1 (Tigray) and Region 3 (Amhara). In this focus MSF-H is actively involved in treatment of cases, with at least 2000 cases benefiting from treatment every year. The patients in this focus are mostly migrant laborers, and one would expect up to 40% of the cases to be HIV co-infected. VL foci in Segen, Woitu and Omo river basins represent typical endemicty. The VL cases from these foci, account for approximately 20% of the total burden in the country, and HIV co-infection is less than 2%. These foci are located in the Southern Nations, Nationalities and Peoples Regional Government (SNNPRG). Other foci are in Region 4 (Oromia), Region 5 (Somali), and Region 2 (Afar). Sporadic case reports are known from other smaller localities. For instance, in Moyale, at the borders

with Kenya and in areas northeast of Lake Abaya. Members of the Ethiopian Army and Police Forces who acquire VL in the endemic areas are admitted in Addis Ababa referral hospitals. This is a special risk group and HIV co-infection could be expected to be more than 50%. (Hailu 2004, Ayele 2004)

In Eritrea, the Red Sea littoral (localities like Nakfa, Afabet, Algena, Keren) and the district of Teseney also in Eritrea (North of Humera) are endemic.

Eastern Sudan (Gedarif State), Upper Nile and Western-Upper Nile are known endemic areas for visceral leishmaniasis in the Sudan. VL is among the most important health problems in the Sudan with more than 24,660 cases and 1193 deaths that has been reported during 1996-2001. The number of reported cases is mainly a reflection of reporting rather than the actual disease transmission. Reports and published work from Sudan showed that the disease affects mainly children with few adult cases. The disease is reported to be more prevalent among poor people, malnourished, vagrant, farmers, laborers, water carrier, and those out of country, who have a very limited capacity to assume the costs of the disease (Sudan Manual 2004).

In Kenya, the endemic foci of VL include Baringo, Turkana, West Pokot, Kitui, Meru, and Machakos districts. The first 3 districts are in Rift Valley province while the latter are in Eastern province. Numerous outbreaks of VL were reported from these areas in the late nineteen seventies with over 2000 cases reported from Meru and Kitui districts only (WHO 1990). All these areas are generally semi-arid, sparsely populated with low rainfall and high temperatures. Low agricultural and economic productivity has resulted in poor social economic status (SES) of the population in these areas.

Population displacements as a result of war, drought, famine, or rural-urban migration have exacerbated the spread of the disease. For instance, the epidemic in western Upper Nile, an area where VL was previously not endemic, caused an estimated 100,000 deaths between 1984 and 1992, or a population mortality of up to 36% (Seaman et al. 1996)

Clinical Aspects of Leishmaniasis in Eastern Africa

Visceral leishmaniasis, is a devastating illness, fatal if left untreated. Patients with VL present with fever, malaise, cough, abdominal pain, diarrhoea, epistaxis, splenomegaly, hepatomegaly, cachexia, anaemia, pancytopenia, lymhadenopathy and malnutrition.

Not all infected people develop clinical kala azar; some have a sub clinical infection that spontaneously resolves. The ratio of those with clinical disease to those with sub clinical disease varies remarkably from place to place, and during periods of epidemics. In eastern Sudan, the ratio of clinical cases to mild or sub clinical diseases was 1.6:1, and increased to 3.3:1 during a recent outbreak. The scenario is less critical in Brazil where, during an outbreak, the ratio was 1:8 or 1:16 (showing much less disease per infection); in Iran, it is 1:12 (MSFH 2003)
Malnutrition, anaemia and immune depression increase the likelihood that infection will progress to the disease. In Ethiopia, Kenya, and Sudan the problems of infected children are compounded by these very reasons, as well as opportunistic infections such as tuberculosis and pneumonia. Infected adults also bear the brunt of these problems – in Ethiopia HIV is found in association with kala-azar in approximately 35-50% of cases (Dr Asrat Hailu – personal communication).

The incubation period for VL varies widely and it is estimated to be between 2 – 6 months. Malnutrition, anaemia and immune depression increase the likelihood that infection will progress to the disease.

A complication of visceral leishmaniasis, especially prevalent in Sudan (and to a lesser extent Ethiopia, and Kenya) is post-kala-azar dermal leishmaniasis (PKDL) (Zijlstra et al 2003) occurring in people who have recovered from VL following treatment.

Main treatment options for visceral leishmaniasis.

Treatment of VL cases in Eastern Africa always presents with challenges such as patients coming late when they are extremely ill and may die during treatment due to the illness as well as toxicity of the drugs used. The other challenges include availability of drugs, drug resistance, and cost of treatment (drugs and hospitalization). In VL endemic areas, facilities may not be available for accurate diagnosis and follow up, and the increasing prevalence of HIV co-infection is an additional challenge, particularly in Ethiopia.

Table 1: Current treatment options for patients with visceral leishmaniasis

Drugs available for use	Associated problems
**Pentavalent antimonials**	Toxic, parasite resistance growing<br>30 day IV/IM treatment in hospital
**Amphotericin B**	Used in case of antimonial resistance but dose-limiting toxicity, 15-20 day IV treatment in hospital
**Liposomal Amphotericin B**	Less toxic but prohibitively expensive
**Miltefosine**	Teratogenic, only registered in India, and expensive

Need for new treatment options

In eastern Africa, the first line treatment today in most endemic areas is antimonial therapy for 4 weeks sodium stibugluconate,(Pentostam® from GSK in Kenya or generic SSG from Albert David in Sudan and Ethiopia) used at 20 mg/kg/day for 28-30 days). Although the efficacy of this treatment is not yet compromised by resistance in this region (in contrast to Bihar – India), the painful daily injections, the need for four weeks of hospitalisation, the toxicity when using longer treatments, the low efficacy in HIV co-infected patients and the risk of inevitable drug resistance, as observed in India, make alternative options a necessity. Second line treatments are either toxic or prohibitively expensive.

In 2002-3, a combination of SSG and PM given for 17 days was used in an epidemic situation in Southern Sudan, with an initial cure rate of 97% (personal communication from Koert Ritmeijer, Médecins Sans Frontières). These findings were in line with previous published experience in the same area (Seaman et al, 1993) and experience in Kenya (Chunge et al, 1990). The proposed study aims to confirm these results in a randomized prospective comparative study.

SSG

Despite the shortcomings listed in table 1, sodium stibogluconate (SSG) is still the most widely used drug for VL in Eastern Africa. SSG is known to cause cardiac, muscle, joint and renal problems. Emergence of resistance as has occurred in the Indian subcontinent (Bihar state) make investigating combination schedules a priority.

Paromomycin

Paromomycin (PM) is a broad-spectrum aminoglycoside antibiotic produced from culture filtrates of Streptomyces krestomyceticus and is identical to aminosidine (Shilling & Shaffner, 1961). PM is very poorly absorbed from the gut, an oral formulation is available for the treatment of infections caused by bacteria, protozoa and worms from the intestinal lumen. For the treatment of systemic infections, for example VL, a parenteral formulation is required.
An injectable formulation of 500 mg of PM sulphate has been marketed in several countries for over 35 years for the treatment of bacterial and parasitic infections, however it has not been licensed specifically for the treatment of VL.

The anti-leishmanial activity of injectable paromomycin was first demonstrated in the 1960s and subsequently confirmed in vitro and in vivo. Since then, it has also been shown to be effective against visceral leishmaniasis (Chunge 1990, and others) and is affordable and well tolerated.
Efficacy of PM has also been shown in Bihar, India, the region with the greatest incidence of kala azar and the highest rates of antimony resistance (Thakur 2000)

Historical Product Profile – Farmitalia dossier

Summary data are available on a total of 2,397 patients treated with injectable paromomycin for various infectious diseases. Patient population ranged from newborn infants to the elderly. In most cases, adults received up to 2g/d for 30 days, although patients with skin infections were given up to 1.5g/d for 49 days.

Summary of results:
Paromomycin was well tolerated. Adverse events (AEs) involving hearing function were reported in 10 (0.4%); two patients had renal function decrease and one albuminuria; 21 additional patients had other AEs. The occurrence of AEs was not related to the age of patients. AEs involving hearing tended to occur in patients administered large dose of PM and/or multiple-drug regimens.

Safety Data - Historical Japanese Post Marketing Data:
Pre and post marketing safety surveillance safety data is available from 2220 patients. The incidence of adverse reactions is as follows: Pain at injection site 94 (4.2%), local rash 30 (1.4%), tinnitus 8 (0.4%), malaise 9 (0.4%), skin rash 5 (0.2%), nausea/vomiting 4 (0.2%), diarrhea 2 (0.1%). The major dose limiting toxicities of injectable paromomycin are the same as other drug in the

aminoglycoside class (e.g. streptomycin, gentamycin) being oto- and renal toxicity. These toxicities are related total dose of the drug given and duration of therapy.

Pre-clinical toxicology

Mutagenicity/Genotoxicity: GLP Institute Pasteur Lille

- Mutagenicity test on bacteria using Ames technique
- Genotoxic activity using the micronucleus test
- Mutation assay at the TK locus in L5178Y Mouse lymphoma cells using a microtitre cloning technique
- Test for chromosomal aberrations by in vitro human lymphocyte metaphase analysis

Results: All tests were negative for mutagenicity/genotoxicity

Animal toxicology

Table 2: Animal toxicology studies – part 1

Study	Species	Route	Dose/Duration	Sponsor	GLP	Main results
Acute	Mouse, Rat	IV, IM, IP, IC, SC, PO	Mice- LD_{50} g/kg<br>- IV 0.106- 0.110<br>- IP 0.750<br>- SC 0.70 - 1.06<br>- IC 0.023<br>- PO 15.0 –17.8<br>Rat – LD_{50} g/kg<br>- IM 1.20<br>- SC 0.87<br>- PO 21.62	FCE	No	The LD_{50} is 8-10x greater than the therapeutic dose in humans
Chronic	Mice, Rats, Cats	IM	Mice mg/kg/day<br>- 400 for 60 days<br>Rats mg/kg/day<br>- 264 for 82 days<br>Cats mg/kg/day<br>- 50 for 37 days	FCE	No	No mortality or vestibular damage seen
Nephrotoxicity	Mice, Rats, Cats	IM	Mice mg/kg/day<br>- 400 for 60 days<br>Rats mg/kg/day<br>- 264 for 82 days<br>Cats mg/kg/day<br>- 50 for 37 days	FCE	No	Mice moderate renal damage<br>Rats slight renal damage<br>Cats moderate renal damage

Table 3: Animal toxicology studies – part 2

Study	Species	Route	Dose/Duration	Sponsor	GLP	Main results
Cochleo Vestibular	Rats, Guinea Pigs,	SC	Rats mg/kg/day<br>- 200, 264 for 60 days<br>G. Pigs mg/kg/day<br>- 50, 100, 200, 400 for 30 days<br>- 20 for 60 days<br>- 200 for 28 days comparative trial with KM and DHSM	FCE	No	Rat dose related cumulative effect on acoustic sensitivity<br>Guinea Pig dose related cumulative effect for oto-toxicity. In the comparative trial AM was less ototoxic than KM or DHSM
Reprotox	Mice, Rats, Rabbits	IM, SC	Teratogenesis<br>Mice mg/kg/day<br>- 100, 200, 300 IM for 7 days<br>Rats mg/kg/day<br>- 100, 200, 300 IM for 7 days<br>Embryo-fetal<br>Rats mg/kg/day<br>- 100, 200 SC for 19 days<br>Rabbits mg/kg/day<br>- 12.5, 25 SC for 28 days	FCE	No	No teratogenic effect detected.<br>No statistically significant embryo-fetal toxicity
Thirteen Week Chronic Toxicity	Dogs	IM	Dogs mg/kg/day<br>30, 100 for 13 weeks	SoloPak	Yes	Low dose dogs slight to minimal renal damage, and a frequency dependent hearing loss at high tones. High doses dogs severe chronic nephropathy, and renal tubular degeneration. Unable to detect audiometric hearing frequencies Swelling and chronic inflammation at injection site

Clinical Pharmacology

Pharmacokinetics

An HPLC assay was developed at the University of Illinois at Chicago under GLP conditions in order to be able to determine the concentration of paromomycin in biological fluids (e.g. urine and plasma)
Single Dose Intramuscular Pharmacokinetics in Healthy Normal Volunteers
Sixteen HNVs were given a single IM dose of paromomycin base either 12 or 15 mg /kg (8 per group)

Table 4: Pharmacokinetic parameters

Dose mg/kg /day	C_{max} (µg/ml)	T_{max} (h)	K_a (h^{-1})	T_{lag} (h)	AUC (µg h/ml)	CL/F (ml/min/1.73M^2)	V_β/F (l/kg)	$t_{1/2}$ (h)
12	21.6	1.19	6.27	0.23	86.3	117.7	0.35	2.21
15	23.4	1.51	2.65	0.20	104.5	126.0	0.41	2.64

Dose finding Studies

1) Randomized phase II clinical study: Kala-azar Research Centre, Muzaffarpur, Bihar, India; T.K. Jha (Jha et al., 1998)

Group (mg/kg/d)	Enrolled	Treatment Completed	Treatment Failures	Relapses	Defaulters	Definitive Cure 180d (%)
PM 12 x 21d	30	30	2	5	0	23/30 (76.7)
PM 16 x 21d	30	30	0	1	1	28/29 (96.5)
PM 20 x 21d	30	30	0	1	0	29/30 (96.7)
SB 20 x 28d	30	30	8	3	0	19/30 (63.3)

2) Randomized phase II clinical study: Patna Medical College, Patna, Bihar, India; (Thakur *et al*, 2000: 94:)

Group (mg/kg/d)	Enrolled	Treatment Completed	Treatment Failures	Relapses	Defaulters	Definitive Cure 180d (%)
PM 12 x 21d	30	30	0	3	0	27/30 (90.0)
PM 16 x 21d	30	30	0	3	3	24/27 (88.9)
PM 20 x 21d	30	30	0	4	1	25/29 (86.2)
SB 20 x 28d	30	30	8	1	1	20/29 (69.0)

3) Randomized, comparative, open-label trial of the safety and efficacy of Paromomycin (PM) + sodium stibogluconate (SB) versus sodium stibogluconate alone for the treatment of visceral leishmaniasis: Patna Medical College, Patna, Bihar, India; (Thakur *et al*, 2000)

Group (mg/kg/d)	Enrolled	Treatment Completed	Treatment Failures	Relapses	Defaulters	Definitive Cure 180d (%)
PM12+SBx 21d	52	51	3	1	0	48/52 (92.3)
PM18+SBx 21d	48	46	2	1	0	45/48 (93.8)
SB20 x 28d	50	46	21	1	1	27/50 (54.0)

Clinical Experience with injectable paromomycin in the Treatment of VL

Previously, clinical trials with injectable PM either alone or in combination with SB for the treatment of VL have been conducted in Africa (Kenya and Sudan), India (Bihar), and in cases imported into the United Kingdom (Jha, et al., 1998, Hassan M, et al., 1995 Thakur et al., 1995; Seaman et al, 1993, Thakur et al, 1992, Scott et al, 1992, Chunge et al., 1990). In all the studies the investigators reported that PM, used as a single agent or combined with SB was highly efficacious and well tolerated in the treatment of VL caused by L. donovani or infantum.

Table 5: Summary of clinical studies using PM

Dose mg/kg/day	Single Agent No. Patients	Place	Combination Therapy with SSG No. Patients	Place
6			40	India (Thakur)
12	60	India (30 Jha, 30 Thakur)	120	India (96 Thakur, 24 Thakur)
14-16	19	Kenya (Chunge)	124	Kenya and Sudan (23 Chunge, 101 Seaman)
16	60	India (30 Jha, 30 Thakur)		
18			50	India (Thakur)
20	60	India (30 Jha, 30 Thakur)		
**Total Patients**	**199**		**384**	

TRIAL OBJECTIVES AND PURPOSE

Currently in the three countries, Sudan, Kenya and Ethiopia many of the patients present themselves in remote areas and need to be treated in relative resource poor settings. It is for this reason that standardised treatment with proven efficacy is much needed. A shorter course of treatment is not only advantageous for the patient but also reduces the overall case load in the clinics thus reducing the risk of disease outbreaks in already immuno-compromised kala-azar patients. Paromomycin, either alone or in combination with SSG would decrease the treatment duration substantially. An additional added value of combination therapy is that it is likely to reduce the chances of development of parasite resistance against the individual drugs.

Leishmaniasis experts in the three countries are in agreement that there are potential benefits of the combination treatment of SSG and PM and that its efficacy should be evaluated with the view to introduce this protocol if proven efficacious and safe. There is ample circumstantial evidence of the use of this combination therapy and its efficacy and tolerability as a standardized protocol. This can only be confirmed through a randomised controlled study with 6 months follow up.

HYPOTHESIS

That a combination course of SSG and PM (17 days) is similar in efficacy to either PM alone(21 days) or SSG alone (30 days) and that the shorter course combination (17 days) is not more toxic than PM or SSG alone.

OBJECTIVES OF THE TRIAL

1) To assess the efficacy and safety of SSG 30 days alone in the treatment of patients with VL.

2) To assess the efficacy and safety of PM 21 days alone in the treatment of patients with VL.

3) To assess the efficacy and safety of SSG and PM as a combination course of 17 days in the treatment of patients with VL.

METHODOLOGY

STUDY DESIGN

This will be a multi-centre, prospective, open, parallel group, comparative trial of efficacy and safety of SSG alone given IM/IV (according to usual hospital practice) for 30 days versus paromomycin alone given IM for 21 days versus a combination of SSG and PM given for 17 days, in the treatment of patients suffering from VL in Ethiopia, Kenya and Sudan.
Patients who have clinical symptoms and a confirmed parasitological diagnosis of VL by splenic aspirate, lymph nodes aspirate or bone marrow aspirate (to be specified for each hospital site) and who have fulfilled the inclusion/exclusion criteria will be enrolled.
The primary endpoint will be cure rate at 6 months post treatment. Secondary endpoints will be cure rate at end of treatment (Day 31 for SSG, Day 22 for PM, Day 18 for PM + SSG) and at three months post treatment.

STUDY SITES

The study will be conducted at the following sites;

Ethiopia:
- Arba Minch hospital
- Gondar hospital

Kenya:
- Centre for Clinical Research (CCR), Kenya Medical Research Institute (KEMRI), Nairobi.

Sudan:
- Kassab Hospital
- Um-El-Kher (MSFH treatment centre)

INCLUSION CRITERIA

Patients who fulfill the following inclusion criteria will be enrolled into the study:-

1) Patients for whom written informed consent has been signed by the patients themselves (if aged 18 years and over) or by parents(s) or legal guardian for patients under 18 years of age.

2) Patients aged between 4 and 60 years (inclusive) who are able to comply with the protocol. It is justified to include children because they represent more than 50% of VL cases.
3) Patients with clinical signs and symptoms of VL and diagnosis confirmed by visualization of parasites in tissue samples (spleen, lymph node or bone marrow) on microscopy.

EXCLUSION CRITERIA

Patients with the following will be excluded from the study:

1) Patients who have received any anti-leishmanial drug in the last 6 months.
2) Patients with a negative splenic / lymph node / bone marrow smears.
3) Patients with a clinical contraindication to splenic/lymph node/ bone marrow aspirates.
4) Patients with severe protein and or caloric malnutrition (Kwashiokor or marasmus)
5) Patients with previous hypersensitivity reaction to SSG or aminoglycosides.
6) Patients suffering from a concomitant severe infection such as TB or any other serious underlying disease (cardiac, renal, hepatic) which would preclude evaluation of the patients response to study medication.
7) Patients suffering from other conditions associated with splenomegaly such as schistosomiasis.
8) Patients with previous history of cardiac arrhythmia or an abnormal ECG
9) Patients who are pregnant or lactating.
10) Patients with haemoglobin < 5gm/dl.
11) Patients with WBC < 1 x $10^3/mm^3$.
12) Patients with platelets < $40,000/mm^3$.
13) Patients with liver function tests more than three times the normal range
14) Patients with serum creatinine outside the normal range for age and gender
15) Patients with pre-existing clinical hearing loss.

<u>NB</u>
Relevant tests will be done to exclude the above listed conditions.

HIV-status and VCT

All patients will be offered counseling and screening for HIV (voluntary counseling and testing programme (VCT). This may either be done at the same

time as consent is obtained for inclusion in the trial or at a later date according to hospital practice. HIV positive patients will not be excluded from the clinical trial. Subset analysis will be performed to assess any differences in response.

CRITERIA FOR PATIENT WITHDRAWAL

Patients will be considered to have completed the study if they satisfy all entry criteria, complete the course of treatment and attend the 6 month follow-up visit.

Patients will be considered to have withdrawn from the study if they had entered into the study (i.e. gave informed consent and received at least one day's treatment) but did not complete the treatment period and follow up period.

Treatment failure will be defined as no change or an increase in the patient's disease severity i.e. in signs and symptoms of VL, and parasitology, such that the patient is withdrawn from the study and alternative therapy given.

A patient may be withdrawn from the study at any stage if the investigator or the DSMB considers there is a serious risk to the patient from continuation in the protocol. Alternative therapy will be provided to the patient if needed, upon withdrawal from the study.

A patient may withdraw, or be withdrawn, from the study for one of the following reasons:

- Serious adverse events (drug related or not)
- Deviation from protocol (including non-compliance)
- Lost to follow-up
- Termination by the sponsor
- Withdrawal of consent

The reason for termination will be recorded on the CRF. Patients withdrawn from the study will be followed-up at 3 and 6 months for monitoring of adverse events wherever possible. Every effort will be made to follow up withdrawn patients in order to determine the final outcome. This information will be recorded in the CRF and these patients data will be analysed as those who failed to respond to treatment.

Randomization in the Clinical Trial (RCT)

The multi-country study adopted restricted randomization in a three-arm study per country. This approach prevents the potential pitfall/imbalance in study numbers that could have resulted if one used simple random sampling. In order to avoid manipulation of blocks of small sizes, blocks of size 15 will be used in randomization. This approach ensures that the study balances after the 15th patient. In the allocation of the drugs to the patients concealment will be used in order to minimize selection bias. Opaque envelopes will be numbered sequentially and then sealed. This process will be carried out at the coordinating centre at KEMRI.

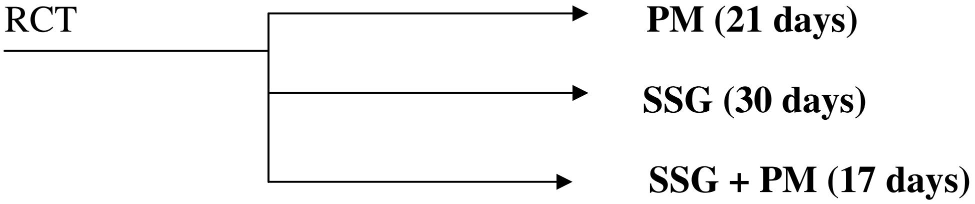

Fig 1: The structure of the RCT

Sample size Determination

The sample size will be used to test the following statistical hypothesis as stipulated earlier:

Hypothesis: The three therapies differ by clinically relevant amounts
Alternative Hypothesis: The three therapies do not differ by clinically relevant amounts

A cure rate of 85% to 95% was used in the sample size calculation, where 85% represents the worst-case scenario and 95% the best outcome. The difference of these two proportions gives the size of the treatment effect sought in the randomized clinical trials.
This means that we are dealing with a dichotomous variable where the patients will be categorized as cured or not cured. A power of 90% and 5% level of significance are also required. The calculated sample size was adjusted for attrition and other covariates such as HIV/AIDS which was observed to be high in some of the participating countries.

Allocation adopted – uniform and equal allocation where $\lambda=n_t/n_c=1$
The sample size $n=r*n_t$ [r is the number of active groups] which is calculated as follows:-

n_t **[per arm]=k* [(p_1(1-p_1) + p_2(1-p_2))/(p_1-p_2)^2]**

Where:
n_t = sample size
$z_{a/2}$ = the corresponding value to the 95% CI
z_β = The corresponding value to power of 90%
p_1 = anticipated cure rate = 95%
p_2= worst case scenario = 85%
$q_1 = 1- p_1$
$q_2 = 1- p_2$
where k= 10.5 for power of 90%

<u>For 90%</u>

n_t >= 10.5 [(0.85(0.15) + 0.95(0.05))/0.01 = 184

The sample size was adjusted to account for attrition rate due to loss to follow up, which is anticipated to be 15%. Thus the minimum sample size becomes n = 217.

HIV/AIDS was likely to be a covariate that could affect the primary variable-cure rate, especially in Ethiopia where the co-infection rate is estimated to be above 20%. This necessitated a further adjustment of the sample size for Ethiopia by 20% in order to be able to attribute the true treatment effect of the test drugs after controlling for HIV/AIDS. Thus the total for the three arms is 695.

The participating teams from Kenya, Ethiopia, Sudan and MSF were asked to specify the number of cases they could conveniently handle given the existing capacity and available infrastructure. This approach was used in absence of any other criteria. On the other hand some of the countries have very high case-load.

Table 1: Sample size per group

Teams	Expected Sample per Group(rounded to complete block of 15)
Kenya	60
Sudan MSF	150
Sudan MOH	225
Ethiopia – Gondar Province	150
Ethiopia-Southern Province	120
Total	705

For further reading on sample size estimation consult (Chan 2003)

TREATMENT

Eligible patients for whom informed consent has been obtained will be randomized to either of the three treatment regimens using a computer generated randomization code provided.

Drug Administration:

SSG will be given IM or IV at a dosage of 20mg/kg/day* for 30 days.
PM will be given IM at a dosage of 15 mg/kg/day for 21 days.
SSG + PM combination: SSG will be given IM or IV at a dosage of 20mg/kg/day* for 17 days and PM at a dosage of 15 mg/kg/day IV/IM for 17days
***The maximum dosage of SSG per day for any patient is 850mg (8.5ml)**

Treatment will be given by the clinicians/nurse at the same time each day and a treatment sheet indicating time of dosing bearing the signature of the attending clinician/nurse will be kept.

Rescue medication

In the event of failure to respond to treatment, clinical deterioration or relapse at any time during the study, rescue treatment consisting of IV Ambisome® (a liposomal formulation of amphotericin B) at a dosage of 3 mg/kg/day for 5 days or according to local Ambisome® rescue protocol, the exact regimen used at each trial site to be documented in the case report form..

Prior and Concomitant Medications

No additional anti-leishmanial therapy will be permitted during the course of the study. If such therapy becomes necessary, the patient will be withdrawn from the study and considered a treatment failure.

Concomitant medication necessary for the health of the patient will be permitted during the course of the study. This will include the concomitant use of drugs such as paracetamol as an analgesic/antipyretic. Details of all concomitant medication taken during the study will be recorded in the CRF with indication, daily dose, route and dates of administration.

In the case of a patient presenting with co-infection, eg. pneumonia or malaria, these infections should be treated first. The patient should be re-assessed for suitability for inclusion in the trial after one week.

EFFICACY ASSESSMENT

Efficacy will be assessed using clinical, haematological, biochemical and parasitological responses.

- Clinical Assessment

The clinical evaluation will involve measuring the spleen size by palpation below the left coastal margin, temperature, blood pressure, body weight on days 0, 7, 14, 21 and end of treatment (18, 22, 31), 3 months and 6 months post treatment.
ECG and audiometry will be done at baseline, day 14, end of treatment and 6 months follow-up, in selected sites.

- Haematological and biochemical assessment

Blood will be analyzed for haemoglobin, WBC, platelets, urea, creatinine, and liver function tests on days 0, 7, 14, 21 and end of treatment (18, 22, 31), 3 months 6 months post-treatment.

- Uninalysis

Urinalysis will be performed on days 0, 7, 14, 21 and end of treatment (18,22,31) 3 months and 6 months post-treatment.

- Parasitological assessment

Parasitological assessment involves aspirating the spleen, lymph node or bone marrow at baseline, end of treatment (18, 22, 31 days depending on treatment arm) on, and at 3 months and 6 months follow up visits for all study patients. In addition, at selected sites aspirates will be cultured. Each patient will have a maximum of four aspirates. Patients who are clinically well with no signs or symptoms of VL and no palpable lymph nodes / spleen at the three months visit do not to have any aspirate at this visit. All patients will have an aspirate at 6 months post treatment.

The source of parasitological specimens should remain unchanged throughout the treatment and follow up periods, unless the spleen / lymph node initially chosen as the source of parasitological specimens becomes unpalpable, in which bone marrow aspirate should be performed.

Since bone marrow, lymph node and splenic aspirates are invasive procedures, these should only be performed at other times if clinically indicated.

PRIMARY EFFICACY ENDPOINT

The primary efficacy variable is parasitological clearance at 6 months post treatment by splenic, lymph node, or bone marrow smear.

SECONDARY EFFICACY ENDPOINT

The secondary efficacy endpoint will be parasitological clearance at the end of treatment (18, 22, 31 days depending on treatment arm – Test of cure (TOC)) and at 3 months post treatment.

SAFETY ASSESSMENTS

During treatment and at follow up, safety will be assessed by means of haematological, urinalysis and biochemical monitoring as above, and by ECG and audiometry at selected sites. In addition, patients will be asked at each visit if they have suffered any side-effects or other unexpected adverse events.

Adverse events

An adverse event will be defined as any noxious, pathological or unintended change in anatomical, physiological or metabolic functions as indicated by physical signs, symptoms and/or laboratory changes occurring in any phase of the clinical study, whether or not they are considered to be associated with the study drug. This includes an exacerbation of pre-existing conditions or events, intercurrent illnesses, drug interaction, or the significant worsening of the disease under investigation that is not recorded elsewhere in the CRF under specific efficacy assessments. Anticipated day to day fluctuations of pre-existing conditions, including the disease under study, that does not represent a clinically significant exacerbation or worsening of the condition, will not to be considered adverse events.

All adverse events occurring after the start of the study (defined as when informed consent was obtained) are to be reported. This is regardless of whether or not they are considered to be drug related. Adverse event (AEs) elicited by the investigator asking the patient or the patient's parent or guardian a non-leading question such as "Do you/has your child felt different in any way since starting the new treatment/the last assessment?" If the response was "Yes", the nature of the event, the date and time (where appropriate) of onset, the duration, maximum intensity (see below) and relationship to treatment are to be established (see below). Details of any changes to the dosage schedule or any corrective treatment are to be recorded on the appropriate pages of the CRF.

Assessment of Intensity/Severity

The assessment of intensity/severity will be based on the investigator's clinical judgment. Maximum intensity/severity will be assigned to one of the following categories.

Mild: An adverse event, which is easily tolerated by the patient, causing minimal discomfort and not interfering with every day activities.

Moderate: An adverse event, which is sufficiently discomforting to interfere with normal everyday activities.

Severe: An adverse event, which prevents normal everyday activities.

Assessment of Causality

The investigator will use clinical judgment to determine the degree of certainty with which adverse event is attributed to drug treatment. Alternative causes, such as natural history of the underlying diseases, concomitant therapy, etc are to be considered taking into account the known pharmacology of the drug, any previous reactions, literature reports and relationship to time of drug ingestion or recurrence on re challenge. Causality will be assessed using the following categories; not related, unlikely, suspected (reasonable possibility) or probable. Patients with adverse events will be followed-up until the event disappears or the condition stabilizes.

Serious Adverse Events

A serious adverse event will be defined as any event which is fatal, life threatening, disabling or incapacitating or results in hospitalization, prolonged hospital stay or is associated with congenital abnormality, cancer or overdose (either accidental or intentional). In addition, any experience which the investigator regards as serious or which suggests any significant hazard, contraindication, side effect or precaution that might be associated with the use of the drug will be reported as a serious event. Any serious adverse event occurring either during the study or within 30 days, or 5 half lives (whichever is longer), of receiving the last dose of study medication, is to be reported by telephone to the study monitor within 24 hours. This will be followed by a full written summary containing relevant hospital case records and autopsy reports where applicable.

As treatment is by parenteral injection, over dosage is not anticipated. However, in the event of over dosage (error of dosage calculation or administration) will be communicated to the study coordinator, Dr. Monique Wasunna, within 24 hours or as soon as possible thereafter. Details of any signs or symptoms and their management will be recorded in the CRF including details of any antidote(s)

administered. As there are no specific antidotes available for the medications to be used in this study, patients will receive all supportive care needed at discretion of the treating physician and after consultation with the study coordinator above.

DATA COLLECTION, STORAGE AND ANALYSIS

Data Management

In order to ensure data quality, a uniform hard copy i.e case report form (CRF) will be designed for use at all the sites. Data will then be sent to the coordinating sitefor data entry. It will be the responsibility of the investigator to ensure that the CRF is correctly completed to avoid unnecessary delays.

The software of choice will be EpiInfo 2003 which has an adequate electronic data capture (EDC) module especially for double entry. The data will be entered using pre-designed screens matching the data collection tool for ease of entry and validation. The entry program will also have in-built checks to minimize entry errors such as minimum and maximum, allowable values, legal values, jumps and values one must fill.

This exercise will be carried out by well-trained data entry personnel who will manage the data under the guidance of the biostatistician at the Centre for Clinical Research Centre of KEMRI.

Analysis

In the analytical approach intention to treat will be used. The aim is to estimate the difference in treatment outcomes for the three arms. The intention to treat analytical method will take care of events such as patent withdrawal from the trial, failure to comply with treatment, change in treatment and lost to follow-up.

There are two basic options:

Analyse final outcome only for those who complied perfectly with each treatment

Analyse data for all subjects in the groups to which they were randomized. This is referred to as 'Intention to treat' analysis or the 'pragmatic approach'.

The second option will be adopted, analysis will be carried out according to the original treatment assignment regardless of adherence to treatment or protocol.

There will be no exclusion of patients or events. Drop-outs will be checked to establish whether they were systematic or non-random. The analysis will, as much as possible stick to the protocol to avoid data dredging as part of post ad hoc analysis.

Note: Specific country analysis will be of little value because it lacks power. The data will have to be pooled for it to achieve the necessary power in the efficacy analysis.

Summary statistical measures to be computed

The summary statistics will include the χ^2 and risk ratios per arm for the overall efficacy comparison. The analysis and interpretation will heavily rely on confidence intervals of the cure rates among the three arms of the RCT. Other estimations to be made will be the calculation of the power finally achieved by the trial. This will be followed by the presentation of the results in terms of basic descriptive statistics and statistical diagrams. Finally multivariate analysis will be carried out to establish the socio-demographic factors associated with the main parameters.

QUALITY CONTROL AND QUALITY ASSURANCE

All study sites and data generated during the study will be regularly monitored by GCP trained clinical monitors. Wherever possible, CRF data will be verified against hospital source data, for example patient notes or laboratory reports, etc.

ETHICAL CONSIDERATIONS

The study protocol together with patient information and consent forms will be submitted to the local scientific and ethics committee and any other regional or national regulatory authorities as required in the three countries, Kenya, Sudan and Ethiopia, before the study starts and any patient receives study medication.

The patients who participate in this study will be hospitalized and under close monitoring. The invasive diagnostic methods used in the study are those used in

normal clinical practice when treating patients with VL. However, the frequency of testing might be increased depending on the patient's response to treatment.

Children will be included in this study because they represent more than 50% of VL cases in this region.

The effective treatment of VL benefits not only the individual patient but also the community by reducing the reservoir of infection for onward transmission by the sandfly vector. The evaluation of new and better treatments for VL, including shorter courses and combinations is anticipated to have a positive effect on development of parasite resistance and will reduce hospitalisation costs. If paromomycin is found to be efficacious and safe, it will be registered for the treatment of VL, providing a new alternative to treatments already available.

Patients will experience some pain while blood is drawn during venepuncture. The amount of blood to be drawn will be 10 mls before treatment and 7 mls at each subsequent visit, with a total of 42 mls (PM alone and a combination of PM and SSG), 49 mls (SSG alone) over the 6 months study period.

SSG has been extensively used in Sudan, Ethiopia and Kenya. Known adverse events include cardiac, muscle, joint and renal toxicity. PM has been used in clinical trials in Sudan and Kenya during the 1990's, and more recently in humanitarian emergency setting in Sudan. Known adverse events of the aminoglycosides include ototoxicity and renal toxicity.

Patients who are found to be HIV positive will be offered anti retroviral treatment at no cost in accordance with national guidelines for treatment.

A Data Safety and Monitoring Board (DSMB) will be set up to regularly review safety data.

Patients will be reimbursed for travel to and from the study site and will not receive any payment for trial participation. Any medication that is required during the trial period will be provided free of charge to the patient.

INSURANCE AND LIABILITY

DNDi is insured to indemnify the collaborating investigator for any injury or harm which occurs during the performance of the trial according to the protocol signed

by the investigator. Furthermore, DNDi will in accordance with the declaration of Helsinki on Ethical principles for medical research involving human subjects, make all reasonable efforts to protect patients from any harm which may occur during the trial, and will wherever possible ensure that any patient that does suffer harm will receive the best possible treatment available in that country to alleviate their suffering.

TIME FRAME

The study is expected to start in October 2004 and will last 12 to 18 months (6-12 months recruitment period plus 6 months follow up)

REFERENCES

Ayele T and Ali A (2004) The distribution of visceral leishmaniasis in Ethiopia. Am. J. Trop Med Hyg., 33, 4, 548 - 552.

Chan Y.H. (2003). Randomised Contolled Trials (RCTs)- Sample size: The magic number? Singapore Med J 44(4): 172-174

Chunge C.N, Owate J, Pamba H. and Donno H.O. (1990). Treatment of visceral Leishmaniasis in Kenya by aminosidine alone or combined with sodium stibogluconate. Trans. Roy. Soc. Trop. Med. & Hyg. 84: 221-225

Desjeux P. 2001 The increase in risk factors for leishmaniasis world wide. *Trans R Soc Trop Med Hyg*. **95**:239-43

Hailu A., Gebre-Michael T., Berhe N and Balkew M. (2004). Leishmaniasis in Ethiopia. In: *The Ecology and Epidemiology of Health and Disease in Ethiopia*. New Edition; Eds, H. Kloos, Berhane Y and Hailemariam D. (in press)

Hassan M, Baat D.B and Hassan K.A (1995).New breakthrough in treatment of visceral leishmaniasis in children.
Journal of the Pakistan Medical Assocaition 45: 155 – 157

Kanyok T.P. Killian A.D., Rodvold K. A and Danziger L.H (1997).
Pharmacokinetics of intramuscularly administered Aminosidine in Healthy Subjects.
Antimicrobial Agents and Chemotherapy 41:982-986

Manual For The Diagnosis and Treatment of Leishmaniasis, Book, PP: 5, (2004) By *Experts in leishmaniasis in Sudan.)*
MSFH Kala azar manual, version March 2003

Schilling R. T and Schaffner C.P. (1961). Differentiation of catenulin-neomycin antibiotics: Identity of catenulin, paromomycin, hydroxymycin and aminosidin.

Antimicrobial Agents and Chemotherapy 4: 275 – 285

Scott J.A.G., Davidson R.N., Moody A.H, Grant H.R., Felmingham D, Scott G.M.S., Olliaro P and Bryceson A.D.M. (1992) .Aminosidine (Paromomycin) in the treatment of leishmaniasis imported into the United Kingdom. Trans. Roy. Soc. Trop. Med. & Hygiene 86: 617-619

Seaman J, Pryce D, Sondorp H.E, Moody A, Bryceson A.D.M. and Davidson R.N. 1993). Epidemic Visceral Leishmaniasis in Sudan: Randomised Trial of aminosidine plus sodium stibogluconate versus sodium stibogluconate alone.
The Journal of Infectious Diseases 168:715 – 20.

Seaman J. Mercer AJ, Sondrop E. (1996) The epidemic of visceral leishmaniasis in western Upper Nile, Southern Sudan: course and impact from 1984 t0 1994.
Int J. Epidemiol, **25**:862-71.

Thakur C.P, Bhowmick S, Dolfi L and Olliaro P. (1995). Aminosidine plus Sodium stibogluconate for the treatment of Indian Kala-azar: a randomized dose-finding clinical trial.
Trans. Roy. Soc. Trop. Med & Hyg 89: 219-233

Thakur C.P., Kanyok T.P., Pandey A.K., Sinha G.P., Zaniewski A.E, Houlihan H.H and Olliaro P. (2000). A prospective randomized, comparative, open label trial of the safety and efficacy of paromomycin (aminosidine) plus Sodium Stibogluconate versus Sodium stibogluconate alone for the treatment of visceral leishmanisis.
Trans.Roy.Sox.Trop.Med & Hygy 94: 429 -431

Thakur C.P., Olliaro P, Gothos Kar S, Bhowmich S, Choudlhury B.K., Prasad S, Kumar M, Verma B.B. (1992) Treatment of Visceral leishmaniasis (Kala-Azar) with Aminosidine (paromomycin) antimonial combinations, a pilot study in Bihar, India.
Trans. Roy. Soc. Trop. Med & Hyg. 386: 615 – 616.

Thakur C.P., Sinha G.P., Pandey A.K, Kumar N, Kumar P, Hassan, Narain S.M. and Roy R. (1998). Do the diminishing efficacy and increasing toxicity of Sodium Stibogluconate in the treatment of Visceral leishmaniasis in

Bihar, India, justify its continued use as a first line drug? An observational study of 80 cases.
Annals of Tropical Medicine and Parasitology 92:561-569

WHO Expert Committee Report (1990). Control of the leishmaniasis. *WHO Tech Rep Ser* 993

WHO Fact Sheet, revised May 2000

Zijlstra E.E, Musa A.M, Khalil EAG, EI Hassan IM, EI- HssanAM(2003). Post Kala-Azar Dermal Leishmaniasis.
Lancet Infect Dis, 3:87-98.

PATIENT INFORMATION AND CONSENT

TITLE: A MULTICENTRE COMPARATIVE TRIAL OF EFFICACY AND SAFETY OF SODIUM STIBOGLUCONATE (SSG) VERSUS PAROMOMYCIN (PM) VERSUS COMBINATION OF SSG AND PM AS THE FIRST LINE TREATMENT FOR VISCERAL LEISHMANIASIS IN ETHIOPIA, KENYA AND SUDAN

PRINCIPAL INVESTIGATOR(S):

	**ETHIOPIA**	**Dr. Asrat Hailu**
	**KENYA**	**Dr. K.M. Wasunna**
	**SUDAN**	**Dr. Musa Amudawi**
	**SUDAN**	**Dr. Getahun Mengistu**
	**MSFH**	**Dr. Manica Balasegaram**

SPONSOR: Drugs for Neglected Diseases Initiative, Geneva, Switzerland

Introduction

We are studying kala-azar disease, which is common in our countries. The test you have had performed indicates that you have been infected by a parasite called *Leishmania* that causes the illness kala-azar. We are studying new drug treatments for this disease, and would like you to participate in this trial to test a new drug called paromomycin. We wish to compare it with the usual treatment called SSG, and a combination of both drugs used together.

Paromomycin alone and the combination with SSG have been shown to be useful in small studies in some countries but we do not have sufficient evidence of safety and efficacy to enable us to get paromomycin registered in this country.

The study is expected to start in 2004 and will last for 12-18 months. A total of 705 patients will participate.

We would like you to be included in this number, but your participation is voluntary.

Procedures during the trial

Because we do not know which treatment is most effective, you will be allocated to one of the three treatment choices by a process called randomization, which means that the chances of you getting any of the three treatments is the same.

You will be admitted to the ward for the duration of the treatment. This is given as a daily injection into a vein or muscle for up to 30 days, depending on which treatment choice you are allocated.

Known side effects of these drugs include, pain at the injection site, skin rash, nausea and vomiting, diarrhea, feeling tired, ringing in the ears and rarely damage to the heart, kidneys or hearing. During the trial we will regularly assess your progress by means of blood tests, urine tests, heart tracings and hearing tests. A total of 10 mls of blood (two tea spoons) will be drawn at the beginning of the trial and 7 mls (one and a half tea spoons) at each subsequent weekly assessment during treatment and at follow up. We shall also need to repeat the test on your lymph nodes/spleen/bone marrow to make sure that the drugs are killing the kala- azar parasites. These tests are necessary but not without risk.

Occasionally splenic aspiration may result in internal bleeding. This is very unlikely but may occur as a complication in approximately 1 in 1,000 patients. The risks can be minimized in a number of ways. For instance, we will determine any bleeding problem you may have by a blood test. If this test indicates you are at risk of bleeding, we will use lymph node (LN) aspiration or bone marrow (BM) aspiration instead. If it is necessary to do a bone marrow test we will perform it under local anesthesia, to reduce the pain of this procedure. Special precautions will be taken in cases of children. The child must be calm and still during the procedure, so will be held gently onto the bed by a nurse to avoid movement.

Local anaesthetic and mild sedation will be given. In case bleeding occurs we will look after you until you are fully recovered.

On rare occasions there might be failure of treatment using the study drugs. In this case, you will receive treatment with another drug called liposomal amphotericin-B (AmBisome). This drug is known to be very effective and safe. It is not available in Ethiopia, Kenya and Sudan because it is very expensive, however, we shall make this drug available to you, at no cost, to make sure we can cure your disease.

We need to assess the long term effects of the drugs and therefore we shall need you to attend two follow up appointments at 3 months and 6 months after your treatment has finished. For school children, this will mean absence from school on those days.

Benefits

The main benefit of participation in the study is that you will be cured of the disease called kala azar. If the study is successful it means that an alternative shorter treatment will be available for this disease which will benefit your community and may reduce the likelihood of other people getting the disease.

Confidentiality

At the end of the study, we plan to write a report about the results of the study. The reports will not bear any information relating to you personally e.g. your name or identity. We assure you of the confidentiality of such information. Thus, we also need your permission to use the test results for writing a report.

In addition, clinical monitors of the sponsor (DNDi) or the regulatory authorities may wish to inspect your records.

Right to refuse or withdraw

You do not have to take part in this research, your participation is voluntary. If you do not wish to do so and this will not affect your treatment at this centre in any way. You will still have the benefit of treatment for your disease at this centre.
If you do decide to participate and then change your mind later, you may do so, at any time, without losing any of your rights as a patient.

It is also possible that we may decide to withdraw you from the study if we believe it is in your best interests, in which case you will continue to receive the usual treatment for kala-azar until you are better..

The sponsor (DNDi) may also decide to terminate the study. In this event we will continue to treat you until you are better.

In the event that you suffer an injury or illness related to participating in this trial, DNDi will pay all costs relating to treatment of the injury or illness.

You will not receive any money for your participation in the study, however, we will pay your travel expenses to attend the hospital for treatment and hospital follow up visits at 3 and 6 months. In the rare event that you suffer complications due to study treatment, we will do everything possible to ensure you receive the necessary medical care and treatment for this complication.
If you agree to participate in the study, we will ask you to read and sign the consent form.
Do you have any questions?

Patient information for HIV testing

As we have explained to you, you have kala-azar infection and we are treating you with one of the three trial drug treatments. We are now asking you to be tested for another infection. It is a test for HIV infection. We have very important reasons to test you for HIV, which we would like you to understand.

If you are HIV positive you may not respond to treatment and we may need to give you additional treatment. In case you are HIV positive, it will be beneficial for you to know, both for your own well being, and also for your family, friends and other persons living with you.

We advise you to consider being tested. If you wish to be tested, a counselor will hold confidential discussions with you before and after the test. We will inform you of the test results. If you are HIV positive we will treat you for kala-azar first and then treat you for the HIV infection afterwards.

If you fulfill the national criteria for anti-retroviral therapy, we will provide you with anti-retroviral therapy for the duration of the project (18 months) or as required by national guidelines, at no cost to you.

If you do not wish to be tested for HIV, you will still benefit from the treatment for your kala-azar.

There is no obligation for you to accept the HIV test within this study, and if you refuse it, or do not wish to be informed of the results of your test, you will not be deprived of any other medical care that we offer you. You may wish to take time to think about being tested. If you change your mind later, and would like to be tested, we will do this for you at any time during this trial.

Consent Form:

I, the undersigned, confirm that, as I give consent to participate in the study, it is with a clear understanding of the objectives and conditions of the study and with the recognition of my right to withdraw from the study if I change my mind.

I ……………………….. do hereby give consent to Dr …………………………… to include me in the proposed research and the treatment. I have been given the necessary information and understand that there might be some risks involved in the treatment procedures. I have also been assured that I can withdraw my consent at any time without penalty or a loss of benefits. The proposal has been explained to me in the language I understand.

Name of patient : ______________________

Patient's Signature: _____________

Name of Doctor: ___________________________

Doctor's Signature:____________________

Date : __________________

Witness: __________________________Date:___________________

CONSENT FOR MINORS (UNDER 18 YRS)

I Mr/Ms ________________________________ being a person aged 18 years and over and being the Parent/Lawful guardian of master/miss______________________

hereby consent to Dr_____________________
to include Master/Miss _____________________________ in the intended research as explained and understood by me.

I have understood the implications, risks and immediate benefits of the tests and treatment to Master/Miss _______________________________.
I accept the tests and treatment to be carried out and the risks attached .

I understand that I have the right to withdraw
Master/Miss ________________________ from the research at any time, for any reason without penalty or harm. In case of withdrawal, I understand that the Physicians will continue to take care of Master/Miss
_________________________________ like any other patient.

All the above conditions have been explained to me in
____
language which I understand

___________________________________ Guardian's full name

___________________________________ Guardian's signature

Date:_____________

___________________________________ Child's full name

___________________________________ Person obtaining consent

___________________________________ Witness

Date:_____________

Consent Form for HIV testing:

I, the undersigned, confirm that, as I give consent to HIV testing, it is with a clear understanding of the objectives of HIV testing in this study,

the availability of counseling services,

the confidentiality of the test results

and in the case that I am positive for HIV, the possibility of receiving anti-retroviral therapy for the duration of the trial (18 months) should I fulfil the criteria set by the national guidelines

I ……………………….. hereby give consent to Dr ___________________

To perform this test.

I have been given the necessary information in a language that I understand.

Name of patient : ______________________

Patient's Signature: ____________

Name of Doctor: __________________________

Doctor's Signature:____________________

Date : _________________

Witness: _________________________Date:___________________

<u>Contact persons (to be customized for each study site)</u>

1. **Name and address of study site investigator**

2. **Name and address of next of kin of study patient**

3. **Name and address of Ethics Committee Chair**
