# Supplementary material for: Paromomycin for the Treatment of Visceral Leishmaniasis in Sudan: A Randomized, Open-Label, Dose-Finding Study
Source: PLoS Negl Trop Dis. 2010 Oct 26;4(10):e855. doi: 10.1371/journal.pntd.0000855 (PMC2964291; doi:10.1371/journal.pntd.0000855)
Supplement: Protocol S3 — Protocol amendment. (0.06 MB PDF) [file pntd.0000855.s004.pdf]

8th July 2005

Protocol Amendment on dosage and pharmacokinetic evaluation (PK) for paromomycin monotherapy arm of trial LEAP 0104 following preliminary analysis of efficacy data presented to a meeting of the Principal Investigators held at the Kenyan Medical Research Institute (KEMRI) Nairobi, Kenya on 20th and 21st June 2005

Title: A multicentre, randomised, comparative trial of efficacy and safety of sodium stibogluconate (SSG) versus paromomycin (PM) versus a combination of SSG and PM as first line treatment for visceral leishmaniasis (VL) in Ethiopia, Kenya and Sudan.

Reason for amendment

Initial results from the trial LEAP 0104 which commenced in November 2004, from 2 trial sites in Sudan (n = 90 Um el Kher, and n = 45 Kassab), indicate that paromomycin at a dosage of 15mg/kg/day for 21 days is less effective, for the treatment of acute, symptomatic treatment of visceral leishmaniasis, based on parasitological test of cure (TOC) at the end of treatment (day 22), by lymph node aspiration, in accordance with national VL policy in Sudan. **Table. 1.** This dosage may, however, be effective in Kenya (based on a small sample size, n=32)

Explanation for table 1:

Table 1: Complete Parasite Clearance by site and treatment

	**Combination**	**PM**	**SSG**	**P-Value**
**Um El Kher**	25 / 30 (83.3)	10 / 30 (33.3)	26 / 30 (86.7)	< 0.001
**Kassab**	12 / 15 (80.0)	9 / 15 (60.0)	14 / 15 (93.3)	0.113
**Kenya**	10 / 11 (90.9)	11 / 11 (100)	10 / 10 (100)	1.00
**P-Value***	.981	< 0.001	0. 571	

P-value from Fisher's exact test

* Column one shows that there were no significant inter-site differences in response to combination (p=0.981) and SSG (p=0.571)
Column two shows that there were significant differences in response to PM at the different sites (p < 0.001).

The majority of cases of VL in the disease endemic area of the Horn of Africa occur in Sudan, therefore it is considered essential to find a dose which is effective in Sudan if paromomycin is to be a useful alternative therapy for visceral leishmaniasis in this region.

No efficacy data are yet available from Ethiopia as final approval was received from the relevant Ethics and Regulatory agencies in that country only in May 2005 and recruitment only commenced in mid-June '05.

Safety and tolerability of the test drug paromomycin appears to be acceptable based on the data available from adverse and serious adverse event reports **Tables 2a and 2b.**

Table 2a: Adverse and serious adverse event reports

	1 GIT	2 Skin	3 RTI	4 Musculo skeletal	5 ENT	6 EYES	7 Systemic	8 Infection	9 Kidney	10 Liver	11 Heart
**Combined** **1 Not Related**	2	2	1		1	1	1	2	4		
**2 Unlikely**	1	2	1		2	1	2				
**3 Possible**			2	2			1			1	
**4 Probable**				5	1					3	
**Total**	**3**	**4**	**4**	**7**	**4**	**2**	**4**	**2**	**4**	**4**	
**PM** **1 Not Related**	6	3	3		4		2	8	4		
**2 Unlikely**	1	1		1	1		3				
**3 Possible**		1		4			1		2		
**4 Probable**							2			1	
**Total**	**7**	**5**	**3**	**5**	**5**	**0**	**8**	**8**	**6**	**1**	
**SSG** **1 Not Related**	2	3	9	2	9	3	2	10	2		
**2 Unlikely**	1	1	1	1	2				2		
**3 Possible**	1	1		1	3		1				
**4 Probable**		2		1			2		1		2
**Total**	**4**	**7**	**10**	**5**	**14**	**3**	**5**	**10**	**5**	**0**	**2**

Table 2b: SAEs

	1 GIT	2 Skin	3 RTI	4 Musculo skeletal	5 ENT	6 EYES	7 Systemic	8 Infection	9 Kidney	10 Liver	11 Heart
**Combined** **1 Not related**											
**2 Unlikely**											
**3 Possible**										1	
**4 Probable**											1
**Total**											
**PM** **1 Not Related**					1						
**2 Unlikely**											
**3 Possible**											
**4 Probable**											
**Total**											
**SSG** **1 Not Related**					1						
**2 Unlikely**											
**3 Possible**											
**4 Probable**									1		
**Total**					**2**				**1**	**1**	**1**

These results are in stark contrast to those obtained in a recent study of an apparently similar patient population with VL in India. (Shyam Sundar personal communication).

Table 3. Efficacy Results of Trial VLPM01
A randomised controlled trial of Paromomycin (PM) versus amphothericin B in patients with visceral leishmaniasis in Bihar, India. *(Ref. 8)*

	**PM** N=500	**Amphotericin B** N=116
**Initial Cure rate TOC at 4 weeks**	99%	98.8%
**Final Cure at 6 Months**	94.6%	98.8%

Only limited PK data in healthy volunteers are available, Kanyok et al 1997,**Table 4**.

Table 4. Pharmacokinetics of Paromomycin in Health Volunteers (Kanyok et al 1997.)

**RESULTS**		
	**12 mg / kg (N = 8)**	**15 mg / kg (N = 7)**
Cmax (µg/mL)	21.6	23.4
Tmax (h)	1.19	1.51
T ½ (h)	2.21	2.64
AUC	86.3	104.5
Renal Clearance	79.6	76.1
Recovery in time (24h)	67.8%	60.1%
Analytical technique	HPLC	

No data are yet available in patients from the study in India. It is therefore considered to be essential to include PK assessment in a limited number of patients, during the assessment of two further PM dosing schedules in Sudan. See appendix 1.

Previous studies in patients with VL over the last 15 years in both Africa (Kenya and Sudan) and in India have indicated that a dosage of 15mg/kg can be expected to be both effective and well tolerated. **Table 5** (-redrawn and with additional detail from Table 5 of the protocol of 31st July 2004.)

Table 5. Summary of clinical studies using PM

Drug and dosage schedules	Treatment Outcome TOC	Treatment Outcome DC	Statistic	References / Comments
**Combination therapy versus PM or SSG as a single-agent Tx**				
G1 = PM14-16 mg./kg/day 20days SSG20mg./kg/day 20days; (n = 23)	100%	87.0%	P<0.05 [G1 – G2]<br>P<0.01 [G1 – G3]	Ref: [1]<br>Clinical cure rate 100% in each group at end of treatment.<br>Epistaxis associated with SSG
G2= PM14-16 mg./kg/day 20days; (n = 19)	84.2%	79.0%	P<0.01 [G2 – G3]	
G3 =SSG20mg./kg/day 20days; [Standard Tx] (n = 11)	90.9%	54.5%	-	
**Combination therapy versus Standard SSG Tx**				
G1 = PM 15 mg./kg/day 17days, and SSG 20mg./kg/day 17days; (n = 61)	95.0% at 17 days	n.d.	P=0.039	Ref: [2]<br>Clinical cure rate 100% in both groups
G2 = SSG 20mg./kg/day; 30 days; (n = 60)	81.0% at 17 days	n.d.		
**Combination therapy versus Standard SSG Tx**				
G1 = PM 15 mg./kg/day 17days, and SSG 20mg./kg/day 17days; (n = 61)	95.0% at 30 days	n.d.	n.s.	Ref: [2]
G2 = SSG 20mg./kg/day; 30 days; [Standard Tx] (n = 60)	93.4% at 30 days	n.d		

Table 5. Summary of clinical studies using PM (table cont'd)

Drug and dosage schedules	Treatment Outcome TOC	DC	Statistic	References / Comments
PM 12mg + SSG 20mg<br>20 days<br>(n=24)	82%	82%	n.a.	Ref: [3]<br>2 deaths on Tx<br>- splenic rupture<br>- renal failure
G1a = PM 12mg + SSG 20mg<br>(n=32) 20 days<br>G1b = PM 12mg + SSG 10mg<br>(n= 32) 20 days<br>G1c = PM 12mg + SSG 5mg<br>(n=32) 20 days<br><br>G2a = PM 6mg + SSG 20mg<br>(n=13) 20 days<br>G2b = PM 6mg + SSG 10mg<br>(n=12) 20 days<br>G2c = PM 6mg + SSG 5mg<br>(n=13) 20 days	88%<br><br>71%<br><br>72%<br><br><br>69%<br><br>50%<br><br>46%	-<br><br>[80%]<br><br>-<br><br><br>-<br><br>-<br><br>-	n.a.	Ref: [4]<br><br>Only 50% attended FU.<br>5 relapses<br>8 slow responders<br><br>PM 6mg dosage groups stopped prematurely due to inefficacy<br><br>Safety:<br>- No renal toxicity<br>- Increase in liver enzymes common<br>- ECG normal<br>- Audiometry: data inadequate
**PM single agent therapy versus Standard SSG Tx**<br>G1 = PM 12 mg./kg/day<br>21days; (n = 30)<br><br>G2 = PM 16 mg./kg/day<br>21days; (n =30)<br><br>G3 = PM 20 mg./kg/day<br>21days; (n = 30)<br><br>G4 = SSG 20mg./kg/day<br>28days[Standard Tx]<br>(n = 30)	93.3%<br><br><br>100%<br><br><br>100%<br><br><br>73.3%	77%<br><br><br>93%<br><br><br>97%<br><br><br>63%	P = 0.26 [G1 – G4]<br><br><br>P<0.005 [G2 – G4]<br><br><br>P<0.005 [G3 – G4]<br><br><br>-	Ref: [5]<br><br>All treatments well tolerated<br>- No renal toxicity<br>- No ototoxicity<br>- No clinically relevant differences in laboratory values

Table 5. Summary of clinical studies using PM (table cont'd)

Drug and dosage schedules	Treatment Outcome TOC	DC	Statistic	References / Comments
**Combination therapy, and PM Dose finding**				
G1 = PM12 mg./kg/day 21days SSG20mg./kg/day 21days; (n = 52)	94.2%	92.3%	P<0.001 [G1 – G3]	Ref: [6]<br><br>1 case of myocarditis on SSG.<br><br>Audiometry data inadequate for analysis.<br><br>Lab values – no significant changes from baseline values.
G2 = PM18 mg./kg/day 21days SSG20mg./kg/day 21days; (n = 48)	95.8%	93.8%	P<0.001 [G2 – G3]	
G3 = SSG20mg./kg/day 30days; [Standard Tx] (n = 50)	55.1%	53.1%	-	
**PM Single-agent Tx (Dose finding) versus standard Tx**				
G1 = PM 12 mg./kg/day 21days; (n = 30)	100%	90.0%	P< 0.05 for G1 – G4 G2 – G4 G3 – G4	Ref: [7]<br><br>No important AEs reported.<br><br>No renal toxicity observed.<br><br>ECG and audiometry data inadequate for analysis.
G2 = PM 16 mg./kg/day 21days; (n = 30)	100%	88.9%		
G3 = PM 20 mg./kg/day 21days; (n = 30)	100%	86.2%		
G4 = SSG 20mg./kg/day 28days [Standard Tx] (n = 30)	73.3%	69.0%		

Abbreviations

TOC = Test of Cure at end of treatment
DC = Definitive Cure at 6 month follow up
n.s. = Not Significant
n.a. = Not Available
n.d. = Not Done

The only study from Africa of paromomycin monotherapy; Chunge et al which reported a TOC of 100% and a definitive cure (DC) at six months of 87% was performed 15 years ago in Kenya. Seaman et al, in Sudan in 1993 studied a combination of paromomycin and SSG and obtained a 95% TOC at day 30.
Studies in India from Thakur et al and Jha et al have consistently reported high test of cure with paromomycin monotherapy at doses ranging from 12 -20mg/kg/day but doses of 16-20mg/kg have been required to deliver definitive cure rates at six months of 90%.

Figure 1. Dose response for paromomycin – Phase II studies.

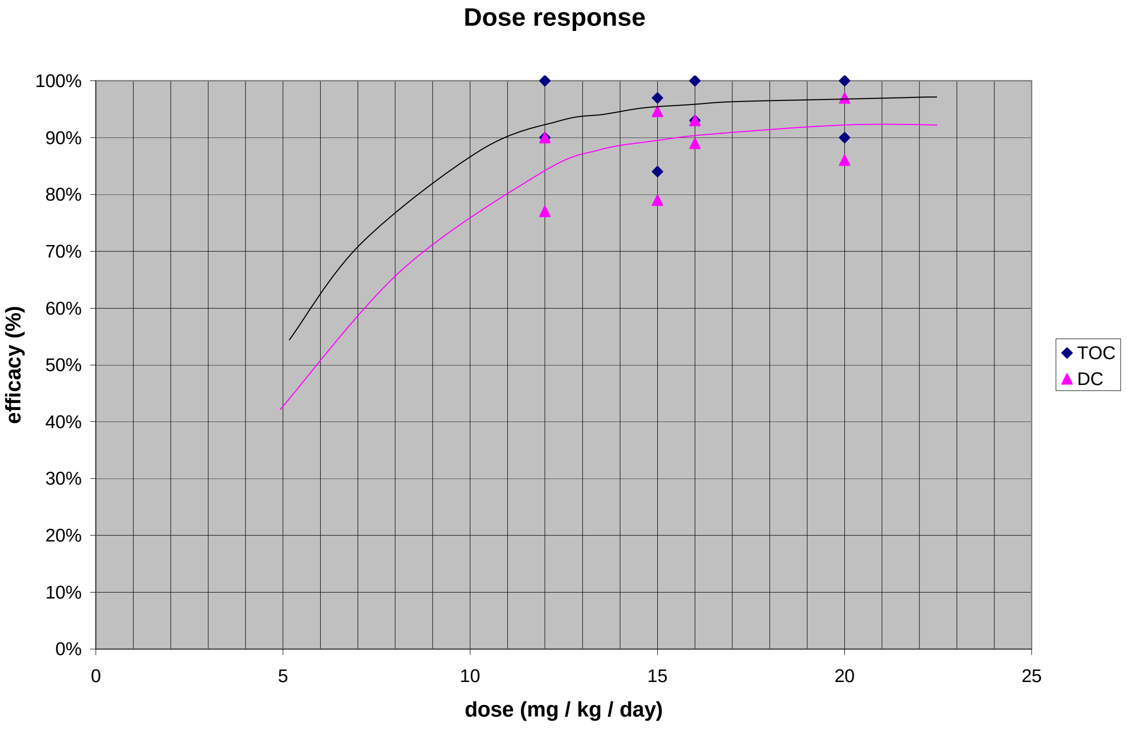

Tolerability and safety have been acceptable in all studies performed so far, using intramuscular injection as the mode of drug administration, with no significant renal toxicity. Only 3 cases of ototoxicity, (<1%) and all reversible, were seen in the large phase III trial from India of 500 patients (Sundar et al personal communication)
In the 20mg/kg studies of Jha et al 1995 and Thakur et al 2000, Jha reported 2 cases of ototoxicity, 1 reversible and one irreversible and Thakur had inadequate data for assessment of ototoxicity.
In contrast administration by **intravenous injection** in the 14-16mg dose range Scott et al 1992 in UK, triggered autotoxicity in 3 of 7 patients in a small case series of imported cases of VL in returned travellers (Table 5).

Based on the evidence above the following protocol amendment is proposed to further elucidate the failure of paromomycin in Sudan.

Group A: Paromomycin 20 mg/kg/day for a total treatment duration of 21 days
Group B: Paromomycin 15mg/kg/day for a treatment duration of 28 days

A total of 42 patients (21 in each treatment arm) will be allocated to receive one of two treatment regimens using a computer-generated randomisation list
The first six patients in each treatment group above, with a body weight of 30kg or more, will have additional venous blood and urine sampling for pharmacokinetic evaluation as outlined in **Appendix 1**.
Inclusion and exclusion criteria are otherwise unchanged from those in the original protocol. (pages 18-19). An amended consent form will be used **Appendix 2.**

Patients who are symptomatic and HIV positive *will be excluded from participation in this protocol amendment but will be offered treatment with SSG and rescue with Ambisome in the event of treatment failure.*

Efficacy The two treatment regimens A and B above will be assessed for efficacy in the 42 patients on the basis of parasitological test of cure following **bone marrow aspiration on day 29 or 30**, this test being acknowledged to be more sensitive but also more invasive and potentially more painful than lymph node aspiration.
Note: Splenic aspiration for parasitological assessment of VL is not allowed in Sudan.

Rescue. Patients who fail to respond to treatment will be given rescue therapy with AmBisome as in the original protocol (Page 24)

Safety will be assessed using the same weekly assessment tests as in the original protocol LEAP 0104 of 31st July 2004 (page 26) -all patients will have ECG and audiometry assessments!

It is expected that following completion of this 42 patient amendment an effective dosing regimen for paromomycin monotherapy in Sudan will have been identified.
This regimen will then be substituted for the current paromomycin only treatment arm, in the 3 arm comparison of the original protocol. Sample size may need to be re-calculated in the light of this new information when it becomes available. A further amendment will be submitted to Ethics Committees and Regulatory Authorities.

Other trial sites

During the period of recruitment and treatment for the 42 patients above at Kassab, Sudan, the trial will be put on hold at KEMRI, Nairobi, Kenya, but recruitment will continue in Ethiopia to n=45 at each trial site (Gondar and Arba Minch hospitals) to obtain contemporaneous information of efficacy and safety of paromomycin at a dosage of 15mg/kg/day for 21 days, as it cannot be assumed that patient response in Ethiopia will match either that in Sudan or Kenya.

The trial site at Um el Kher has been closed by Medecins sans Frontieres (MSF) for operational reasons unrelated to this trial, and will not be available for future patient recruitment to the trial.

Trial site key personnel and monitor update as at June 2005 (can be found in Appendix 3):

References

1. Chunge, C.N., et al., *Treatment of visceral leishmaniasis in Kenya by aminosidine alone or combined with sodium stibogluconate.* Trans R Soc Trop Med Hyg, 1990. 84(2): p. 221-5.
2. Seaman, J., et al., *Epidemic visceral leishmaniasis in Sudan: a randomized trial of aminosidine plus sodium stibogluconate versus sodium stibogluconate alone.* J Infect Dis, 1993. 168(3): p. 715-20.
3. Thakur, C.P., et al., *Treatment of visceral leishmaniasis (kala-azar) with aminosidine (= paromomycin)-antimonial combinations, a pilot study in Bihar, India.* Trans R Soc Trop Med Hyg, 1992. 86(6): p. 615-6.
4. Thakur, C.P., et al., *Aminosidine plus sodium stibogluconate for the treatment of Indian kala-azar: a randomized dose-finding clinical trial.* Trans R Soc Trop Med Hyg, 1995. 89(2): p. 219-23.
5. Jha, T.K., et al., *Randomised controlled trial of aminosidine (paromomycin) v sodium stibogluconate for treating visceral leishmaniasis in North Bihar, India.* Bmj, 1998. 316(7139): p. 1200-5.
6. Thakur, C.P., et al., *A prospective randomized, comparative, open-label trial of the safety and efficacy of paromomycin (aminosidine) plus sodium stibogluconate versus sodium stibogluconate alone for the treatment of visceral leishmaniasis.* Trans R Soc Trop Med Hyg, 2000. 94(4): p. 429-31.
7. Thakur, C.P., et al., *Treatment of visceral leishmaniasis with injectable paromomycin (aminosidine). An open-label randomized phase-II clinical study.* Trans R Soc Trop Med Hyg, 2000. 94(4): p. 432-3.
8. Sundar, S., *Randomised, comparative trial of paromomycin versus amphotericin B in Bihar, India.* Personal communication, 2005.
9. Scott, J.A., et al., *Aminosidine (paromomycin) in the treatment of leishmaniasis imported into the United Kingdom.* Trans R Soc Trop Med Hyg, 1992. 86(6): p. 617-9.
10. Kanyok, T.P., et al., *Pharmacokinetics of intramuscularly administered aminosidine in healthy subjects.* Antimicrob Agents Chemother, 1997. 41(5): p. 982-6.

8th July 2005

**Principal Investigators**	**SIGNATURE**	**DATE**
Dr Monique Wasunna<br>(Trial Medical Coordinator)	……………………	
Prof. Asrat Hailu	……………………	
Dr. Getahun Mengistu	……………………	
Dr. Musa A Mudawi	……………………	
Dr. Manica Balasegaram	……………………	
**Trial Manager**		
Dr Juma Rashid	……………………	
**Trial Statistician**		
Mr. Lawrence Muthami	……………………	
**Project Manager**		
Dr Catherine Royce	……………………	

8th July 2005

Appendix 1

Pharmacokinetic sampling schedule for patients with a body weight of 30kg or more who consent to participate in the PK part of the study.

Venous sampling for PK patients in group A (n=12)

Day 1 of treatment:
At time 0, 0.25, 0.5, 1.0, 2, 4, 6, 8, 12, and 24 hours after dosing

Day 14 of treatment;
At time 0 before the day 14 dose 0.25, 0.5, 1.0, 2, 4, 6, 8, 12, and 24 hours post dosing.

Venous sampling for patients in group B (n=12)

Day 1 of treatment:
At time 0, 0.25, 0.5, 1.0, 2, 4, 6, 8, 12, and 24 hours after dosing

Day 26 of treatment;

At time 0 before the day 26 dose 0.25, 0.5, 1.0, 2, 4, 6, 8, 12, and 24 hours post dosing.

5 ml of blood at each sampling point will be taken to ensure sufficient plasma is obtained for duplicate test analysis.
Total blood volume taken for PK on each sampling day is 50ml.

Urine sampling

Both groups A and B will also have 24 hour urine collections on PK venous sampling days, collected in aliquots 0-2h, 2-4h, 4-6h, 6-8h, 8-12 and 12-24h.

Safety assessments

Safety assessments (blood biochemistry, haematology, urinalysis, ECG and audiometry,) will be performed as in the original LEAP 0104 protocol before treatment, on day 7, 14 for both groups and additionally on day 22 for group A and day 26 for group B so that direct correlations can be made with the PK data.

Appendix 2

Consent form for Patients participating in PK sampling.

Appendix 3

Site Investigators:

Ethiopia:

Gondar:
Dr Nurelign Gashu (Site PI)
Dr Asfaweseen
Dr Sisay Yifru

Abar Minch:
Dr Samson Tesfaye (Site PI)
Dr Gabriell Teklu

Kenya:
Dr M Wasunna
Dr J R Rashid
Dr Jane Mbui
Dr F Kirui

Sudan:
Dr. Ahmed Musa Mudawi
Prof. E A G Khalil
Dr. Osama M ahmed

Monitors:
Dr Shibru Berhanu
Dr Hilda O'hara
